# T3P-Promoted Synthesis of a Series of 2-Aryl-3-phenyl-2,3-dihydro-4*H*-pyrido[3,2-*e*][1,3]thiazin-4-ones and Their Activity against the Kinetoplastid Parasite *Trypanosoma brucei*

**DOI:** 10.3390/molecules26206099

**Published:** 2021-10-09

**Authors:** Lee J. Silverberg, Tapas K. Mal, Carlos N. Pacheco, Megan L. Povelones, Madeline F. Malfara, Anthony F. Lagalante, Mark A. Olsen, Hemant P. Yennawar, Hany F. Sobhi, Kayla R. Baney, Robin L. Bozeman, Craig S. Eroh, Michael J. Fleming, Tracy L. Garcia, Casey L. Gregory, Julia E. Hahn, Alyssa M. Hatter, Lexi L. Johns, Tianna L. Klinger, Jennie J. Li, Andrew J. Menig, Grace C. Muench, Melissa E. Ramirez, Jordyn Reilly, Nicole Sacco, Alexandra M. Sheidy, Marla M. Stoner, Eric N. Thompson, Soroush F. Yazdani

**Affiliations:** 1 Schuylkill Campus, Pennsylvania State University, 200 University Drive, Schuylkill Haven, PA 17972, USA; krb5791@psu.edu (K.R.B.); Woweezowee13@yahoo.com (R.L.B.); craig.eroh@gmail.com (C.S.E.); mjf5883@gmail.com (M.J.F.); tvg5225@psu.edu (T.L.G.); caseygregory@vt.edu (C.L.G.); jeh5702@psu.edu (J.E.H.); amh.psu19@gmail.com (A.M.H.); luj54@psu.edu (L.L.J.); tiannaklinger@icloud.com (T.L.K.); jul564@psu.edu (J.J.L.); ajm7716@psu.edu (A.J.M.); gzm5297@psu.edu (G.C.M.); MELI88986@GMAIL.COM (M.E.R.); jordynpreilly@gmail.com (J.R.); nicolesacco25@gmail.com (N.S.); aps5448@psu.edu (A.M.S.); marla.stoner07@gmail.com (M.M.S.); ent5068@psu.edu (E.N.T.); SOROUSHY9@YAHOO.COM (S.F.Y.); 2 Department of Chemistry, Pennsylvania State University, 104 Chemistry Building, Room 08, University Park, State College, PA 16802, USA; tkm9@psu.edu (T.K.M.); carlos.n.pacheco@njit.edu (C.N.P.); 3 Chemistry and Environmental Science, New Jersey Institute of Technology, Tiernan Hall, Room B006, University Heights, Newark, NJ 07102, USA; 4 Mendel Science Center, Department of Biology, Villanova University, 800 Lancaster Avenue, Villanova, PA 19085, USA; megan.povelones@villanova.edu (M.L.P.); madeline.malfara@villanova.edu (M.F.M.); 5 Mendel Science Center, Department of Chemistry, Villanova University, 800 Lancaster Avenue, Villanova, PA 19085, USA; anthony.lagalante@villanova.edu (A.F.L.); mark.olsen@villanova.edu (M.A.O.); 6 Department of Biochemistry and Molecular Biology, Pennsylvania State University, 8 Althouse Laboratory, University Park, State College, PA 16802, USA; hpy1@psu.ede; 7 Center for Organic Synthesis Coppin State University, Room-229, 2500 West North Avenue, Baltimore, MD 21216, USA; Hsobhi@coppin.edu

**Keywords:** pyridothiazinone, T3P, imine, *N*-aryl, thionicotinic acid, parasites, *T. brucei*

## Abstract

A series of fourteen 2-aryl-3-phenyl-2,3-dihydro-4*H*-pyrido[3,2-*e*][1,3]thiazin-4-ones was prepared at room temperature by T3P-mediated cyclization of *N*-phenyl-*C*-aryl imines with thionicotinic acid, two difficult substrates. The reactions were operationally simple, did not require specialized equipment or anhydrous solvents, could be performed as either two or three component reactions, and gave moderate–good yields as high as 63%. This provides ready access to *N*-phenyl compounds in this family, which have been generally difficult to prepare. As part of the study, the first crystal structure of neutral thionicotinic acid is also reported, and showed the molecule to be in the form of the thione tautomer. Additionally, the synthesized compounds were tested against *T. brucei,* the causative agent of Human African Sleeping Sickness. Screening at 50 µM concentration showed that five of the compounds strongly inhibited growth and killed parasites.

## 1. Introduction

**Figure 1:**
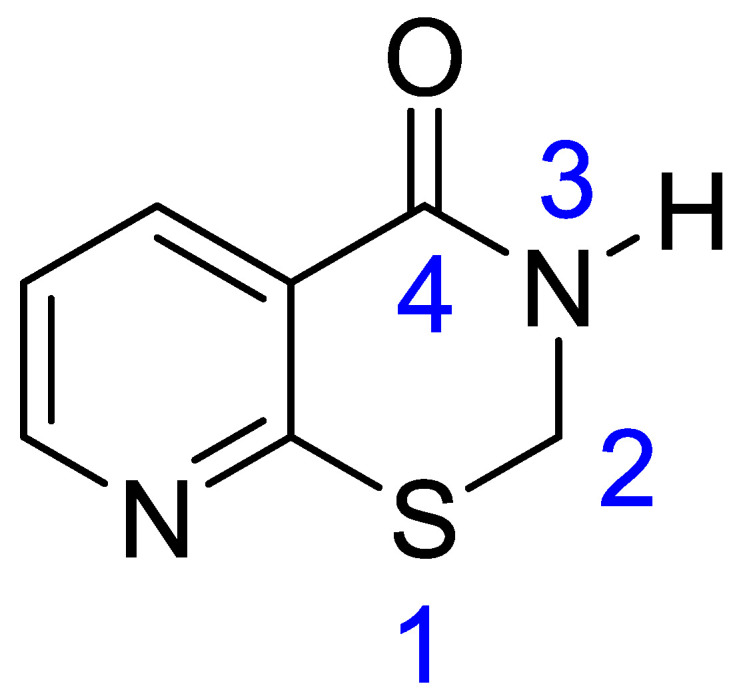
The 2,3-dihydro-4*H*-pyrido[3,2-*e*][1,3]thiazin-4-one scaffold.

**Scheme 1:**
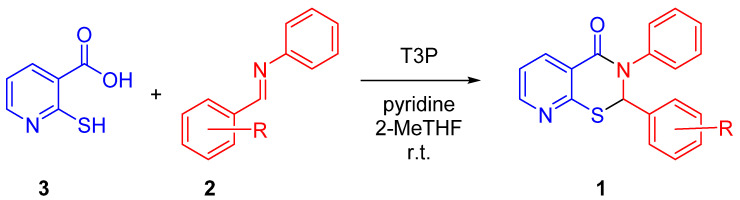
Preparation of 2-aryl-3-phenyl-2,3-dihydro-4*H*-pyrido[3,2-*e*][1,3]thiazin-4-ones **1**.

The 2,3-dihydro-4*H*-pyrido[3,2-*e*][1,3]thiazin-4-one scaffold (Figure 1) features a pyridine ring fused to a thiazine ring. Compounds with this scaffold have previously shown anticancer [1], antibacterial [2], and glycosidase inhibitory bioactivity [3]. A compound previously reported by us, 2,3-diphenyl-2,3-dihydro-4*H*-pyrido[3,2-*e*][1,3]thiazin-4-one **1j** (Scheme 1, Table 1, R = H) [4,5], inhibited growth of two kinetoplastid parasites, *Trypanosoma brucei* and *Crithidia fasciculata* [6]. The effect was particularly striking for *T. brucei*, which causes disease in humans and domesticated livestock. Parasite cell growth was inhibited at a specific stage of the cell cycle, indicating that the completion of cytokinesis was delayed or prevented in treated cells. *T. brucei* parasites treated with this compound also had delayed mitochondrial division and showed signs of an endocytosis defect, both of which could disrupt cytokinesis. Although the IC50s were in the micromolar range, precluding further investigation of **1j** as an antikinetoplastid therapeutic, these parasites represent a powerful system with which to discover the biological target(s) of this and related compounds, having a great deal of conservation with other eukaryotes and a variety of tools for genetic manipulation and screening. In addition, these compounds may serve as a method to probe the highly orchestrated cell cycle and cell biology of these important pathogens.

Compound **1j** has also been shown to have activity against two human pathogenic fungi, *Cryptococcus neoformans* and *Lomentospora prolificans* [7], although this work is still ongoing. For potential use as a drug, analogs of lead compound **1j** may show enhanced activity and lower IC50s. We are thus interested in producing derivatives of **1j** to establish structure–activity relationships and improve the efficacy. 

There are only a modest number of 2,3-dihydro-4*H*-pyrido[3,2-*e*][1,3]thiazin-4-ones reported in the literature, and the number grows quite small when there is an aryl group on the nitrogen of the 1,3-thiazin-4-one ring [1,2,8,9,10]. A common method used for preparation of a variety of 2,3-dihydro-4*H*-1,3-thiazin-4-ones is the cyclization of an imine with a 3-thiocarboxylic acid [11]. However, it is known that *N*-aryl imines are less reactive in the preparation of similar 2,3-dihydro-4*H*-1,3-thiazin-4-ones than *N*-alkyl imines [12,13,14]. Furthermore, Dandia et al. reported in 2004 that reaction of *N*-aryl imines with thionicotinic acid (2-mercaptopyridine-3-carboxylic acid) **3** by thermal methods with various catalysts and solvents failed, which they attributed to the “low reactivity” of **3** [9]. Dandia et al. succeeded in their reactions by using microwaves at 132–145 °C [9]. Arya et al. have reported success with ZSM-5-([MIM]^+^BF_4_^−^), a Brønsted acid ionic liquid catalyst, plus ultrasonication at 95 °C [1,8]. 

Where both of these methods involved specialized equipment (microwave oven, ultrasonic processor) and high temperature, we have previously reported simple, room temperature synthesis of various 2,3-diaryl-2,3-dihydro-4*H*-1,3-thiazin-4-ones [5,12,13,14], including a few 2-aryl-3-phenyl-2,3-dihydro-4*H*-pyrido[3,2-*e*][1,3]thiazin-4-ones [4,5,15], using amide coupling agent 2,4,6-tripropyl-1,3,5,2,4,6-trioxatriphosphorinane-2,4,6-trioxide (T3P). T3P has several advantages over other amide-forming reagents, including that the reagent is safe, the by-products are water soluble and easily removed, and in combination with pyridine epimerization at the carbon alpha to the carboxylic acid is minimized [16]. Taylor’s group has also used T3P to prepare 2,3-dihydro-4*H*-1,3-thiazin-4-ones [17,18,19], but have not reported preparation of any 2,3-dihydro-4*H*-pyrido[3,2-*e*][1,3]thiazin-4-ones.

Herein we report the synthesis of a full series of 2-aryl-3-phenyl-2,3-dihydro-4*H*-pyrido[3,2-*e*][1,3]thiazin-4-ones **1**, with a variety of substituents in the *meta* and *para* positions on the *C*-aryl ring, as well as one in the *ortho* position (Scheme 1). Both electron-withdrawing and electron-donating substituents were used. Additionally, initial screening of the compounds against *T. brucei* is disclosed. The first X-ray crystallographic structure of neutral reagent **3** is also reported. 

## 2. Results and Discussion

The compounds **1a**–**1n** were prepared and the yields are shown in Table 1. The preparations of the imines **2a**–**2n** used have been previously reported [14], but an improved procedure for *m*-F imine **2i** is included here. In cases where the imine was a liquid (**2g**, **2l**, and **2n**), the reactions were performed as three-component couplings, forming the imine in situ from an aldehyde and aniline rather than preparing it separately. As we have reported previously in the similar reaction of 3-mercaptopropionic acid [12], yields were expected to be a little lower for the three-component reaction than for the two-component reaction. Equimolar amounts of imine **2** (or aldehyde and aniline) and thionicotinic acid **3** were used, with excesses of T3P and pyridine. Reactions were allowed to proceed at least overnight and followed by TLC. Compounds **1a [15]**, **1h [15]**, and **1j [4,5]** have been previously reported, but here updated procedures and yields are provided. All of the reactions attempted gave desired products **1a**–**n**. In general, these compounds were readily crystallized. Yields ranged from 22% (*ortho*-nitro **1c**) to 63% and averaged 46%. In the three-component reactions (**1g, 1l**, and **1n**) the average yield was 40%, ranging from 35–44%. The two-component reactions (**1a**–**1f**, **1h**–**1k**, **1m**) ranged from 22–63% and averaged 48% yield. 

Arya et al. reported yields of 80–94% in reactions of *N*-aryl imines with **3** using ultrasonication and a Bronsted acid ionic liquid at 95 °C [1,8]. The same group reported similar reactions in 85–92% yield using microwaves at 132–145 °C [9]. Thus, while the yields were high, the reactions required specialized equipment and high temperatures. The method reported here is run at room temperature without special equipment, is simple to perform, and does not require dry solvents.

**Scheme 2:**
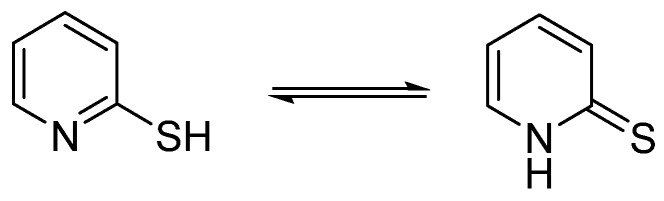
Tautomerization of 2-mercaptopyridine [20].

**Scheme 3:**
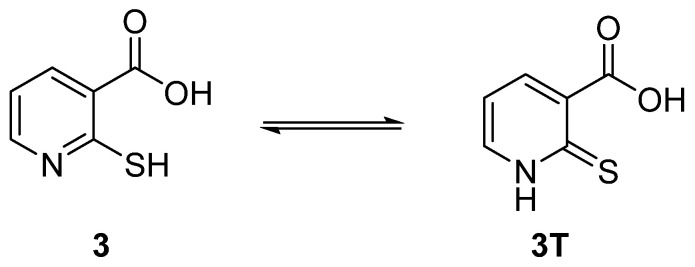
Tautomerization of aromatic **3** to nonaromatic **3T**.

**Figure 2:**
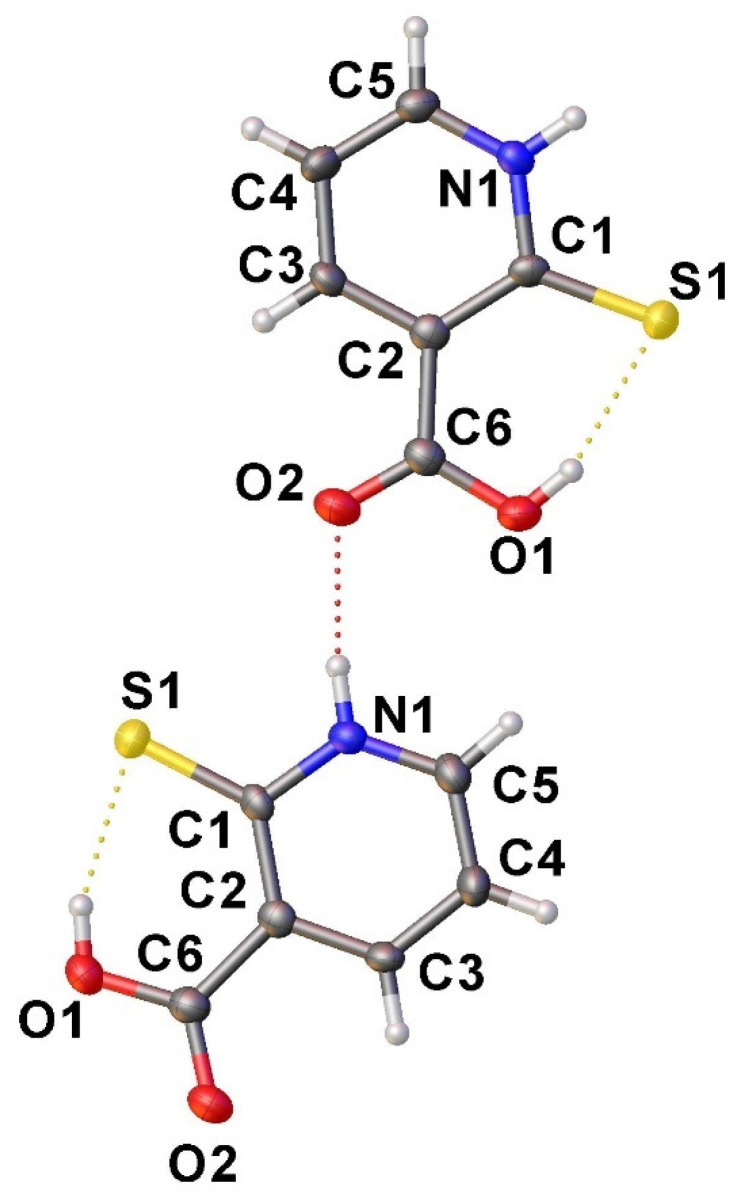
Drawing of the X-ray crystal structure of **3T**. There is intramolecular O1-H---S1 hydrogen bonding and intermolecular N1-H---O2 hydrogen bonding.

**Figure 3:**
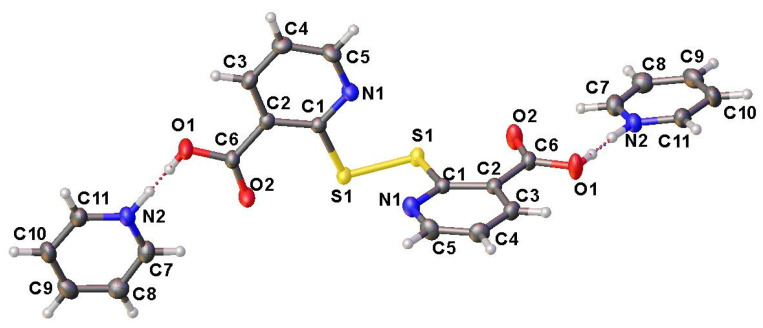
Drawing of the X-ray crystal structure of the disulfide pyridine solvate from **3**. Half of the molecular species in the crystal have the proton on the carboxylic acid oxygen (O1) and the other half have it on the pyridine (solvent) nitrogen (N2).

Reaction of the same *meta*- and *para*-substituted imines **2a, 2b, 2d**–**2n** with thiosalicylic acid (2-mercaptobenzoic acid), an aromatic thioacid, had ranged from 12–43% with an average yield of 30% [14]. The more reactive aliphatic 3-mercaptopropionic acid gave yields of 33–75%, averaging 58% [12]. The yields for **3** (35–63%, average 48%) thus fall in between these two—better than thiosalicylic acid but not as good as 3-mercaptopropionic acid. Compound **3** is more complex than it might appear. 2-Mercaptopyridine is known to tautomerize to a nonaromatic thione (Scheme 2) [20]. However, Saleh studied **3** and proposed that the carboxylic acid deprotonates more readily than the thiol [21]. Smith and Sagatys determined the X-ray crystallographic structure of ammonium 2-mercaptopyridine-3-carboxylate hydrate and found a zwitterion in which both the thiol and carboxylic acid were deprotonated and the nitrogen was protonated [22]. Crystal structures have also been reported in which **3** was oxidized to the disulfide [23,24]. This left open several possibilities for precisely how **3** is behaving in the reaction reported herein. To study this further, two crystal studies were undertaken. Growth of crystals of **3** from methanol gave crystals in which **3** was in the form of the thione tautomer **3T** (Scheme 3, Figure 2). This appears to be the first time that the structure of the neutral molecule has been determined and shows that the thiol is in fact deprotonated before the carboxylic acid. There is O1-H---S1 hydrogen bonding within the molecule, and N1-H---O2 hydrogen bonding between molecules (Figure 2). Growth of **3** from pyridine, which is part of the reaction medium, produced the disulfide as a pyridine solvate (Figure 3). Half of the molecular species in the crystal have the proton on the carboxylic acid O and the other half have it on the pyridine (solvent) N, suggesting a proton exchange equilibrium in solution. Disulfide formation presumably occurred by O_2_ oxidation [24], as the crystals were grown by slow evaporation to the atmosphere. The reaction reported here has been run under N_2_, although air has not been rigorously excluded. Thus, it would appear that the reaction may have an equilibrium between the aromatic **3** and the nonaromatic **3T** (Scheme 3), which could account for the yields being between those of aromatic thiosalicylic acid and nonaromatic 3-mercaptopropionic acid.

**Scheme 4:**
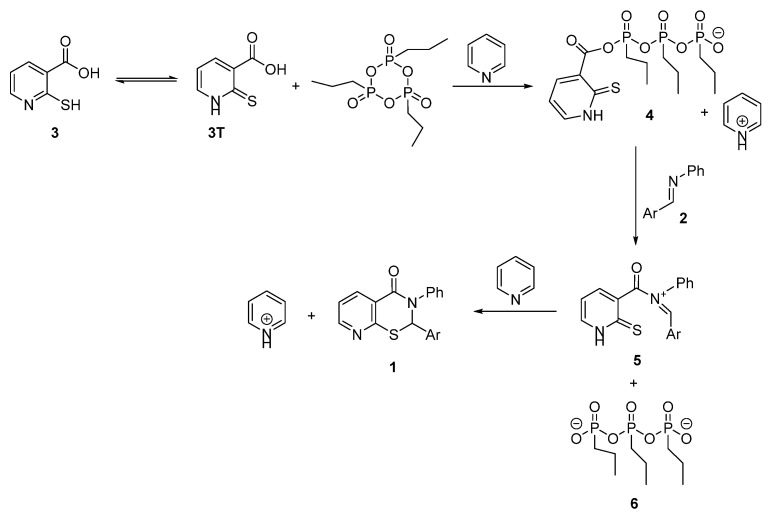
Possible reaction pathway.

Based on these results and mechanisms previously proposed by us [12,14] and Unsworth et al. [17], one possible pathway for the reaction is shown in Scheme 4. In the proposed mechanism, the carboxylate of **3T** attacks a phosphorus of T3P, opening the ring and forming a phosphonate ester **4**. The nitrogen of imine **2** then attacks the carbonyl of **4**, with the phosphonate group **6** leaving, to yield the iminium ion **5**. Finally, ring closure by nucleophilic attack by sulfur on the strongly electrophilic carbon of the iminium ion **5** along with deprotonation by pyridine produces **1**. Considering this last step, it would be expected that electron-withdrawing groups on the *C*-aryl ring would increase the electrophilicity of the iminium ion carbon. However, no pattern was discernible in the yields with regard to electron-withdrawing or electron-donating. This may be because the slow step in the process is likely to be the amide bond formation, and the subsequent ring closure is rapid. The low yield of **1c** can be attributed to steric hindrance by the *ortho* substituent.

**Figure 4:**
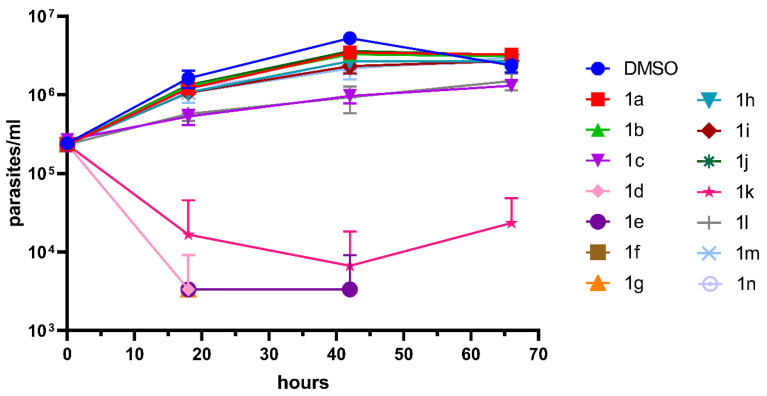
Compounds **1a**–**1n** were tested against long slender bloodstream form *Trypanosoma brucei.* Treated cells (50 µM) were compared to a vehicle control (DMSO) over the course of three days. The average of three replicates was plotted for treated cells with standard deviation shown as error bars. DMSO (vehicle) was plotted as the average of four replicates. Cell densities below the level of detection of the hemacytometer (10^4^) are represented by 0, and are not shown on the graph. For example, **1f** was not detectable at 18 h, **1d** and **1g** (overlapping on the graph) were not detectable at 42 h, and **1e** was not detectable at 66 h. A table of the raw data is in the Supplementary Material.

All fourteen compounds **1a**–**1n** were tested at 50 µM concentration against the kinetoplastid parasite *T. brucei brucei.* Cultured long slender bloodstream form *T. brucei* were used for these experiments, as this form most closely represents the stage present in the mammalian host. Of the fourteen compounds analyzed, including **1j** that was previously shown to inhibit *T. brucei* at higher concentrations [6], compounds with trifluoromethyl substitution (**1d**, **1e**), bromo substitution (**1f**, **1g**), and *para*-methyl substitution (**1k**) strongly inhibited growth of trypanosomes within the first 18 h (Figure 4; a table of the raw data is in the Supplementary Material). In fact, these compounds not only inhibited growth but killed parasites, with the *para*-bromo substitution (**1f**) killing parasites the quickest. Interestingly, the *para*-methyl substitution (**1k**) strongly inhibited parasite growth, while the *meta*-methyl substitution (**1l**) only showed a subtle effect on parasite growth. This may indicate that the substituent in the *para*-position may be able to bind more favorably than the *meta*-position, although more studies would be needed to confirm this. In summary, compounds **1d**, **1e**, **1f**, **1g**, and **1k** strongly inhibited parasite growth at a concentration of 50 µM and are good candidates for further study. 

X-ray crystallographic structures of compounds **1a**, **1h**, and **1j** have been previously reported [4,15], as have physical and spectroscopic properties of **1j** [5]. Here, full spectral data and physical properties of **1a**–**1i** and **1k**–**1n** are provided in the Materials and Methods Section and in the Supplementary Material.

## 3. Materials and Methods

**General:** Thionicotinic acid (2-mercaptopyridine-3-carboxylic acid) **3** was obtained from Oakwood and also purchased from Sigma-Aldrich. 2-Methyltetrahydrofuran and silica gel for flash chromatography were purchased from Sigma-Aldrich. Pyridine was purchased from AlfaAesar. 2,4,6-Tripropyl-1,3,5,2,4,6-trioxatriphosphorinane-2,4,6-trioxide (T3P) in 2-methyltetrahydrofuran (50 weight %) was obtained from Curia. TLC plates (silica gel GF, 250-micron, 10 × 20 cm, cat. No. 21521) were purchased from Analtech. TLCs were visualized under short wave UV, and then with I_2_, and then by spraying with ceric ammonium nitrate/sulfuric acid and heating. Infrared spectra of **1e**, **1f**, **1g**, **1h**, and **1n** were run on a Thermo-Fisher NICOLET iS50 FT-IR using a diamond-ATR attachment for direct powder analysis (Coppin State University). Infrared spectra of **1a, 1b, 1c, 1d, 1i, 1k,** and **1m** were run on another Thermo-Fisher NICOLET iS50 FT-IR using a diamond-ATR attachment for direct powder analysis (Penn State Schuylkill). ^1^H, ^13^C NMR, and ^19^F NMR experiments (Penn State’s shared NMR facility, University Park) were carried out on a Bruker Avance-III-HD 500.20-MHz (^1^H frequency) instrument using a 5 mm Prodigy (liquid nitrogen cooled) BBO BB-1H/19F/D Z-GRD cryoprobe. Samples were dissolved in CDCl_3_ and analyzed at RT. Typical conditions for ^1^H acquisition were 2 s relaxation delay, acquisition time of 4.089 s, spectral width of 8 kHz, 16 scans. Spectra were zero-filled to 128k points, and multiplied by exponential multiplication (EM with LB = 0.3 Hz) prior to FT. For ^13^C experiments, data were acquired with power-gated ^1^H decoupling using a 2 s relaxation delay, with acquisition time of 1.1 s, spectral width of 29.8 kHz, and 256 scans. Spectra were zero-filled once, and multiplied by EM with LB = 2 Hz prior to FT. ^19^F NMR data were acquired with acquisition time of 0.58 s, spectral width of 240 ppm, 32 scans, and relaxation delay of 2 s. Exact mass of synthesized compounds was determined LC-MS (Villanova University). Exact mass was measured on a SCIEX Exion LC with a SCIEX 5600+ TripleTOF MS. Separation was achieved on an Agilent Infinity LabPoroshell 120 EC-C18 column maintained at 40 °C with a gradient of 90/10 (water/acetonitrile with 0.1% formic acid) ramped from 5/95 over 6 min at a flowrate of 0.5 mL/min. The TOF-MS was scanned over 100–500 Da and calibrated with the SCIEX APCI positive calibrant solution prior to accurate mass analysis. Compound exact mass was measured in positive ESI mode with a DP = 100 V, CE = 10, GAS1 = GAS2 = 60 psi, CUR = 30 psi, ISV = 5500 V, and source temperature of 450 °C. Melting points were performed on an Arthur H. Thomas Co. Thomas Hoover Capillary Melting Point Apparatus or on a Vernier Melt Station (Penn State Schuylkill). Single crystals of C_6_H_5_NO_2_S and C_12_H_7_N_2_O_4_S_2_•2(C_5_H_5.5_ N) were grown by slow evaporation of methanol and pyridine solutions, respectively. Suitable crystals were selected and sequentially mounted using a nylon loop and a dab of paratone oil on a Rigaku Oxford diffraction, Synergy Custom system, HyPix-Arc 150 diffractometer (Penn State University Park). The crystals were at 173(2) K during data collection. Using Olex2 [25], the structures were solved with the SHELXT [26] structure solution program using Intrinsic Phasing and refined with the SHELXL [27] refinement package using least-squares minimization.

### X-ray Crystallographic Data

**Thionicotinic acid grown in methanol:** Crystal Data for C*_6_*H*_5_*NO*_2_*S (M = 155.17 g/mol): orthorhombic, space group Pna21 (no. 33), a = 8.2935(4) Å, b = 13.2553(6) Å, c = 5.8995(3) Å, V = 648.55(5) Å3, Z = 4, T = 173(2) K, μ(Cu Kα) = 3.882 mm^−1^, D*_calc_* = 1.589 g/cm*^3^*, 1840 reflections measured (12.59° ≤ 2Θ ≤ 147.864°), 988 unique (R*_int_* = 0.0168, R*_sigma_* = 0.0162) which were used in all calculations. The final R1 was 0.0544 (I > 2σ(I)) and wR2 was 0.1385 (all data). 

**Thionicotinic acid grown in pyridine:** Crystal Data for C_12_H_7_N_2_O_4_S_2_•2(C_5_H_5.5_N) (M = 466.52 g/mol): monoclinic, space group C2/c (no. 15), a = 7.9786(2) Å, b = 12.3830(3) Å, c = 21.5361(5) Å, β = 96.019(2)°, V = 2116.02(9) Å3, Z = 4, T = 173(2) K, μ(Cu Kα) = 2.616 mm^−1^, D_calc_ = 1.464 g/cm^3^, 5474 reflections measured (8.256° ≤ 2Θ ≤ 149.326°), 2034 unique (R_int_ = 0.0159, R_sigma_ = 0.0133) which were used in all calculations. The final R1 was 0.0310 (I > 2σ(I)) and wR2 was 0.0819 (all data). (Note that in the cif, the molecular formula and weight listed (C_11_H_9_N_2_SO_2_ and 233.26) is half of that for the moiety, corresponding to the contents in the asymmetric unit.)

**Imines:** Imines **2** were prepared as previously reported [14]. One purification was updated as reported below.

***N*-{[3-(Trifluoromethyl)phenyl]methylidene}aniline (2e)**: Recrystallization from hexanes provided light yellow crystals (9.6692 g, 78%). mp: 45.6–47.8 °C (lit. 47–47.5 °C [28]).

**General Procedure for Three-Component Coupling to Prepare2-Aryl-3-phenyl-2,3-dihydro-4*H*-pyrido[3,2-*e*][1,3]thiazin-4-ones:** A two-necked 25 mL round-bottom flask was oven-dried, cooled under N_2_, and charged with a stir bar. Aniline (0.559 g, 6 mmol) and a substituted benzaldehyde (6 mmol) were added. 2-Methyltetrahydrofuran (2.3 mL) was added and the solution was stirred. Thionicotinic acid (0.931 g, 6 mmol) was added. Pyridine (2.9 mL, 36 mmol) was added. Finally, 2,4,6-tripropyl-1,3,5,2,4,6-trioxatriphosphorinane-2,4,6-trioxide (T3P) in 2-methyltetrahydrofuran (50 weight percent; 11 mL, 18 mmol) was added. The reaction was stirred at room temperature and followed by TLC, then poured into a separatory funnel with dichloromethane (20 mL). The mixture was washed with water (10 mL). The aqueous was then extracted twice with dichloromethane (10 mL each). The organics were combined and washed with saturated sodium bicarbonate (10 mL) and then saturated sodium chloride (10 mL). The organic was dried over sodium sulfate and concentrated under vacuum to give a crude mixture. Further purification was carried out as indicated below for each compound.

**2-(3-Bromophenyl)-3-phenyl-2,3-dihydro-4*H*-pyrido[3,2-*e*][1,3]thiazin-4-one (1g):** After chromatography on 30 g silica gel with mixtures of ethyl acetate and hexanes, recrystallization from ethyl acetate and hexanes gave off-white crystals (1.0001 g, 42% yield). mp: 142.6–143.5 °C. ^1^H NMR (CDCl_3_): δ(ppm): δ 8.53 (dd, ^3^*J*_HH_ 4.8, ^4^*J*_HH_ 1.9 Hz, 1H, *para* to pyridine N), 8.48 (dd, ^3^*J*_HH_ 7.9, ^4^*J*_HH_ 1.9 Hz, 1H, *ortho* to pyridine **N**), 7.63 (s, 1H, Ar), 7.47–7.37 (m, 4H, Ar), 7.34 (d, ^3^*J*_HH_ 9.0 Hz, 3H, Ar), 7.24 (dd, ^3^*J*_HH_ 7.9, 4.8 Hz, 1H, *meta* to pyridine N), 7.18 (t, ^3^*J*_HH_ 7.9 Hz, 1H, Ar), 6.12 (s, 1H, C2-H). ^13^C NMR (CDCl_3_): δ(ppm): δ 163.1 (C=O), 156.2 (Ar), 153.0 (Ar), 141.8 (Ar), 141.5 (Ar), 138.1 (Ar), 131.8 (Ar), 130.1 (Ar), 129.7 (Ar), 129.5 (Ar), 127.7 (Ar), 126.0 (Ar), 125.6 (Ar), 125.1 (Ar), 123.0 (Ar), 121.5 (Ar), 64.3 (C2). HRMS (*m/z*): [C_19_H_13_BrN_2_OS+H]^+^ of 397.0001 is consistent with calculated [M+H]^+^ of 397.0005. IR (neat, cm^−1^): 1667 (C=O)*. R*_f_ (50% EtOAc/hexanes) = 0.52.

**2-(3-Methylphenyl)-3-phenyl-2,3-dihydro-4*H*-pyrido[3,2-*e*][1,3]thiazin-4-one (1l):** After chromatography on 30 g silica gel with mixtures of ethyl acetate and hexanes, recrystallization from ethyl acetate and hexanes gave off-white crystals (0.6939 g, 35% yield). mp: 114.8–116.2 °C. ^1^H NMR (CDCl_3_): δ(ppm): δ 8.55–8.40 (m, 2H, *ortho* and *para* to pyridine N), 7.41 (t, ^3^*J*_HH_ 7.8 Hz, 2H, Ar), 7.35 (d, ^3^*J*_HH_ 8.4 Hz, 2H, Ar), 7.33–7.23 (m, 4H, Ar), 7.23–7.13 (m, 2H, Ar), 7.08 (d, ^3^*J*_HH_ 7.6 Hz, 1H, Ar), 6.13 (s, 1H, C2-H), 2.31 (s, 3H, CH_3_). ^13^C NMR (CDCl_3_): δ(ppm): δ 163.4 (C=O), 156.8 (Ar), 152.8 (Ar), 142.0 (Ar), 139.1 (Ar), 138.5 (Ar), 137.9 (Ar), 129.4 (Ar), 129.3 (Ar), 128.4 (Ar), 127.4 (Ar), 127.3 (Ar), 126.1 (Ar), 125.7 (Ar), 123.6 (Ar), 121.2 (Ar), 64.9 (C2), 21.5 (CH_3_). HRMS (*m*/*z*): [C_20_H_16_N_2_OS+H]^+^ of 333.1067 is consistent with calculated [M+H]^+^ of 333.1061. IR (neat, cm^−1^): 1662 (C=O)*. R*_f_ (50% EtOAc/hexanes) = 0.57.

**2-(3-Methoxyphenyl)-3-phenyl-2,3-dihydro-4*H*-pyrido[3,2-*e*][1,3]thiazin-4-one (1n):** After chromatography on 30 g silica gel with mixtures of ethyl acetate and hexanes, recrystallization from ethyl acetate and hexanes gave an off-white powder (0.9294 g, 44% yield). mp: 153.0–153.6 °C. ^1^H NMR (CDCl_3_): δ(ppm): δ 8.51 (dd, ^3^*J*_HH_ 4.8, ^4^*J*_HH_ 1.9 Hz, 1H, *para* to pyridine N), 8.47 (dd, ^3^*J*_HH_ 7.8, ^4^*J*_HH_ 1.9 Hz, 1H, *ortho* to pyridine N), 7.41 (t, ^3^*J*_HH_ 7.8 Hz, 2H, Ar), 7.36 (d, ^3^*J*_HH_ 7.0 Hz, 2H, Ar), 7.31 (t, ^3^*J*_HH_ 7.3 Hz, 1H, Ar), 7.24–7.18 (m, 2H, Ar), 7.04 (d, ^3^*J*_HH_ 7.7 Hz, 1H, Ar), 6.99 (s, 1H, Ar), 6.81 (dd, ^3^*J*_HH_ 8.2, ^4^*J*_HH_ 2.7 Hz, 1H, Ar), 6.13 (s, 1H, C2-H), 3.76 (s, 3H, OCH_3_). ^13^C NMR (CDCl_3_): δ(ppm): δ 163.3 (C=O), 159.8 (Ar), 156.7 (Ar), 152.8 (Ar), 142.0 (Ar), 140.8 (Ar), 138.0 (Ar), 129.6 (Ar), 129.3 (Ar), 127.5 (Ar), 126.2 (Ar), 125.7 (Ar), 121.3 (Ar), 118.9 (Ar), 114.0 (Ar), 112.5 (Ar), 64.9 (C2), 55.3 (OCH_3_). HRMS (*m/z*): [C_20_H_16_N_2_O_2_S+H]^+^ of 349.1008 is consistent with calculated [M+H]^+^ of 349.1005. IR (neat, cm^−1^): 1661 (C=O). *R*_f_ (50% EtOAc/hexanes) = 0.52.

**General Procedure for Two-Component Coupling to Prepare2-Aryl-3-phenyl-2,3-dihydro-4*H*-pyrido[3,2-*e*][1,3]thiazin-4-ones:** A two-necked 25 mL round-bottom flask was oven-dried, cooled under N_2_, and charged with a stir bar, an imine (6 mmol), and thionicotinic acid (0.931 g, 6 mmol). 2-Methyltetrahydrofuran (2.3 mL) was added and the solution was stirred. Pyridine (1.95 mL, 24 mmol) was added. Finally, 2,4,6-tripropyl-1,3,5,2,4,6-trioxatriphosphorinane-2,4,6-trioxide (T3P) in 2-methyltetrahydrofuran (50 weight percent; 7.3 mL, 12 mmol) was added. The reaction was stirred at room temperature and followed by TLC, then poured into a separatory funnel with dichloromethane (20 mL). The mixture was washed with water (10 mL). The aqueous was then extracted twice with dichloromethane (10 mL each). The organics were combined and washed with saturated sodium bicarbonate (10 mL) and then saturated sodium chloride (10 mL). The organic was dried over sodium sulfate and concentrated under vacuum to give a crude mixture. Further purification was carried out as indicated below for each compound.

**2-(4-Nitrophenyl)-3-phenyl-2,3-dihydro-4*H*-pyrido[3,2-*e*][1,3]thiazin-4-one (1a)**: After chromatography on 30 g silica gel with mixtures of ethyl acetate and hexanes, recrystallization from ethyl acetate and hexanes gave an off-white powder (0.9726 g, 45% yield). mp: 190.0–191.3 °C. ^1^H NMR (CDCl_3_): δ(ppm): δ 8.53 (dd, ^3^*J*_HH_ 4.8, ^4^*J*_HH_ 1.0 Hz, 1H, *para* to pyridine N), 8.48 (d, ^3^*J*_HH_ 7.9 Hz, 1H, *ortho* to pyridine N), 8.18 (d, ^3^*J*_HH_ 8.9 Hz, 2H, Ar), 7.68 (d, ^3^*J*_HH_ 9.1 Hz, 2H, Ar), 7.44 (t, ^3^*J*_HH_ Hz, 2H, Ar), 7.39–7.30 (m, 3H, Ar), 7.26 (dd, ^3^*J*_HH_ 7.9, 4.8 Hz, 1H, *meta* to pyridine N), 6.23 (s, 1H, C2-H). ^13^C NMR (CDCl_3_): δ(ppm): 163.0 (C=O), 155.8 (pyridine ring), 153.1 (pyridine ring), 147.9 (Ar), 146.4 (Ar), 141.6 (Ar), 138.3 (Ar), 129.6 (Ar), 127.9 (Ar), 127.5 (Ar), 125.9(Ar), 125.5 (Ar), 124.0 (Ar), 121.8 (Ar), 64.3 (C2). HRMS (*m*/*z*): [C_19_H_13_N_3_O_3_S+H]^+^ of 364.0751 is consistent with calculated [M+H]^+^ of 364.0756. IR (neat, cm^−1^): 1652 (C=O). *R*_f_ (50% EtOAc/hexanes) = 0.50.

**2-(3-Nitrophenyl)-3-phenyl-2,3-dihydro-4*H*-pyrido[3,2-*e*][1,3]thiazin-4-one (1b)**: After chromatography on 30 g silica gel with mixtures of ethyl acetate and hexanes, recrystallization from a mixture of ethyl acetate and hexanes gave a white solid (1.128 g, 52% yield). mp: 149.7–149.9 °C. ^1^H NMR (CDCl_3_): δ(ppm): δ 8.55–8.52 (m, 1H, *para* to pyridine N), 8.50 (dd, ^3^*J*_HH_ 7.8, ^4^*J*_HH_ 1.6 Hz, 1H, *ortho* to pyridine N), 8.37 (s, 1H, Ar), 8.15 (d, ^3^*J*_HH_ 8.3 Hz, 1H, Ar), 7.82 (d, ^3^*J*_HH_ 8.9 Hz, 1H, Ar), 7.51 (t, ^3^*J*_HH_ 8.0 Hz, 1H, Ar), 7.45 (t, ^3^*J*_HH_ 7.9 Hz, 2H, Ar), 7.39–7.32 (m, 3H, Ar), 7.26 (dd, ^3^*J*_HH_ 7.8, 4.8 Hz, 1H, *meta* to pyridine N), 6.25 (s, 1H, C2-H). ^13^C NMR (CDCl_3_): δ(ppm): 162.9 (C=O), 155.8 (Ar), 153.1 (Ar), 148.4 (Ar), 141.7 (Ar), 141.6 (Ar), 138.3 (Ar), 132.1 (Ar), 129.8 (Ar), 129.7 (Ar), 127.9 (Ar), 125.9 (Ar), 125.7 (Ar), 123.7 (Ar), 121.8 (Ar), 121.6 (Ar), 64.2 (C2). HRMS (*m/z*): [C_19_H_13_N_3_O_3_S+H]^+^ of 364.0754 is consistent with calculated [M+H]^+^ of 364.0756. IR (neat, cm^−1^): 1654 (C=O). *R*_f_ (50% EtOAc/hexanes) = 0.30.

**2-(2-Nitrophenyl)-3-phenyl-2,3-dihydro-4*H*-pyrido[3,2-*e*][1,3]thiazin-4-one (1c)**: After chromatography on 30 g silica gel with mixtures of ethyl acetate and hexanes, recrystallization from a mixture of ethyl acetate and hexanes gave light yellow crystals (0.4811 g, 22% yield). mp: 195.7–1196.4 °C. ^1^H NMR (CDCl_3_): δ(ppm): δ 8.52 (q, ^4^*J*_HH_ 1.9 Hz, 1H, *para* to pyridine N), 8.51 (s, 1H, Ar), 8.18 (dd, ^3^*J*_HH_ 8.1, ^4^*J*_HH_ 1.5 Hz, 1H, Ar), 7.64 (d ^3^*J*_HH_ 7.0 Hz, 1H, Ar), 7.57 (td, ^3^*J*_HH_ 7.6, ^4^*J*_HH_ 1.5 Hz, 1H, Ar), 7.53–7.46 (m, 1H, Ar), 7.47–7.40 (m, 2H, Ar), 7.38–7.30 (m, 3H, Ar), 7.26–7.21 (m, 1H, Ar), 7.15 (s, 1H, C2-H). ^13^C NMR (CDCl_3_): δ(ppm): 163.9 (C=O), 156.4 (Ar), 153.2 (Ar), 146.2 (Ar), 142.0 (Ar), 138.1 (Ar), 135.4 (Ar), 133.4 (Ar), 129.7 (Ar), 129.6 (Ar), 128.0 (Ar), 127.5 (Ar), 127.1 (Ar), 125.8 (Ar), 124.9 (Ar), 121.4 (Ar), 60.6 (C2). HRMS (*m/z*): [C_19_H_13_N_3_O_3_S+H]^+^ of 364.0750 is consistent with calculated [M+H]^+^ of 364.0756. IR (neat, cm^−1^): 1663 (C=O). *R*_f_ (50% EtOAc/hexanes) = 0.49.

**3-Phenyl-2-[4-(trifluoromethyl)phenyl]-2,3-dihydro-4*H*-pyrido[3,2-*e*][1,3]thiazin-4-one (1d):** After chromatography on 30 g silica gel with mixtures of ethyl acetate and hexanes, recrystallization from ethyl acetate and hexanes gave slightly off-white crystals (1.4097 g, 63% yield). mp: 185.2–186.1 °C. ^1^H NMR (CDCl_3_): δ(ppm): δ 8.52 (dd, ^3^*J*_HH_ 4.8, ^4^*J*_HH_ 1.8 Hz, 1H, *para* to pyridine N), 8.48 (dd, ^3^*J*_HH_ 7.9, ^4^*J*_HH_ 1.9 Hz, 1H, *ortho* to pyridine N), 7.69–7.54 (m, 4H, Ar), 7.49–7.39 (m, 2H, Ar), 7.39–7.31 (m, 3H, Ar), 7.24 (dd, ^3^*J*_HH_ 7.9, 4.8 Hz, 1H, *meta* to pyridine N), 6.20 (s, 1H, C2-H). ^13^C NMR (CDCl_3_): δ(ppm): δ 163.0 (C=O), 156.0 (Ar), 152.8 (Ar), 143.2 (Ar), 141.7 (Ar), 138.4 (Ar), [131.3, 131.0, 130.7, 130.5, q, ^2^*J*_CF_ 32.6 Hz, Ar C connected to CF_3_), 129.6 (Ar), 127.8 (Ar), 126.9 (Ar), 126.0 (Ar), [125.8, 125.8, 125.8, 125.7, q, ^3^*J*_CF_ 3.7 Hz, Ar *ortho* to CF_3_), 125.5 (Ar), [(one peak possibly underneath the peak at 126.9), 124.8 (Ar), 122.6 (Ar), 120.4, q, ^1^*J*_CF_ 272.5 Hz, CF_3_], 121.6 (Ar), 64.4 (C2). ^19^F NMR (CDCl_3_): δ(ppm): δ -62.8. HRMS (*m/z*): [C_20_H_13_F_3_N_2_OS+H]^+^ of 387.0775 is consistent with calculated [M+H]^+^ of 387.0778. IR (neat, cm^−1^): 1652 (C=O). *R*_f_ (50% EtOAc/hexanes) = 0.54.

**3-Phenyl-2-[3-(trifluoromethyl)phenyl]-2,3-dihydro-4*H*-pyrido[3,2-*e*][1,3]thiazin-4-one (1e):** After chromatography on 30 g silica gel with mixtures of ethyl acetate and hexanes, recrystallization from ethyl acetate and hexanes gave a white powder (1.2574 g, 54% yield). mp: 119.2–120.2 °C. ^1^H NMR (CDCl_3_): δ(ppm): δ 8.52 (dd, ^3^*J*_HH_ 4.8, ^4^*J*_HH_ 1.9 Hz, 1H, *para* to pyridine N), 8.48 (dd, ^3^*J*_HH_ 7.9, ^4^*J*_HH_ 1.9 Hz, 1H, *ortho* to pyridine N), 7.73 (s, 1H, Ar), 7.67 (d, ^3^*J*_HH_ 7.8 Hz, 1H, Ar), 7.55 (d, ^3^*J*_HH_ 7.9 Hz, 1H, Ar), 7.44 (td, ^3^*J*_HH_ 8.2, 5.2 Hz, 3H, Ar), 7.37–7.30 (m, 3H, Ar), 7.24 (dd, ^3^*J*_HH_ 7.9, 4.8 Hz, 1H, *meta* to pyridine N), 6.21 (s, 1H, C2-H). ^13^C NMR (CDCl_3_): δ(ppm): δ 163.1 (C=O), 156.1 (Ar), 153.0 (Ar), 141.7 (Ar), 140.4 (Ar), 138.1 (Ar), [131.6, 131.4, 131.1, 130.9, q, ^2^*J*_CF_ 32.7 Hz, Ar C connected to CF_3_], 129.6 (Ar), 129.5 (Ar), 129.2 (Ar), 127.8 (Ar), [126.9, 124.7, 122.5, 120.4, q, ^1^*J*_CF_ 272.6 Hz, CF_3_], 126.0 (Ar), 125.7 (Ar), [125.6, 125.6, 125.5, 125.5, q, ^3^*J*_CF_ 3.9 Hz, Ar C *ortho* to CF_3_], [123.5, 123.5, 123.4, 123.4, q, ^3^*J*_CF_ 3.8 Hz, Ar C *ortho* to CF_3_], 121.6 (Ar), 64.5 (C2). ^19^F NMR (CDCl_3_): δ(ppm): δ -62.8. HRMS (*m/z*): [C_20_H_13_F_3_N_2_OS+H]^+^ of 387.0773 is consistent with calculated [M+H]^+^ of 387.0778. IR (neat, cm^−1^): 1653 (C=O). *R*_f_ (50% EtOAc/hexanes) = 0.59.

**2-(4-Bromophenyl)-3-phenyl-2,3-dihydro-4*H*-pyrido[3,2-*e*][1,3]thiazin-4-one (1f):** After chromatography on 30 g silica gel with mixtures of ethyl acetate and hexanes, recrystallization from ethyl acetate and hexanes gave off-white crystals (0.9455 g, 40% yield). mp: 171.6–173.6 °C. ^1^H NMR (CDCl_3_): δ(ppm): δ 8.59–8.39 (m, 2H, *ortho* and *para* to pyridine N), 8.18 (d, ^3^*J*_HH_ 8.8 Hz, 2H, Ar), 7.68 (d, ^3^*J*_HH_ 8.9 Hz, 2H, Ar), 7.44 (t, ^3^*J*_HH_ 7.8 Hz, 2H, Ar), 7.39–7.31 (m, 3H, Ar), 7.26 (dd, ^3^*J*_HH_ 7.9, 4.8 Hz, 2H, Ar), 6.23 (s, 1H, C2-H). ^13^C NMR (CDCl_3_): δ(ppm): δ 163.2 (C=O), 156.4 (Ar), 152.9 (Ar), 141.8 (Ar), 138.3 (Ar), 138.1 (Ar), 131.8 (Ar), 129.5 (Ar), 128.2 (Ar), 127.6 (Ar), 126.0 (Ar), 125.6 (Ar), 122.8 (Ar), 121.5 (Ar), 64.5 (C2). HRMS (*m/z*): [C_19_H_13_BrN_2_OS+H]^+^ of 397.0009 is consistent with calculated [M+H]^+^ of 397.0005. IR (neat, cm^−1^): 1652 (C=O). *R*_f_ (50% EtOAc/hexanes) = 0.59.

**2-(4-Fluorophenyl)-3-phenyl-2,3-dihydro-4*H*-pyrido[3,2-*e*][1,3]thiazin-4-one (1h):** After chromatography on 30 g silica gel with mixtures of ethyl acetate and hexanes, recrystallization from ethyl acetate and hexanes gave white crystals (1.0067 g, 50% yield). mp: 127.8–128.4 °C. ^1^H NMR (CDCl_3_): δ(ppm): δ 8.52 (dd, ^3^*J*_HH_ 4.8, ^4^*J*_HH_ 2.2 Hz, 1H, *para* to pyridine N), 8.46 (dd, ^3^*J*_HH_ 7.8, ^4^*J*_HH_ 2.2 Hz, 1H, *ortho* to pyridine N), 7.48–7.39 (m, 3H, Ar), 7.34 (d, ^3^*J*_HH_ 8.0 Hz, 2H, Ar), 7.26–7.16 (m, 2H, Ar), 6.99 (t, ^3^*J*_HH_ 8.6 Hz, 2H, Ar), 6.17 (s, 1H, C2-H). ^13^C NMR (CDCl_3_): δ(ppm): δ [163.5, 161.8, d, ^1^*J*_CF_ 248.7 Hz, C-F)) 163.3 (C=O), 156.6 (Ar), 152.9 (Ar), 141.8 (Ar), 138.0 (Ar), 137.9 (Ar), [134.9, 134.9, d, ^4^*J*_CF_ 3.3 Hz, C *para* to F], 129.4 (Ar), 129.1 (Ar), [128.5, 128.4, d, ^3^*J*_CF_ 8.8 Hz, C *meta* to F], 128.2, 127.6, 126.0, 125.7, 125.3 (Ar), 121.4 (Ar), [115.8, 115.6, d, ^2^*J*_CF_ 21.5 Hz, C *ortho* to F], 64.5 (C2). ^19^F NMR (CDCl_3_): δ(ppm): δ −112.79 (dq, ^3^*J*_HF_ 8.9, ^4^*J*_HF_ 4.5 Hz). HRMS (*m/z*): [C_19_H_13_FN_2_OS+H]^+^ of 337.0812 is consistent with calculated [M+H]^+^ of 337.0805. IR (neat, cm^−1^): 1652 (C=O). *R*_f_ (50% EtOAc/hexanes) = 0.48.

**2-(3-Fluorophenyl)-3-phenyl-2,3-dihydro-4*H*-pyrido[3,2-*e*][1,3]thiazin-4-one (1i):** After chromatography on 30 g silica gel with mixtures of ethyl acetate and hexanes, recrystallization from ethyl acetate and hexanes gave an off-white solid (0.8775 g, 43% yield). mp: 118.8–121.2 °C. ^1^H NMR (CDCl_3_): δ(ppm): δ 8.52 (dd, ^3^*J*_HH_ 4.8, ^4^*J*_HH_ 1.9 Hz, 1H, *para* to pyridine N), 8.47 (dd, ^3^*J*_HH_ 7.9, ^4^*J*_HH_ 1.9 Hz, 1H, *ortho* to pyridine N), 7.46–7.40 (m, 2H, Ar), 7.40–7.26 (m, 6H, Ar), 7.24 (dd, ^3^*J*_HH_ 7.9, 4.8 Hz, 1H, *meta* to pyridine N), 7.20 (dd, ^3^*J*_HH_ 10.3, ^4^*J*_HH_ 2.4 Hz, 1H, Ar), 7.01–6.95 (m, 1H, Ar), 6.15 (s, 1H, C2-H).^13^C NMR (CDCl_3_): δ(ppm): δ [163.8, 161.8, d, ^1^*J*_CF_ 248.0 Hz, C-F], 163.1 (C=O), 156.3 (Ar), 152.9 (Ar), [141.9, 141.9, d, ^3^*J*_CF_ 6.4 Hz, C *meta* to F], 141.8 (Ar), 138.2 (Ar), [130.3, 130.2, d, ^3^*J*_CF_ 8.2 Hz, C *meta* to F], 129.5 (Ar), 127.6 (Ar), 126.0 (Ar), 125.6 (Ar), [122.3, 122.3, d, ^4^*J*_CF_ 3.2 Hz, C *para* to F], 121.5, [115.8, 115.7, d, ^2^*J*_CF_ 21.3 Hz, C *ortho* to F], [114.0, 113.8 d, ^2^*J*_CF_ 23.6 Hz, C *ortho* to F], 64.4 (C2). ^19^F NMR (CDCl_3_): δ(ppm): δ −111.41 (td, ^3^*J*_HF_ 8.6, ^3^*J*_HF_ 7.7, ^4^*J*_HF_ 4.8 Hz). HRMS (*m/z*): [C_19_H_13_FN_2_OS+H]^+^ of 337.0806 is consistent with calculated [M+H]^+^ of 337.0805. IR (neat, cm^−1^): 1646 (C=O)*. R*_f_ (50 % EtOAc/hexanes) = 0.51.

**2,3-Diphenyl-2,3-dihydro-4*H*-pyrido[3,2-*e*][1,3]thiazin-4-one (1j):** After chromatography on 30 g silica gel with mixtures of ethyl acetate and hexanes, recrystallization from 2-propanol gave an ivory solid (0.9114 g, 48%). Data previously reported [5].

**2-(4-Methylphenyl)-3-phenyl-2,3-dihydro-4*H*-pyrido[3,2-*e*][1,3]thiazin-4-one (1k):** Recrystallization from 2-propanol gave an orange–yellow solid (1.0572 g, 53% yield). mp: 128–130 °C. ^1^H NMR (CDCl_3_): δ(ppm): δ 8.61–8.35 (m, 2H, *ortho* and *para* to pyridine N), 7.47–7.25 (m, 8H, Ar), 7.25–7.16 (m, 1H, Ar), 7.10 (d, ^3^*J*_HH_ 8.0 Hz, 2H, Ar), 6.15 (s, 1H, C2-H), 2.29 (s, 3H, CH_3_). ^13^C NMR (CDCl_3_): δ(ppm): δ 163.3 (C=O), 156.7 (Ar), 152.4 (Ar), 141.9 (Ar), 138.6 (Ar), 138.2 (Ar), 136.0 (Ar), 129.4 (Ar), 127.4 (Ar), 126.2 (Ar), 125.7 (Ar), 121.2 (Ar), 64.9 (C2), 21.0 (CH_3_). HRMS (*m/z*): [C_20_H_16_N_2_OS+H]^+^ of 333.1054 is consistent with calculated [M+H]^+^ of 333.1061. IR (neat, cm^−1^): 1657 (C=O). *R*_f_ (50% EtOAc/hexanes) = 0.49.

**2-(4-Methoxyphenyl)-3-phenyl-2,3-dihydro-4*H*-pyrido[3,2-*e*][1,3]thiazin-4-one (1m):** After chromatography on 30 g silica gel with mixtures of ethyl acetate and hexanes, recrystallization from 2-propanol gave a yellow solid (1.1419 g, 55% yield). mp: 142.5–143.0 °C. ^1^H NMR (CDCl_3_): δ(ppm): δ 8.51 (dd, ^3^*J*_HH_ 4.7, ^4^*J*_HH_ 1.8 Hz, 1H, *para* to pyridine N), 8.46 (dd, ^3^*J*_HH_ 7.9, ^4^*J*_HH_ 1.9 Hz, 1H, *ortho* to pyridine N), 7.43–7.30 (m, 7H, Ar), 7.21 (dd, ^3^*J*_HH_ 7.9, 4.8 Hz, 1H, *meta* to pyridine N), 6.81 (d, ^3^*J*_HH_ 8.9 Hz, 2H, Ar), 6.15 (s, 1H, C2-H), 3.77 (s, 3H, OCH_3_). ^13^C NMR (CDCl_3_): δ(ppm): δ 163.4 (C=O), 159.7 (Ar), 156.9 (Ar), 152.8 (Ar), 141.9 (Ar), 137.9 (Ar), 130.9 (Ar), 129.3 (Ar), 127.9 (Ar), 127.4 (Ar), 126.1 (Ar), 125.7 (Ar), 121.2 (Ar), 114.0 (Ar), 64.7 (C2), 55.3 (OCH_3_). HRMS (*m/z*): [C_20_H_16_N_2_O_2_S+H]^+^ of 349.1006 is consistent with calculated [M+H]^+^ of 349.1005. IR (neat, cm^−1^): 1644 (C=O). *R*_f_ (50% EtOAc/hexanes) = 0.48.

**Cell Culture:** Bloodstream form *T.* *brucei* *brucei* strain 90–13 were cultured at 37 °C in 5% CO_2_ in HMI-9 supplemented with 15% fetal bovine serum [29]. Cells were maintained in 5 µg/mL hygromycin and 1 µg/mL neomycin (G418). Parasites from a mid-log culture were split back to 2 × 10^5^ cells/mL in 1 mL, treated with 50 µM of each compound in triplicate, and counted over 3 days using a hemacytometer. The average of 4 replicates for the DMSO vehicle control and 3 replicates for treated samples was plotted with standard deviation shown as error bars (Figure 4).

## 4. Conclusions

A new, simple preparation of 2-aryl-3-phenyl-2,3-dihydro-4*H*-pyrido[3,2-e ][1,3]thiazin-4-ones 1 from two difficult substrates (2 and 3) has been demonstrated. The reactions run at room temperature, are simple to perform, and do not require dry solvents or special equipment such as a microwave oven or an ultrasonicator. A series of compounds with various electron-donating and electron-withdrawing groups on the C2-aryl ring was prepared in moderate–good yields. The reactions can be run as either a two- or three-component reaction, although yields were somewhat lower for the three-component reactions. 

Some of these compounds strongly inhibited growth of the parasite *T. brucei*, an important animal pathogen closely related to other species and subspecies that cause disease in humans. These compounds are good candidates for further study as pharmacological agents.

X-ray crystallographic studies of **3** yielded the first crystal structure of the neutral species and showed it to be in the thione form **3T**.

## Figures and Tables

**Table 1:** Compounds prepared as shown in Scheme 1.

Compound	R	Yield of 1 from 2 (%)
**1a** [15]	*p*-NO_2_	45% ^a^
**1b**	*m*-NO_2_	52% ^a^
**1c**	*o*-NO_2_	22% ^a^
**1d**	*p*-CF_3_	63% ^a^
**1e**	*m*-CF_3_	54% ^a^
**1f**	*p*-Br	40% ^a^
**1g**	*m*-Br	42% ^a,b^
**1h** [15]	*p*-F	50% ^a^
**1i**	*m*-F	43% ^a^
**1j** [4,5]	H	48% ^a^
**1k**	*p*-Me	53% ^c^
**1l**	*m*-Me	35% ^a,b^
**1m**	*p*-OMe	55% ^a^
**1n**	*m*-OMe	44% ^a,b^

^a^ Isolated by chromatography and subsequent recrystallization. ^b^ Performed as a three-component coupling rather than premaking the imine. Yield is from aniline. ^c^ Isolated by recrystallization.

## Data Availability

Data sharing not applicable.

## Supplementary Materials

The following are available online. ^1^H NMR, ^13^C NMR, ^19^F NMR, FTIR, Mass spectra, and data for the biological study are available online. CCDC deposition number 2108518–2108519 contains the supplementary crystallographic data for this paper. These data can be obtained free of charge via http://www.ccdc.cam.ac.uk/conts/retrieving.html, accessed on 7 August 2021 (or from the CCDC, 12 Union Road, Cambridge CB2 1EZ, UK; Fax: +44-1223-336033; E-mail: deposit@ccdc.cam.ac.uk).

## Sample Availability

Samples of the compounds are not available from the authors.
