# Supplementary material for: T3P-Promoted Synthesis of a Series of 2-Aryl-3-phenyl-2,3-dihydro-4H-pyrido[3,2-e][1,3]thiazin-4-ones and Their Activity against the Kinetoplastid Parasite Trypanosoma brucei
Source: Molecules. 2021 Oct 9;26(20):6099. doi: 10.3390/molecules26206099 (PMC8540051; doi:10.3390/molecules26206099)
Supplement: Supplementary file 1 [file molecules-26-06099-s001.zip › molecules-1353197-supplementary.pdf]

T3P-Promoted Synthesis of a Series of 2-Aryl-3-phenyl-2,3-dihydro-4*H*-pyrido[3,2-*e*][1,3]thiazin-4-ones and Their Activity Against the Kinetoplastid Parasite *Trypanosoma brucei*

Lee J. Silverberg,[a]* Megan L. Povelones,[b] Madeline F. Malfara,[b] Tapas K. Mal,[c] Carlos N. Pacheco,[c,d] Anthony F. Lagalante,[e] Mark A. Olsen,[e] Hemant P. Yennawar,[f] Hany F. Sobhi,[g] Kayla R. Baney,[a] Robin L. Bozeman,[a] Craig S. Eroh,[a] Michael J. Fleming,[a] Tracy L. Garcia,[a] Casey L. Gregory,[a] Julia E. Hahn,[a] Alyssa M. Hatter,[a] Lexi Johns,[a] Tianna L. Klinger,[a] Jennie Li,[a] Andrew J. Menig,[a] Grace C. Muench,[a] Melissa E. Ramirez,[a] Jordyn Reilly,[a] Nicole Sacco,[a] Alexandra Sheidy,[a] Marla M. Stoner,[a] Eric N. Thompson,[a] Soroush Yazdani[a]

[a] Schuylkill campus, Pennsylvania State University, 200 University Drive, Schuylkill Haven, PA, USA 17972

[b] Department of Biology, Villanova University, Mendel Science Center, 800 Lancaster Avenue, Villanova, PA, USA 19085

[c] Department of Chemistry, Pennsylvania State University, 104 Chemistry Building, Room 08, University Park, PA, USA 16802

[d] Chemistry and Environmental Science, New Jersey Institute of Technology, Tiernan Hall, Room B006, University Heights, Newark, NJ, USA 07102

[e] Department of Chemistry, Villanova University, Mendel Science Center, 800 Lancaster Avenue, Villanova, PA, USA 19085

[f] Department of Biochemistry and Molecular Biology, Pennsylvania State University, 8 Althouse Laboratory, University Park, PA, USA 16802

[g] Center for Organic Synthesis, Coppin State University, Room-229, 2500 West North Avenue, Baltimore, MD, USA 21216

Table of Contents

Content	Page
**Figures S1-S13.** ^{1}H NMR spectra of compounds **1a-i, k-n.**	S3
**Figures S14-S26.** ^{13}C NMR spectra of compounds **1a-i, k-n.**	S16
**Figures S27-S30.** ^{19}F NMR spectra of compounds **1d-e, h-i.**	S29
**Figures S31-S43.** FT-IR spectra of compounds **1a-i, k-n.**	S33
**Figures S44-56.** Mass spectra of compounds **1a-i, k-n.**	S46
**Table S1.** TbBSF 90-13 growth curve data with 50 uM pyridothiazinones **1a**-**1n**.	S59

Figure S1. ^{1}H NMR spectrum of compound **1a** (*p*-NO_2).

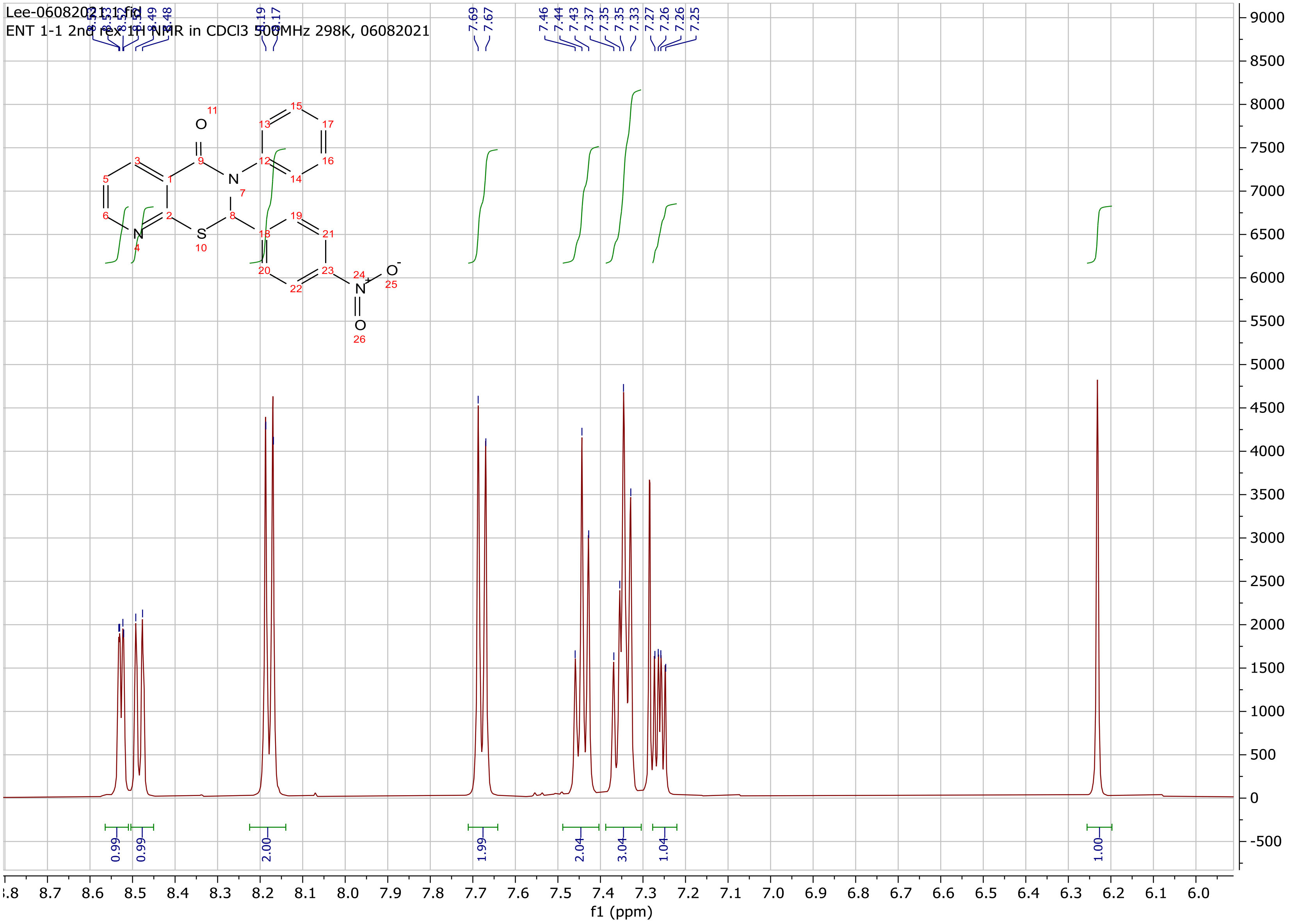

Figure S2. ^{1}H NMR spectrum of compound **1b** (*m*-NO_2).

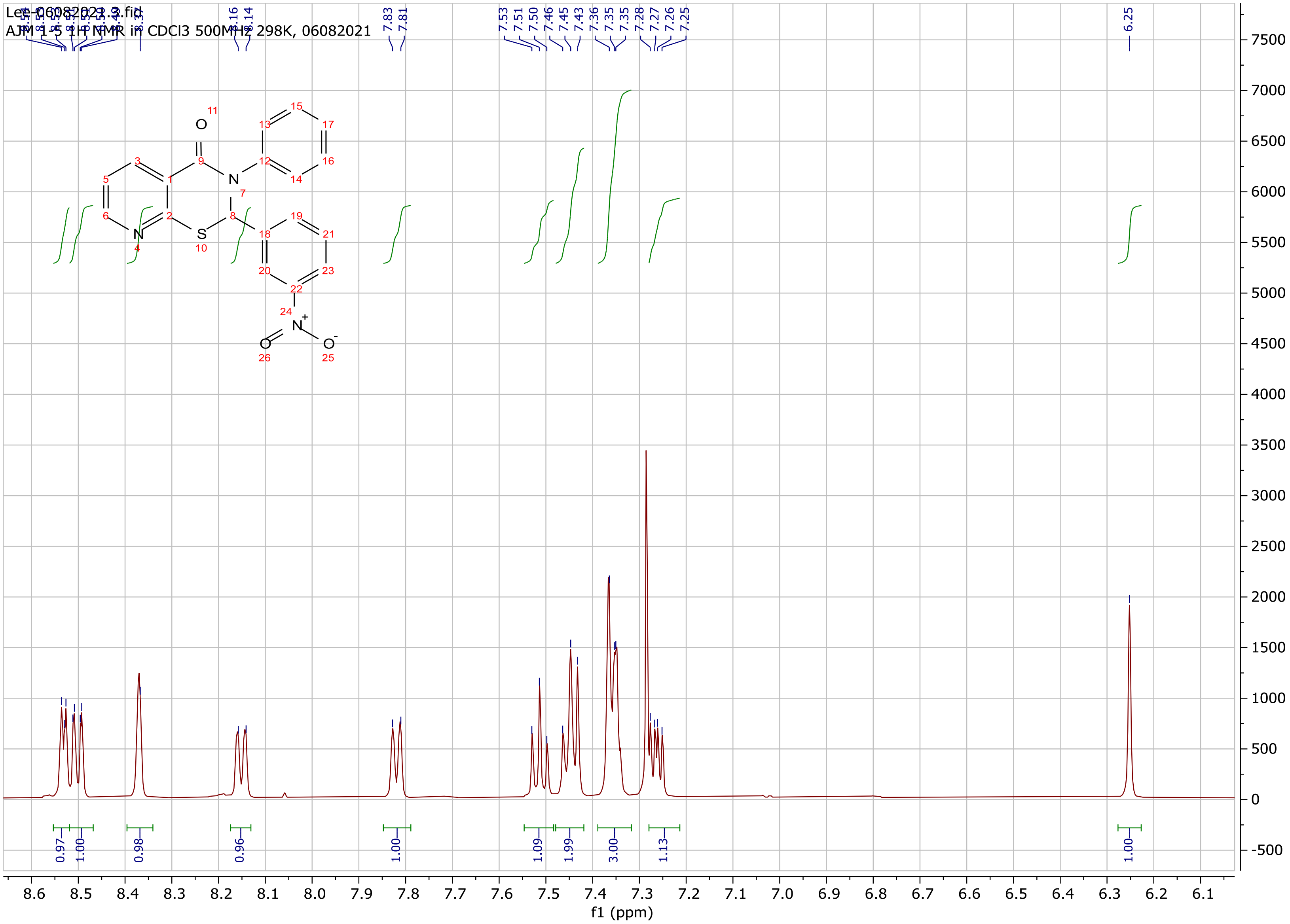

Figure S3. ^{1}H NMR spectrum of compound **1c** (*o*-NO_2).

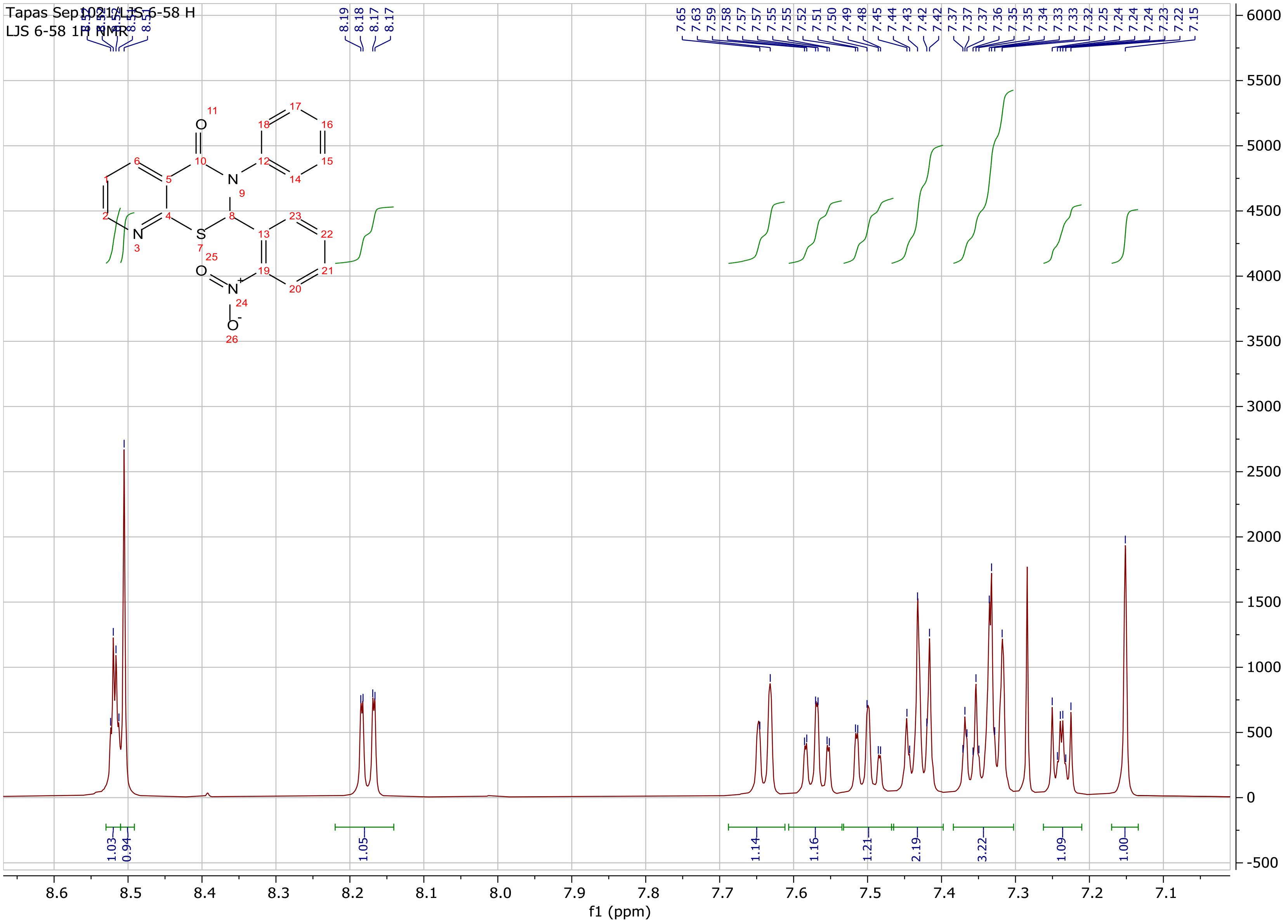

Figure S4. ^{1}H NMR spectrum of compound **1d** (p-CF_3).

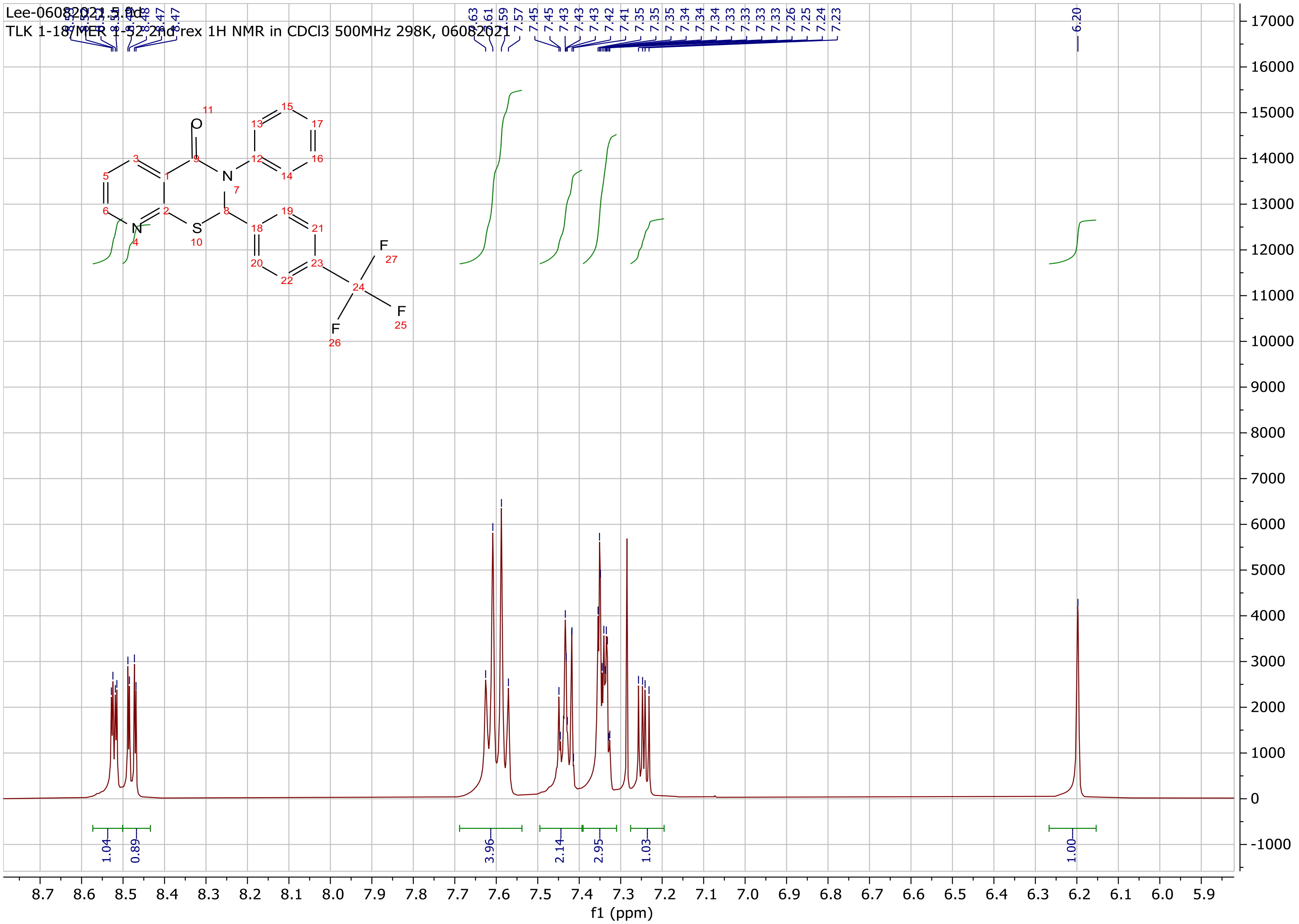

Figure S5. ^{1}H NMR spectrum of compound **1e** (*m*-CF_3).

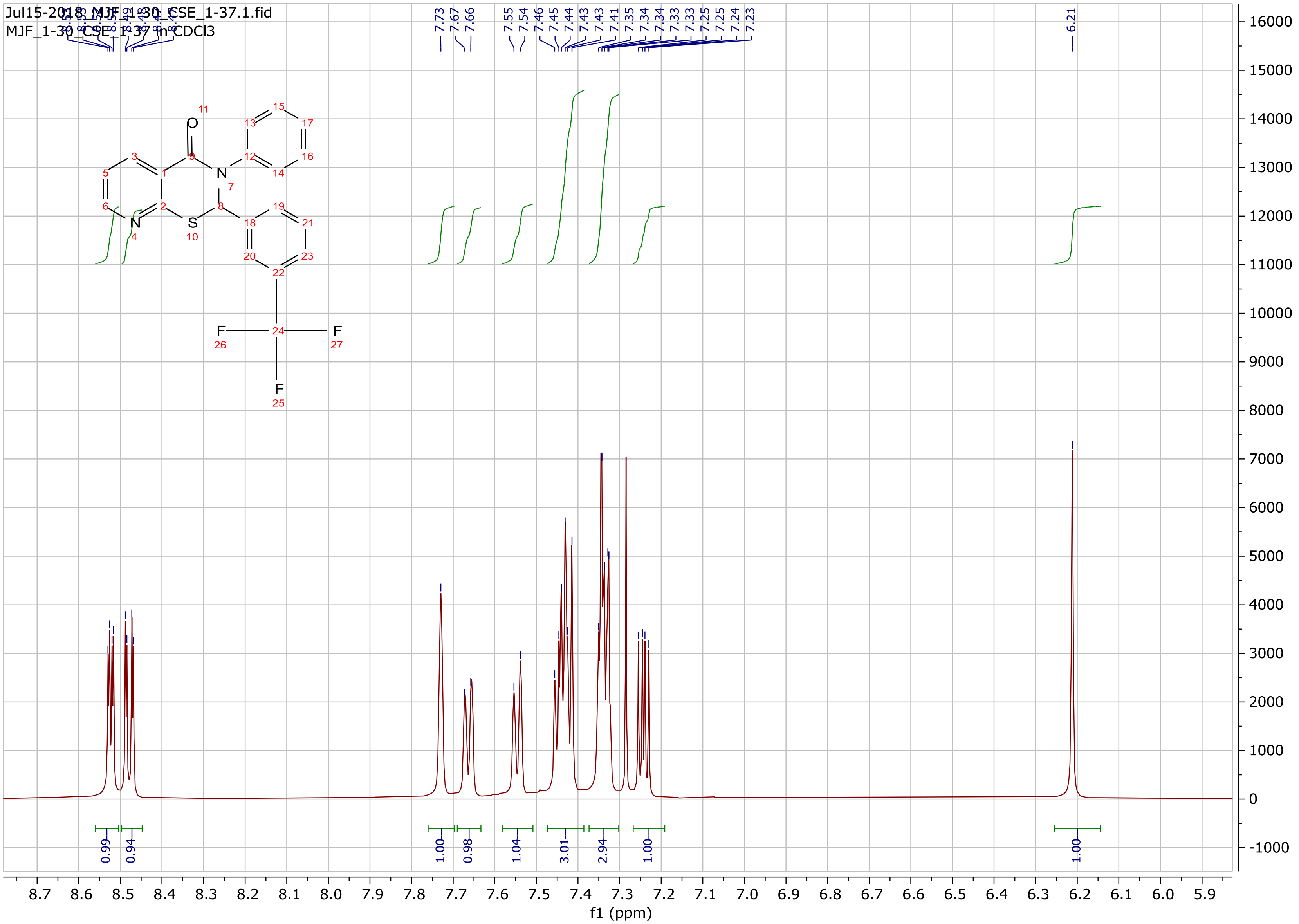

Figure S6. ^{1}H NMR spectrum of compound **1f** (*p*-Br).

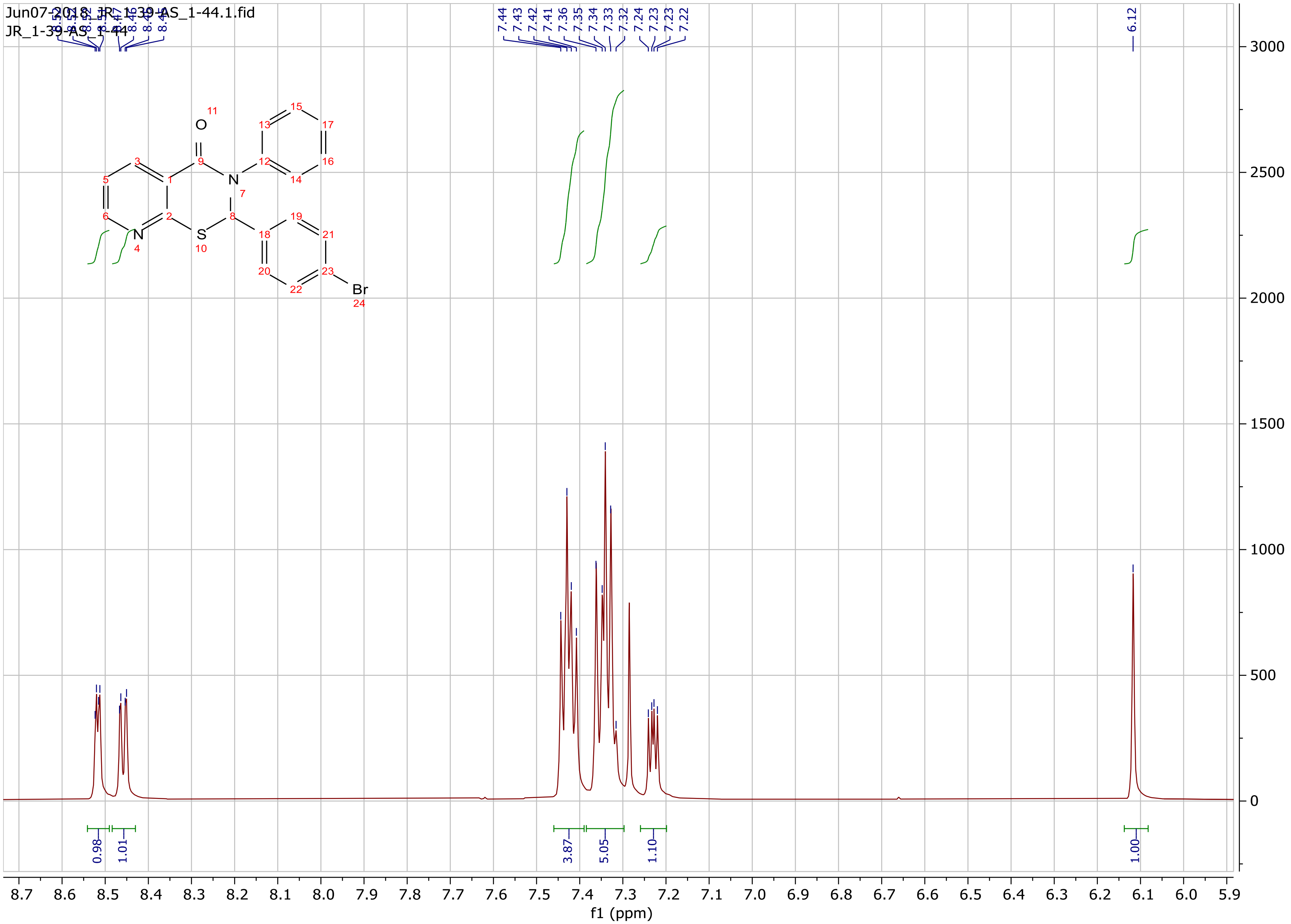

Figure S7. ^{1}H NMR spectrum of compound **1g** (*m*-Br).

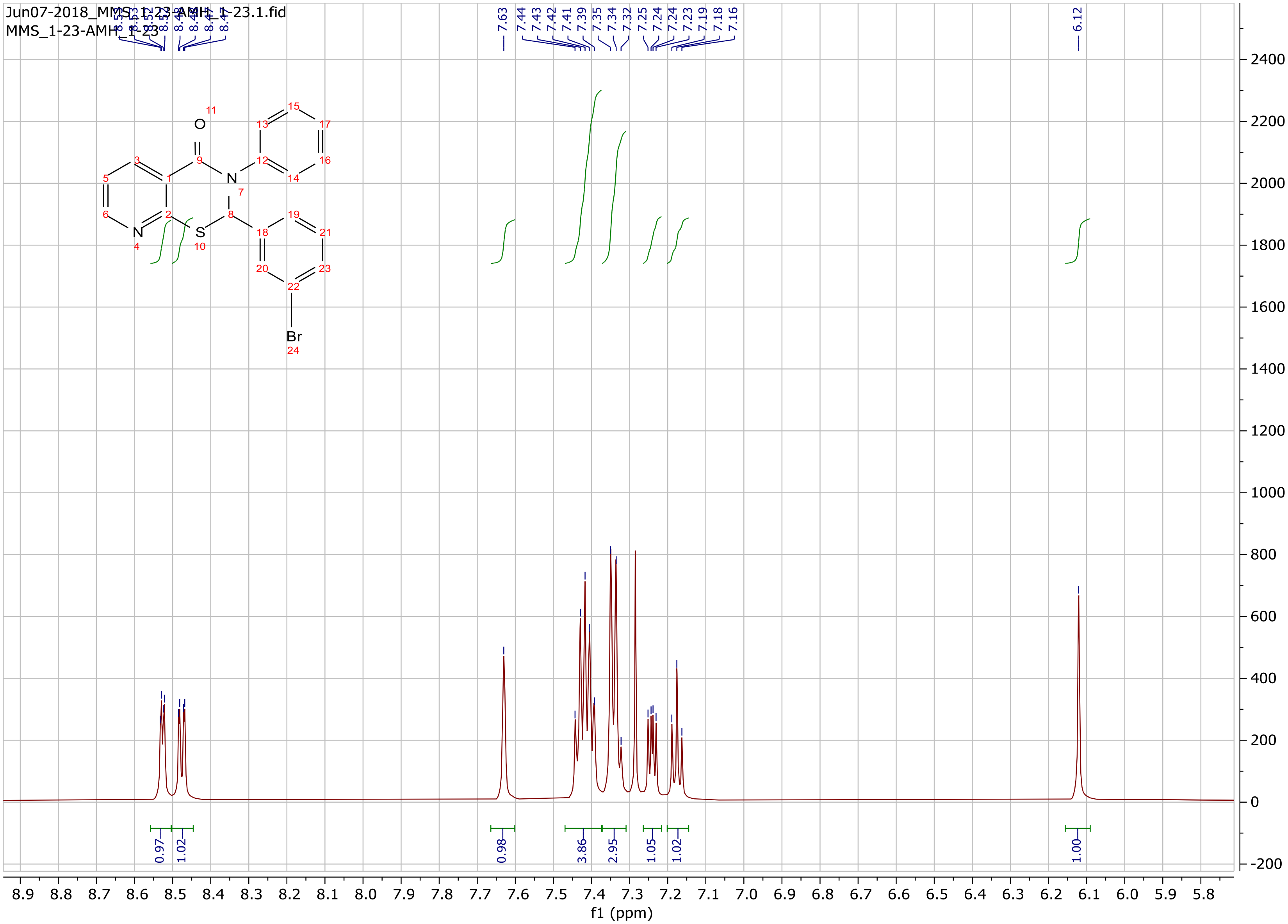

Figure S8. ^{1}H NMR spectrum of compound **1h** (*p*-F).

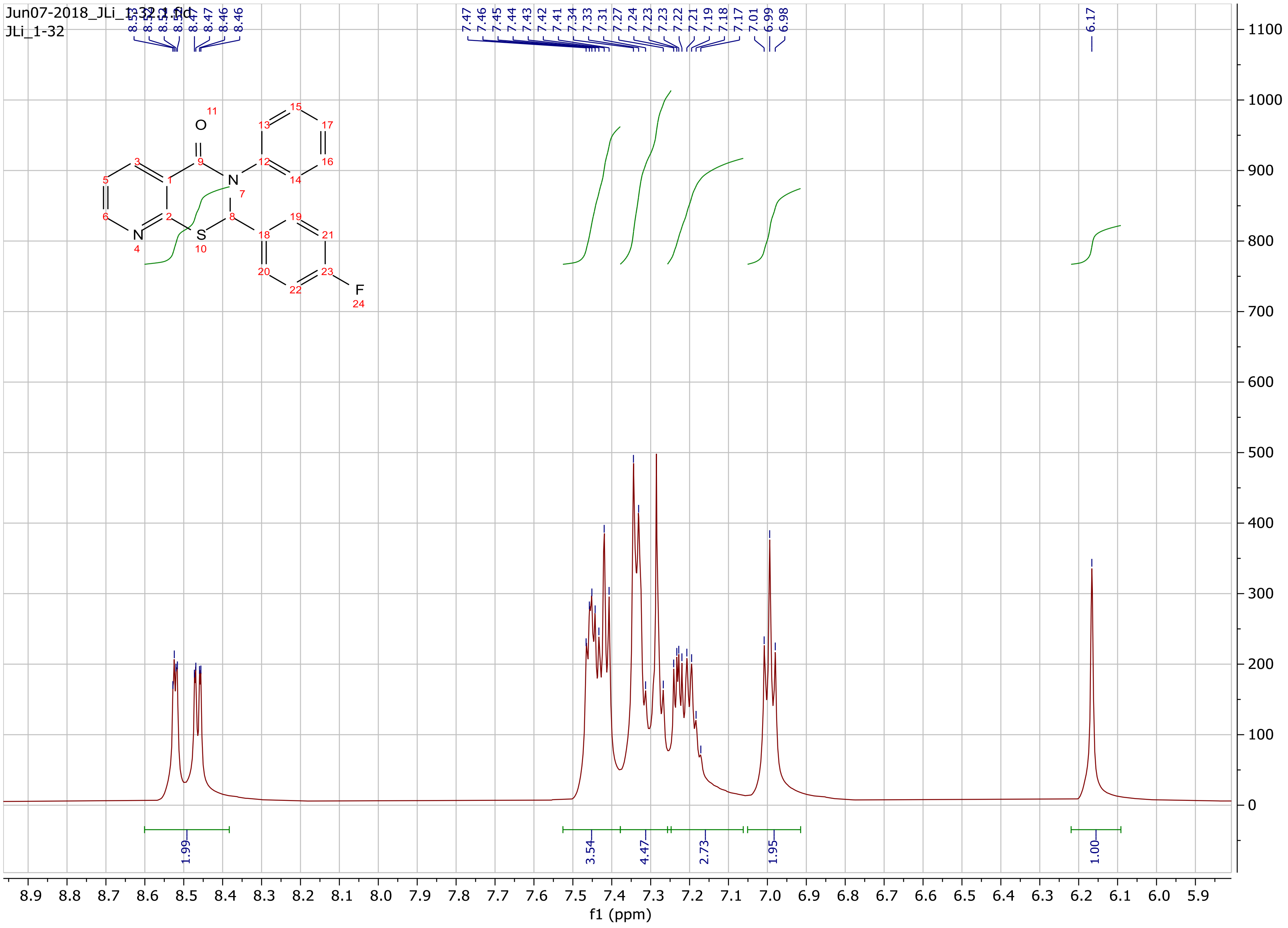

Figure S9. ^{1}H NMR spectrum of compound **1i** (*m*-F).

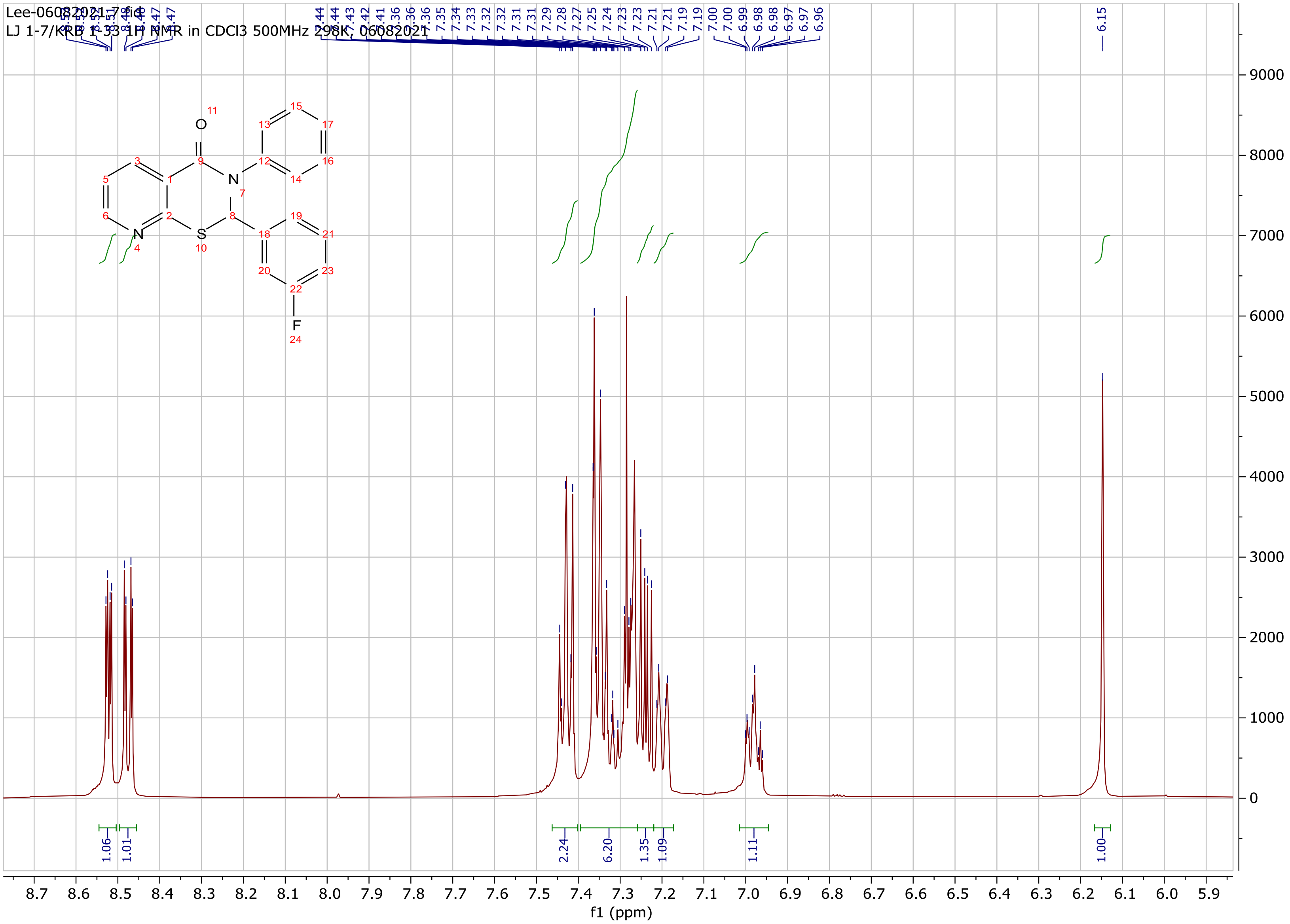

Figure S10. ^{1}H NMR spectrum of compound **1k** (*p*-Me).

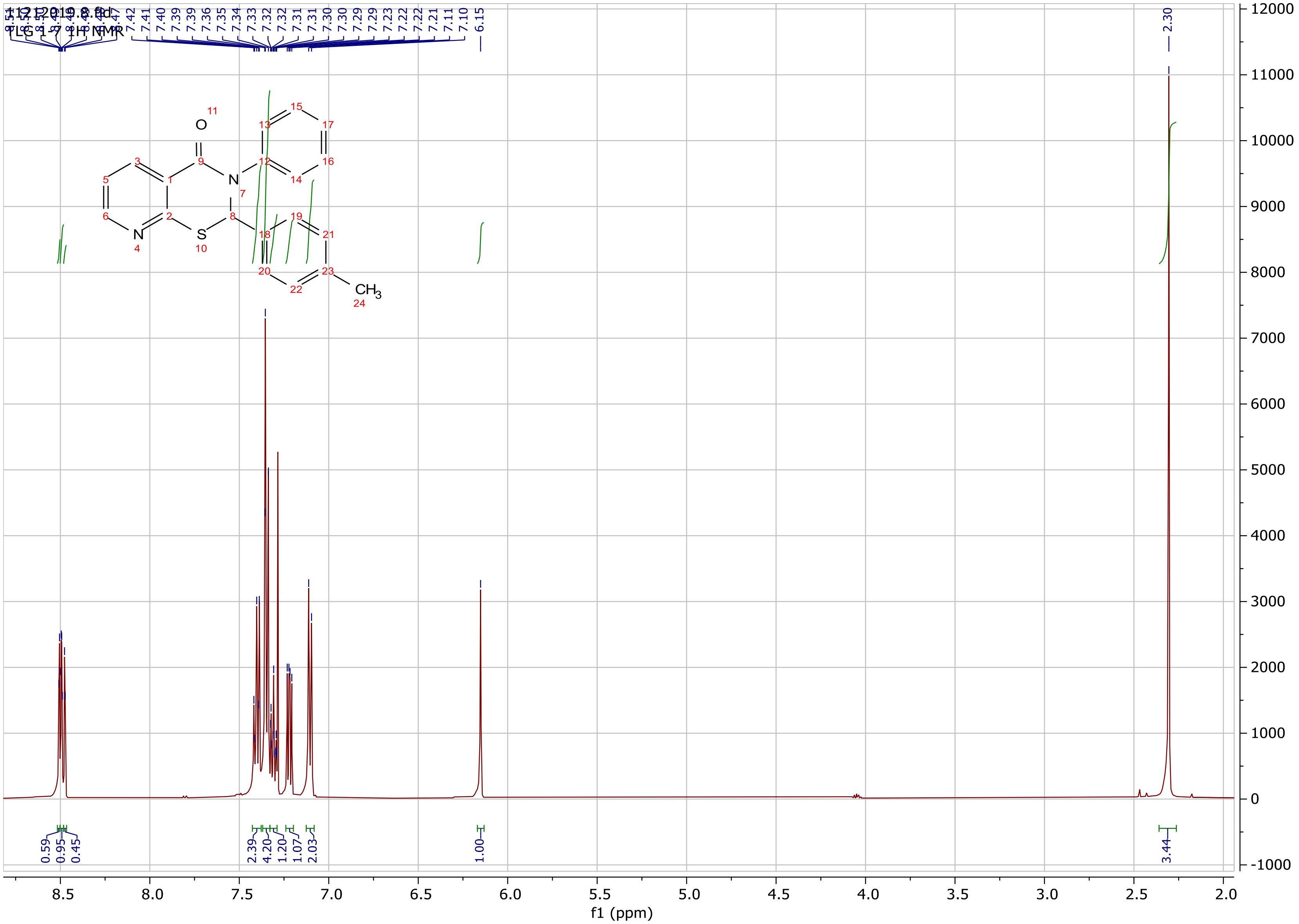

Figure S11. ^{1}H NMR spectrum of compound **1l** (*m*-Me).

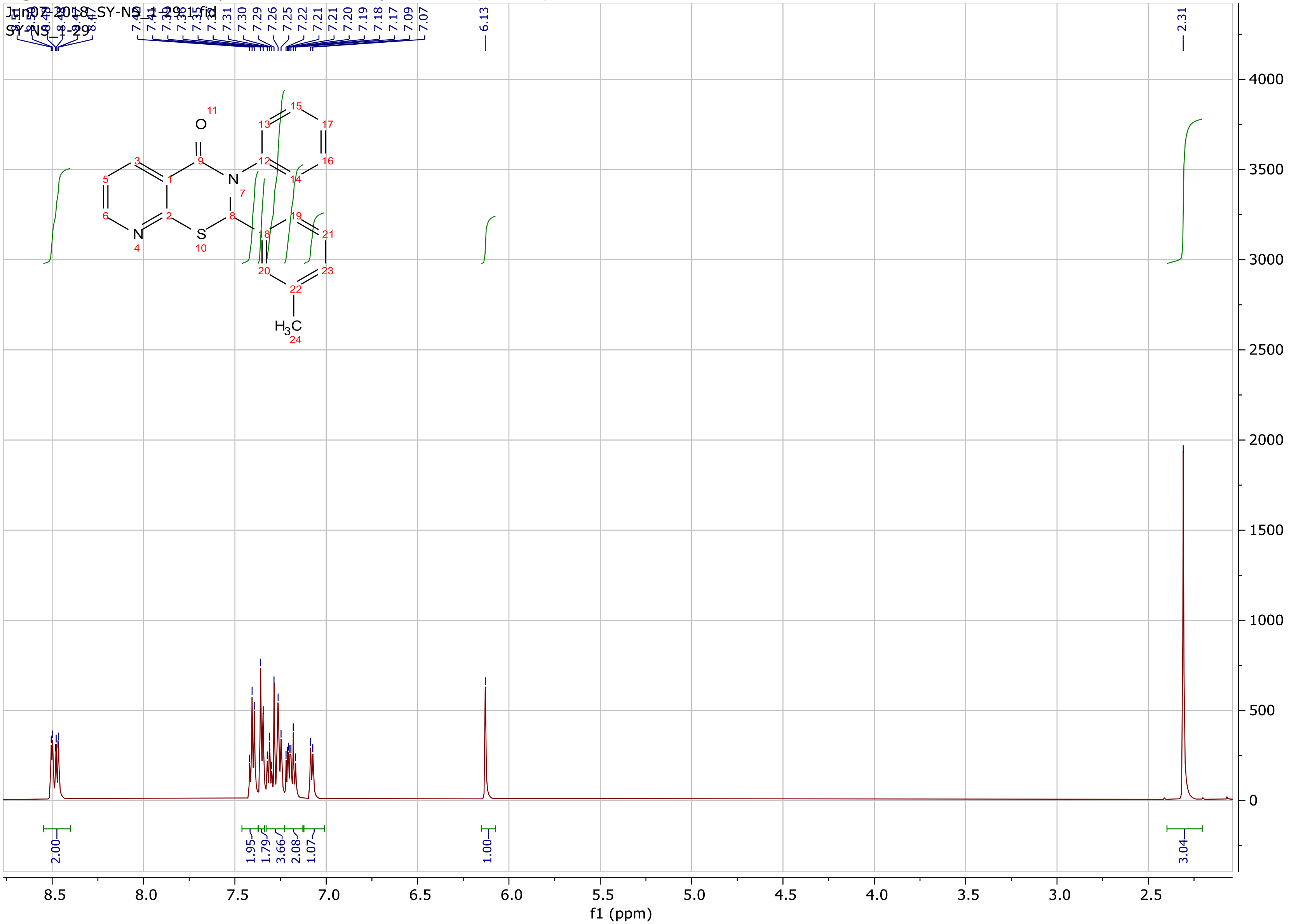

Figure S12. ^{1}H NMR spectrum of compound **1m** (*p*-OMe).

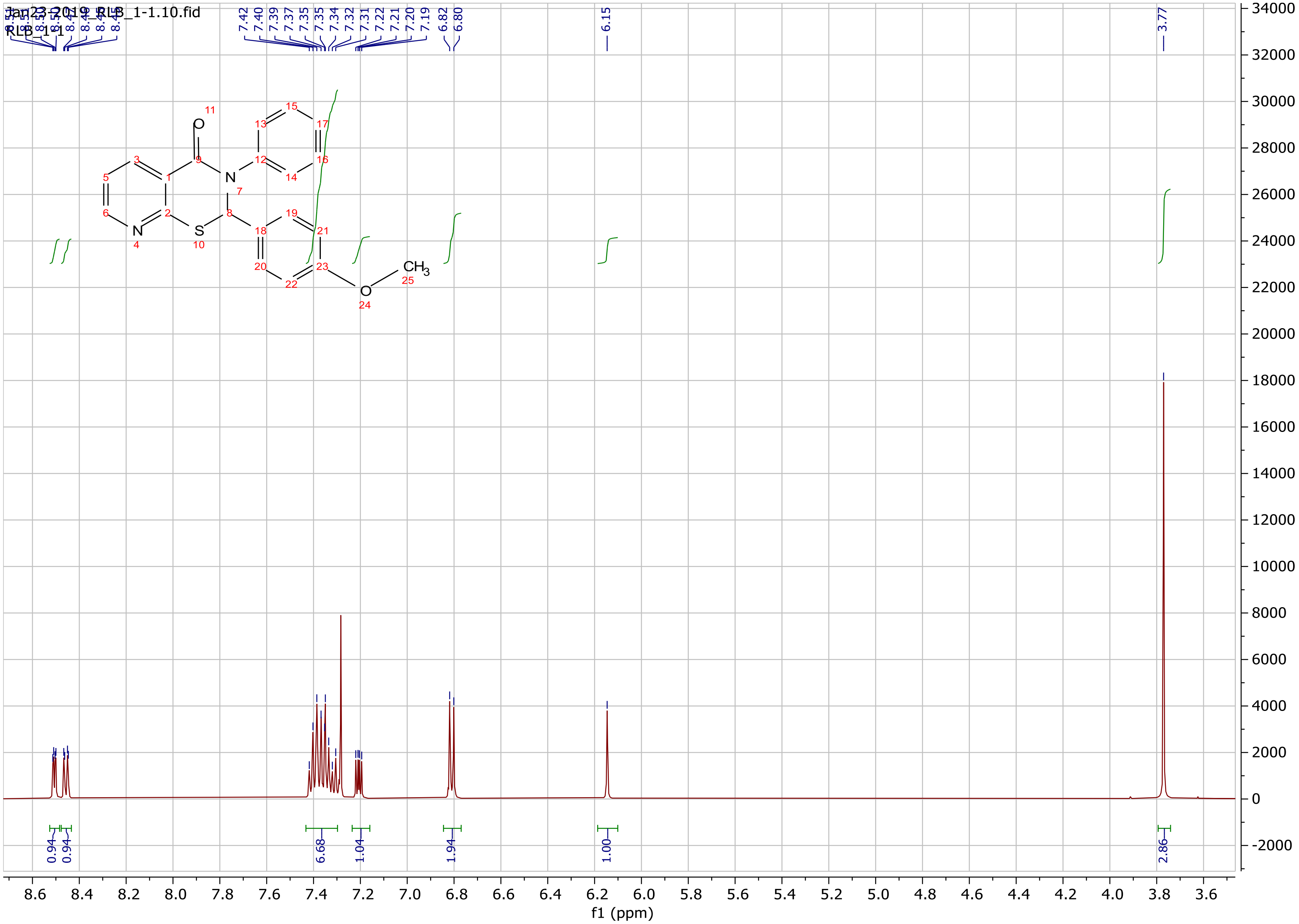

Figure S13. ^{1}H NMR spectrum of compound **1n** (*m*-OMe).

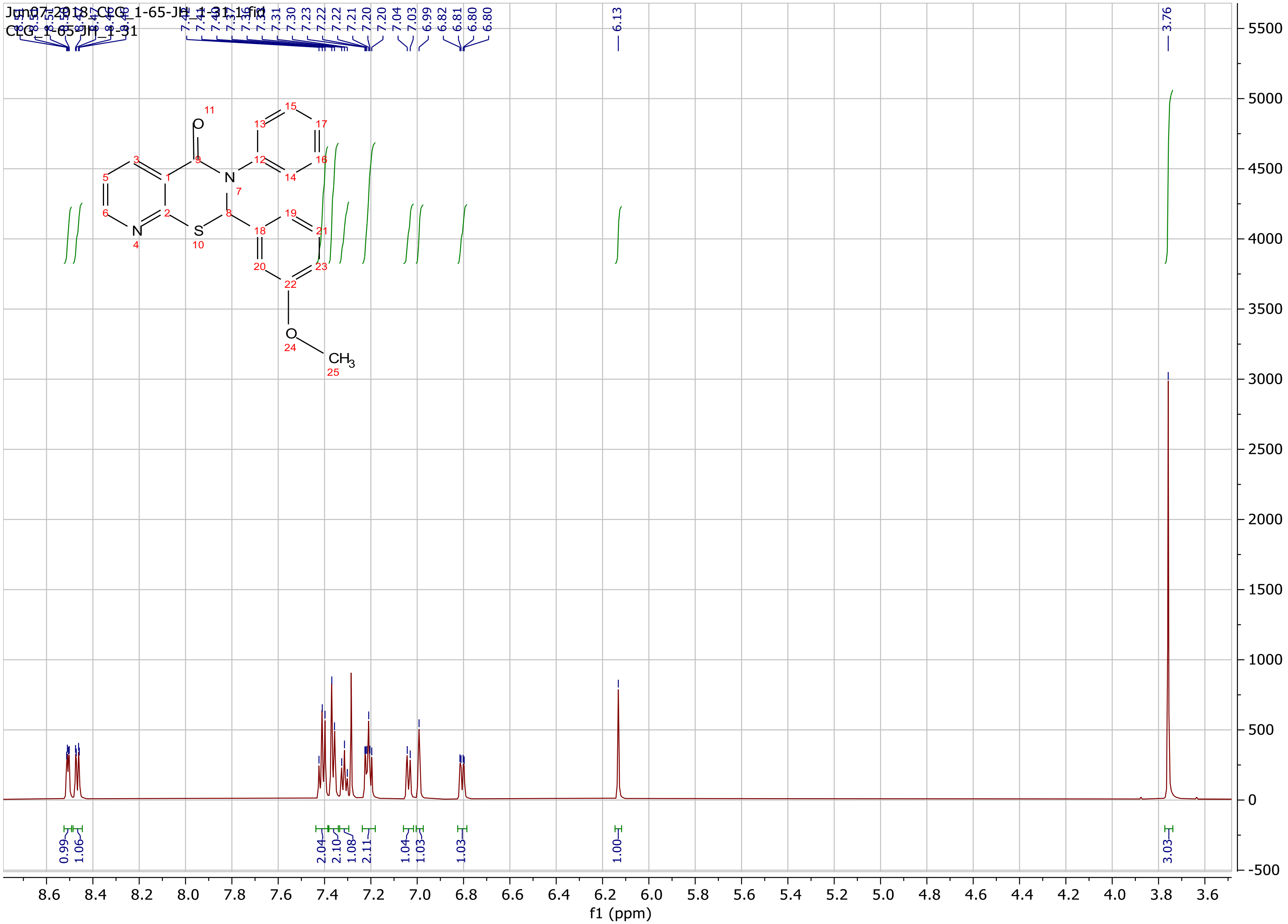

Figure S14. ^{13}C NMR spectrum of compound **1a** (*p*-NO_2).

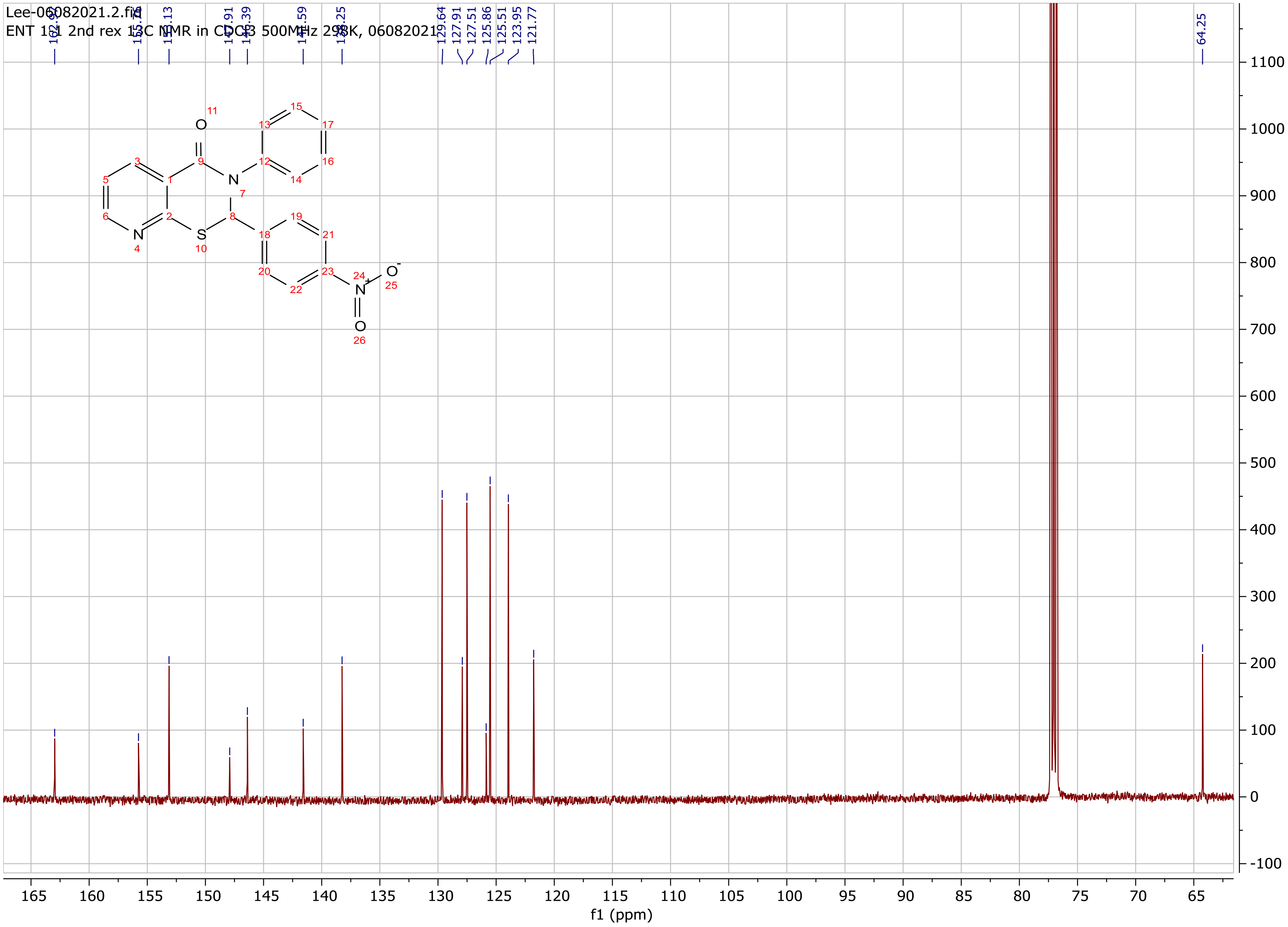

Figure S15. ^{13}C NMR spectrum of compound **1b** (*m*-NO_2).

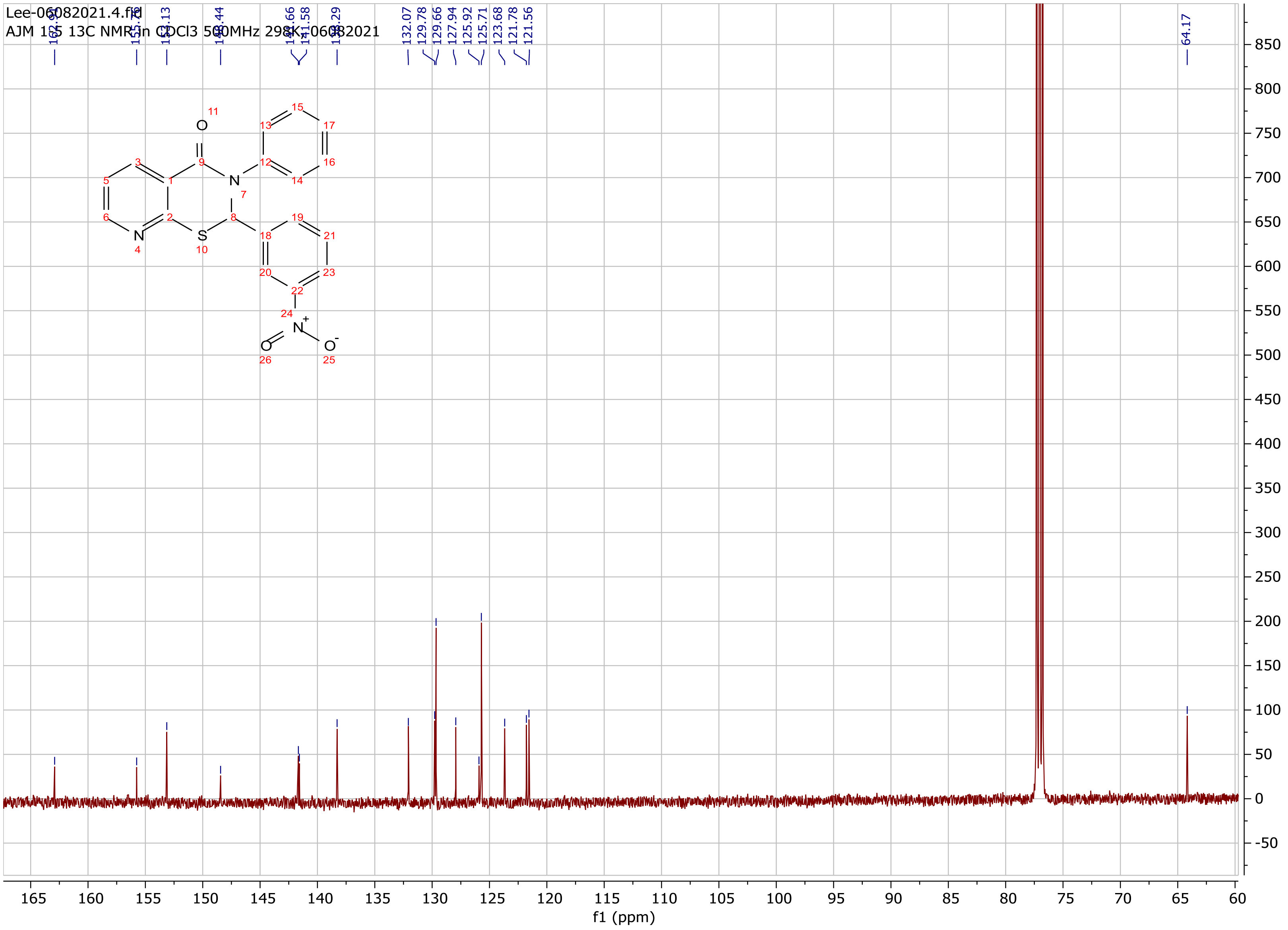

Figure S16. ^{13}C NMR spectrum of compound **1c** (*o*-NO_2).

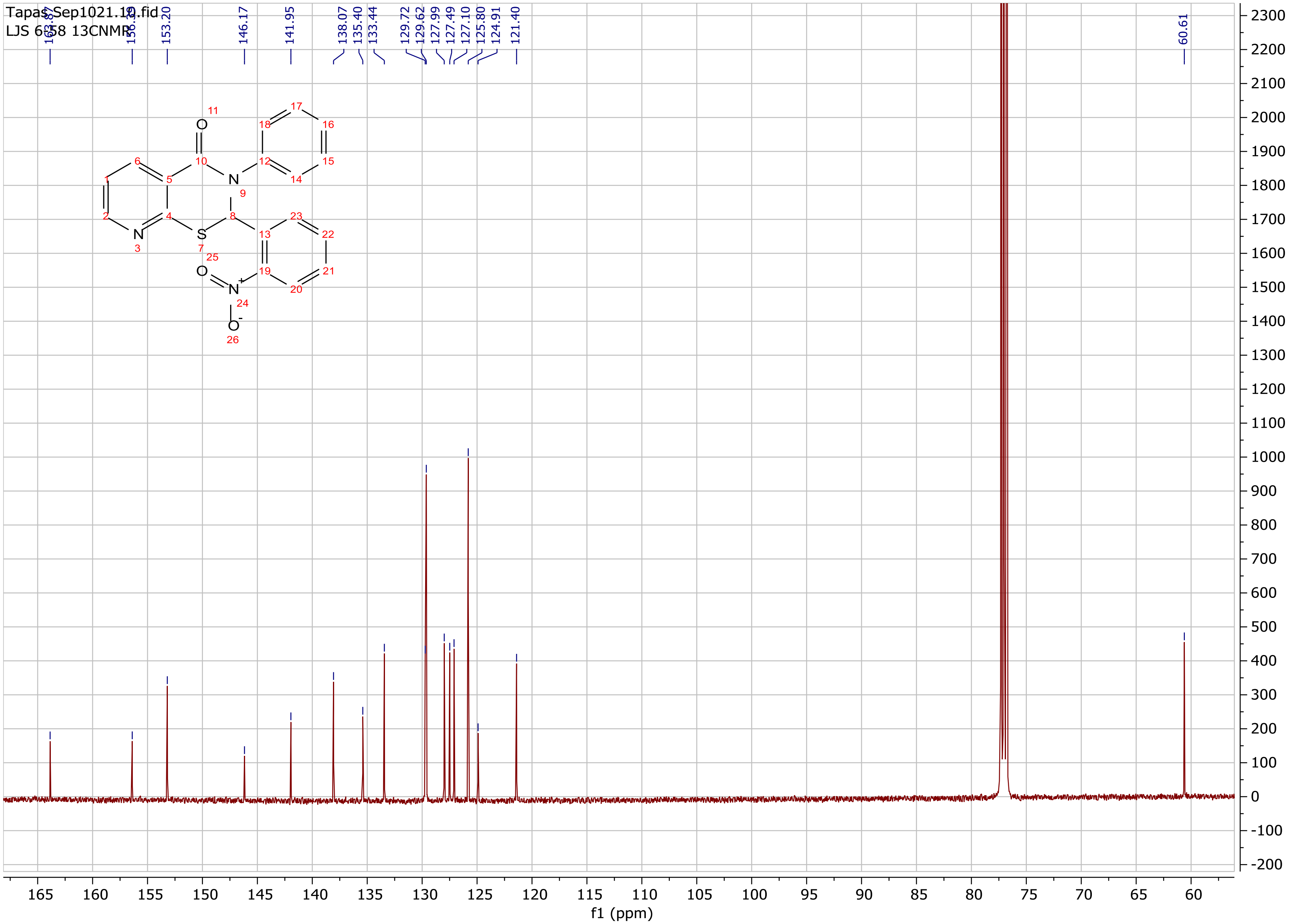

Figure S17. ^{13}C NMR spectrum of compound **1d** (p-CF_3).

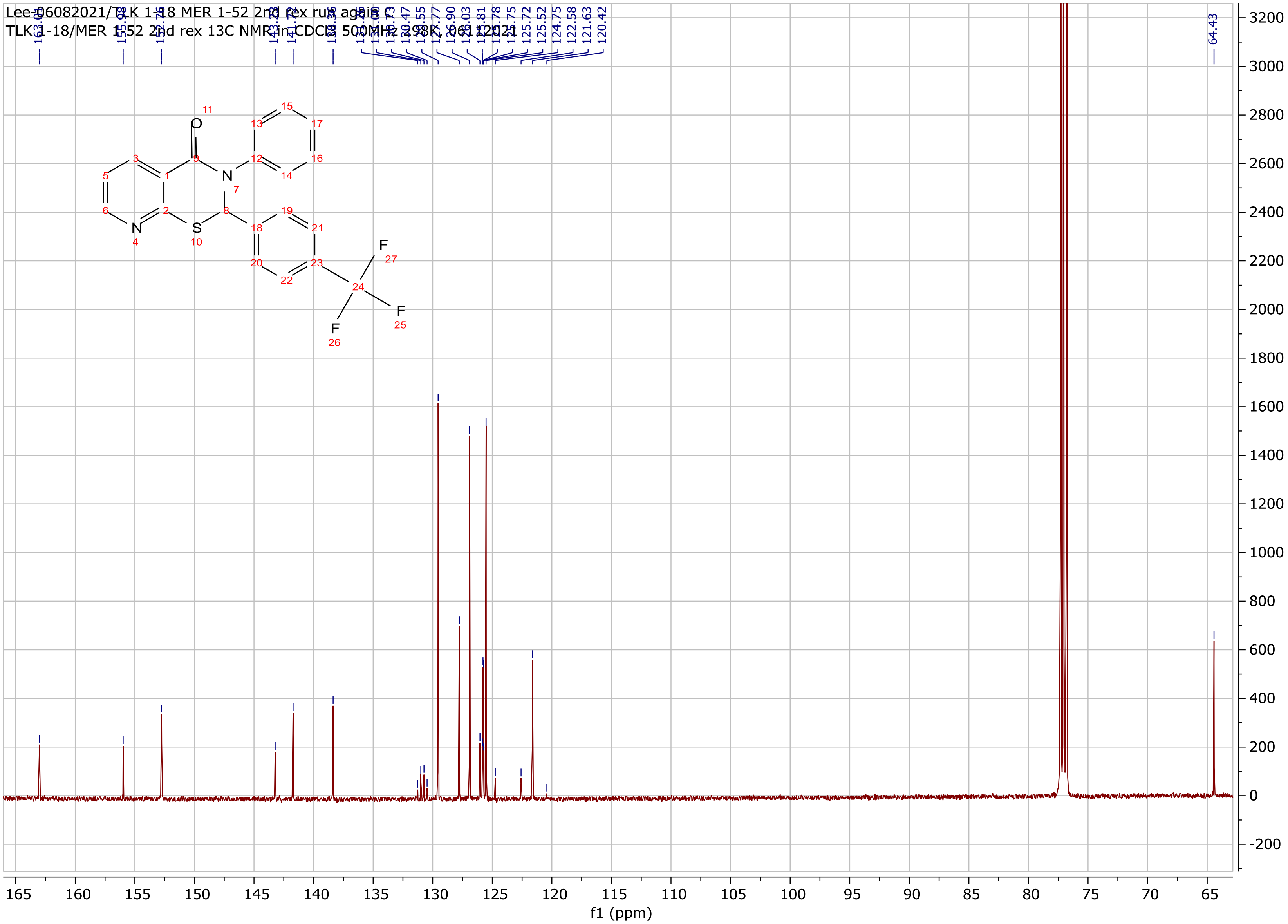

Figure S18. ^{13}C NMR spectrum of compound **1e** (*m*-CF_3).

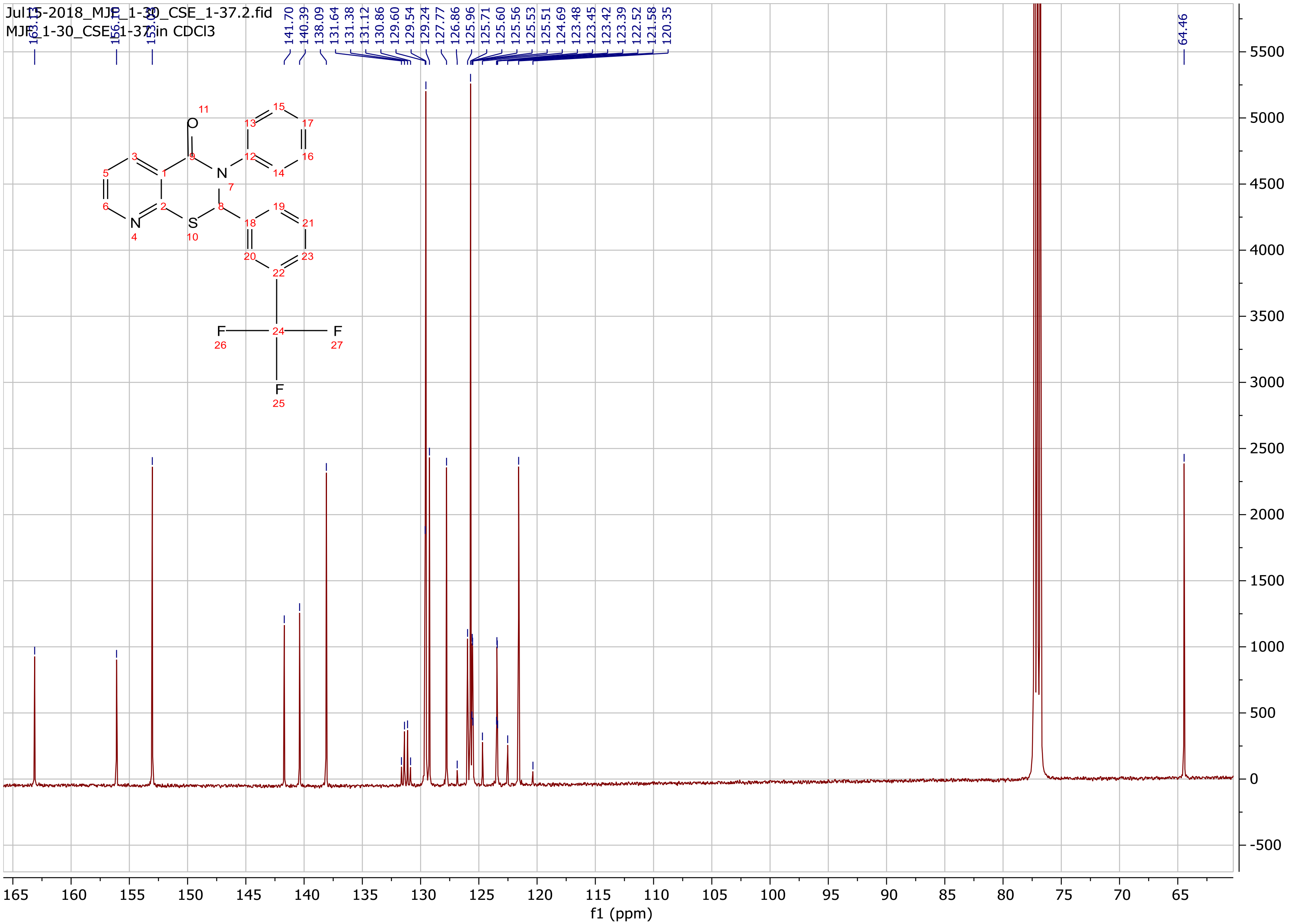

Figure S19. ^{13}C NMR spectrum of compound **1f** (*p*-Br).

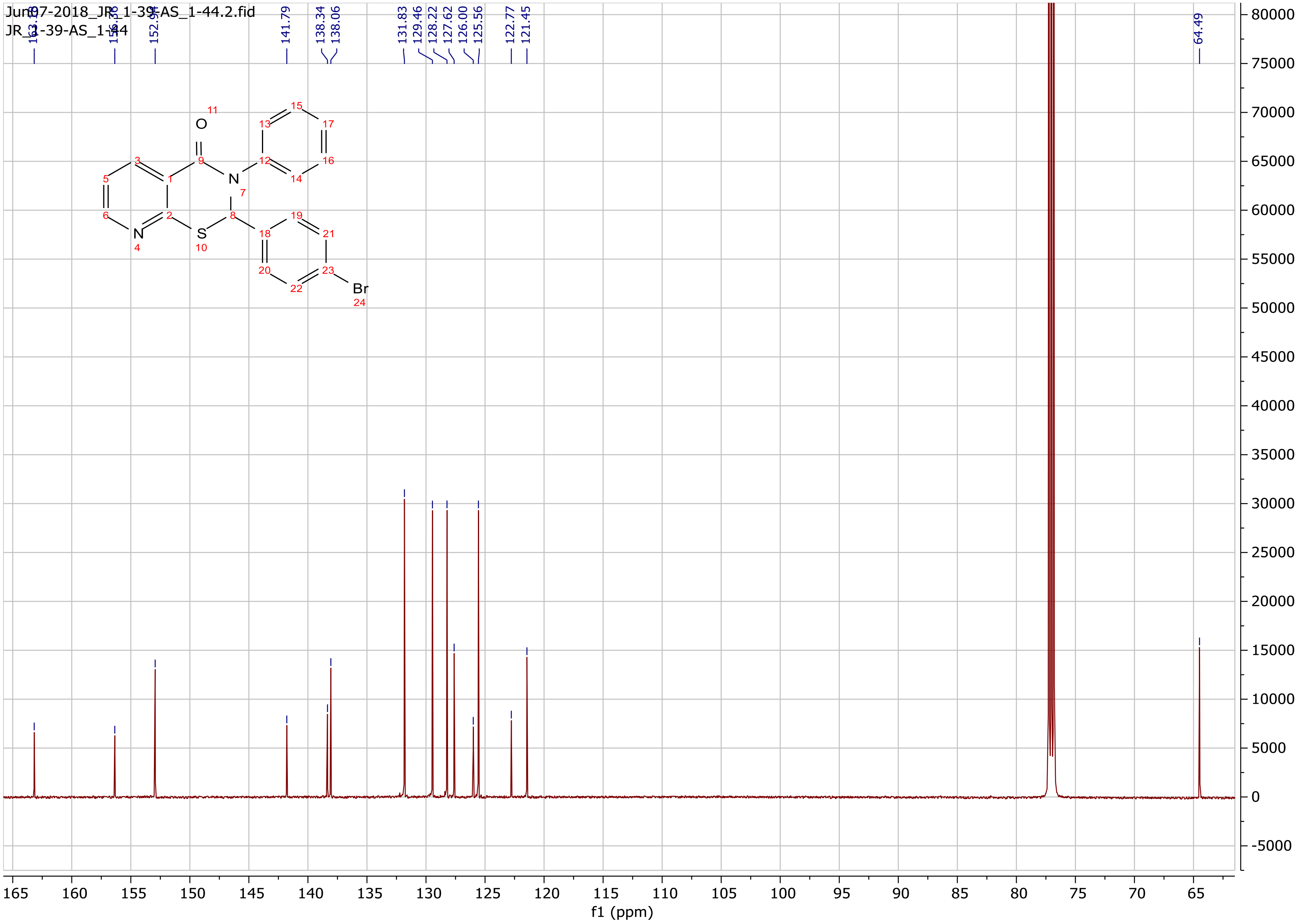

Figure S20. ^{13}C NMR spectrum of compound **1g** (*m*-Br).

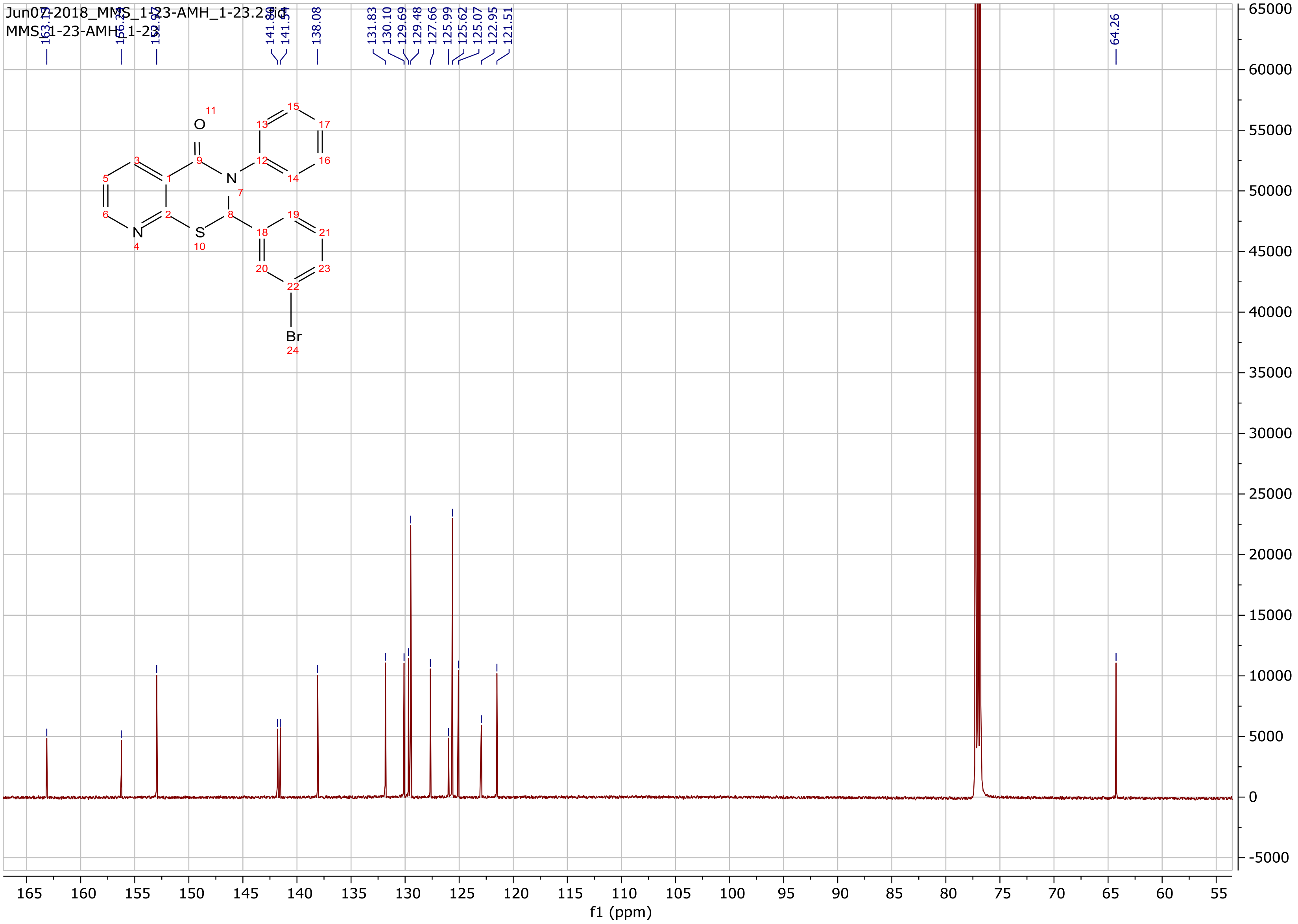

Figure S21. ^{13}C NMR spectrum of compound **1h** (*p*-F).

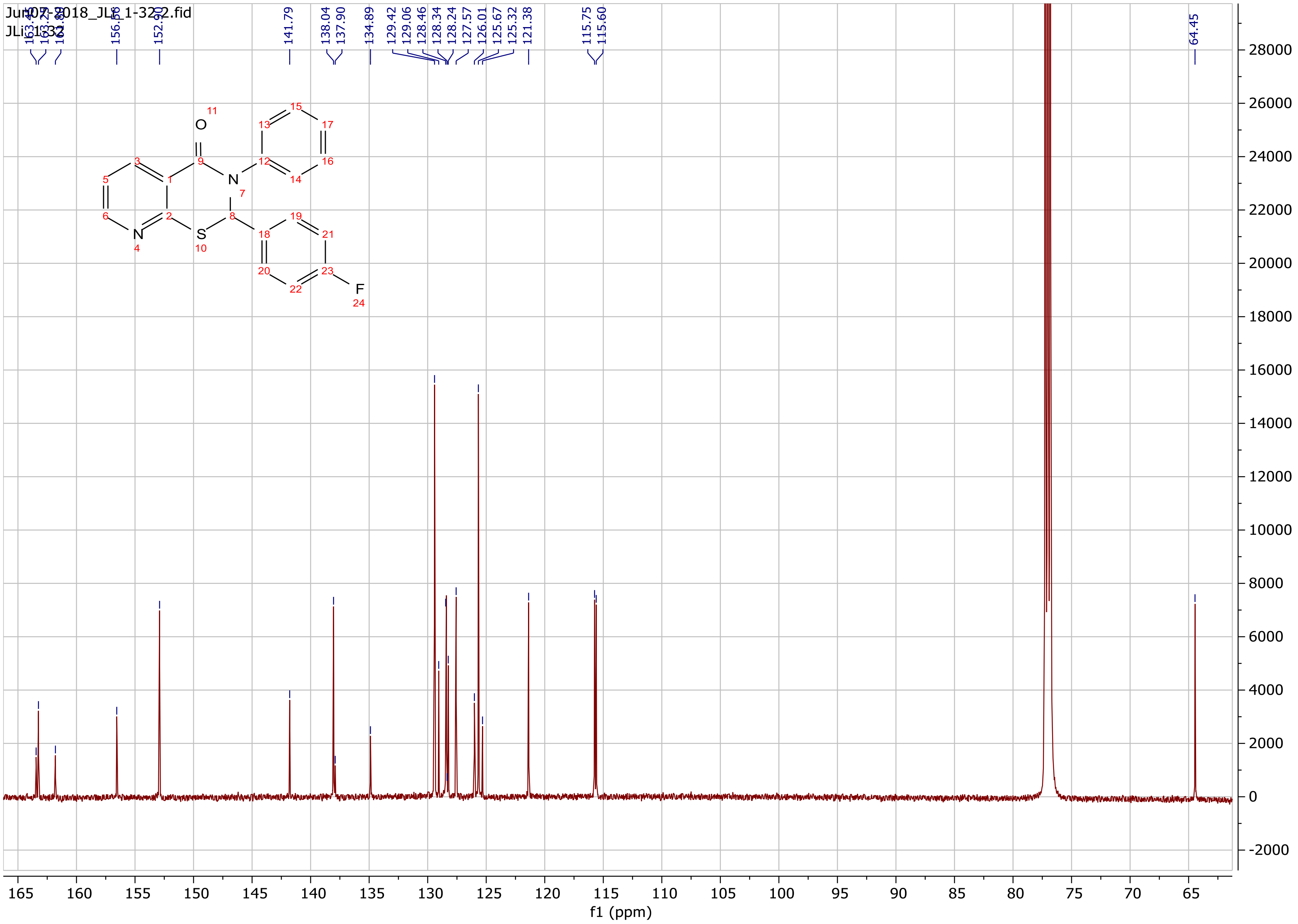

Figure S22. ^{13}C NMR spectrum of compound **1i** (*m*-F).

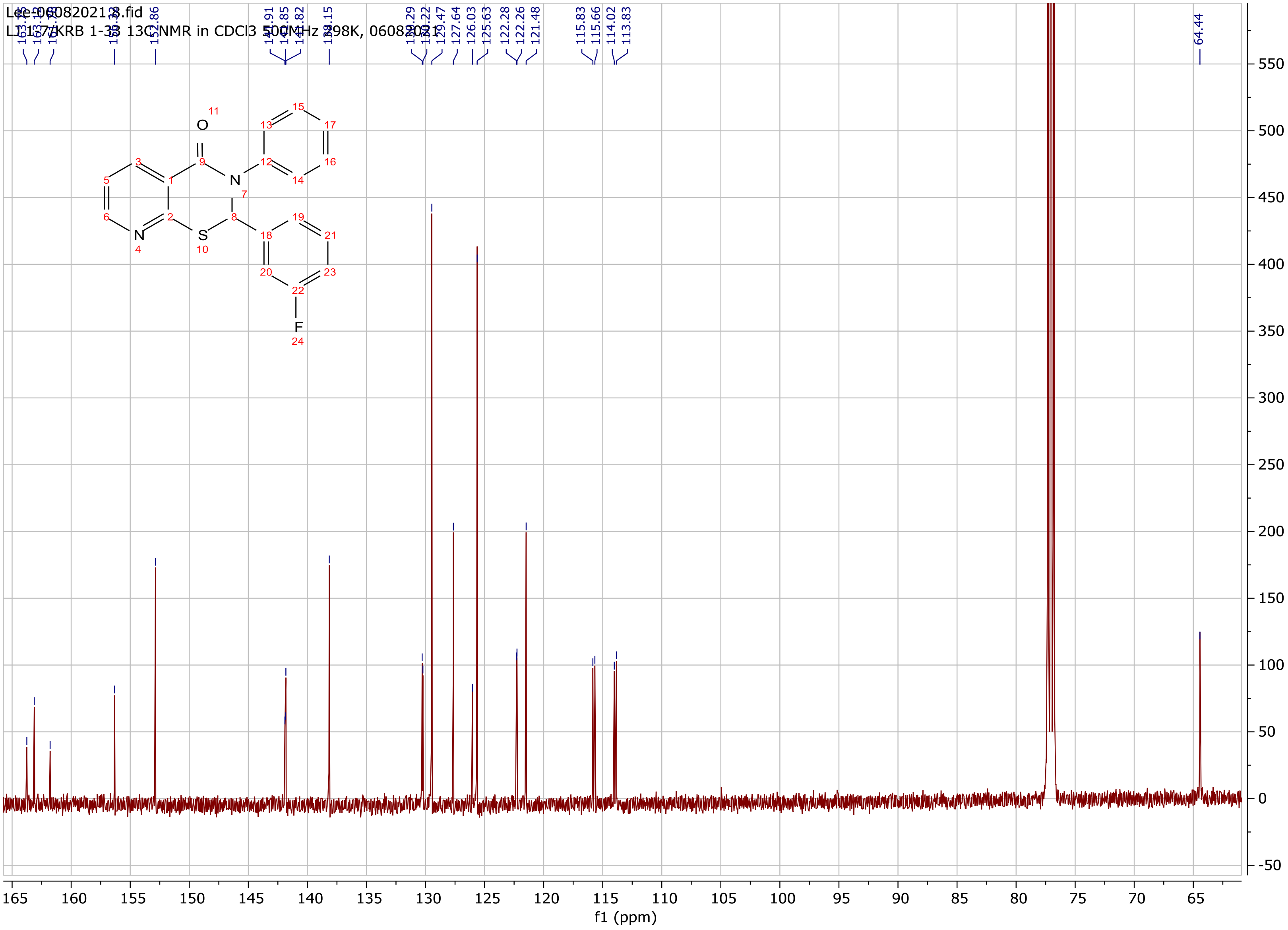

Figure S23. ^{13}C NMR spectrum of compound **1k** (*p*-Me).

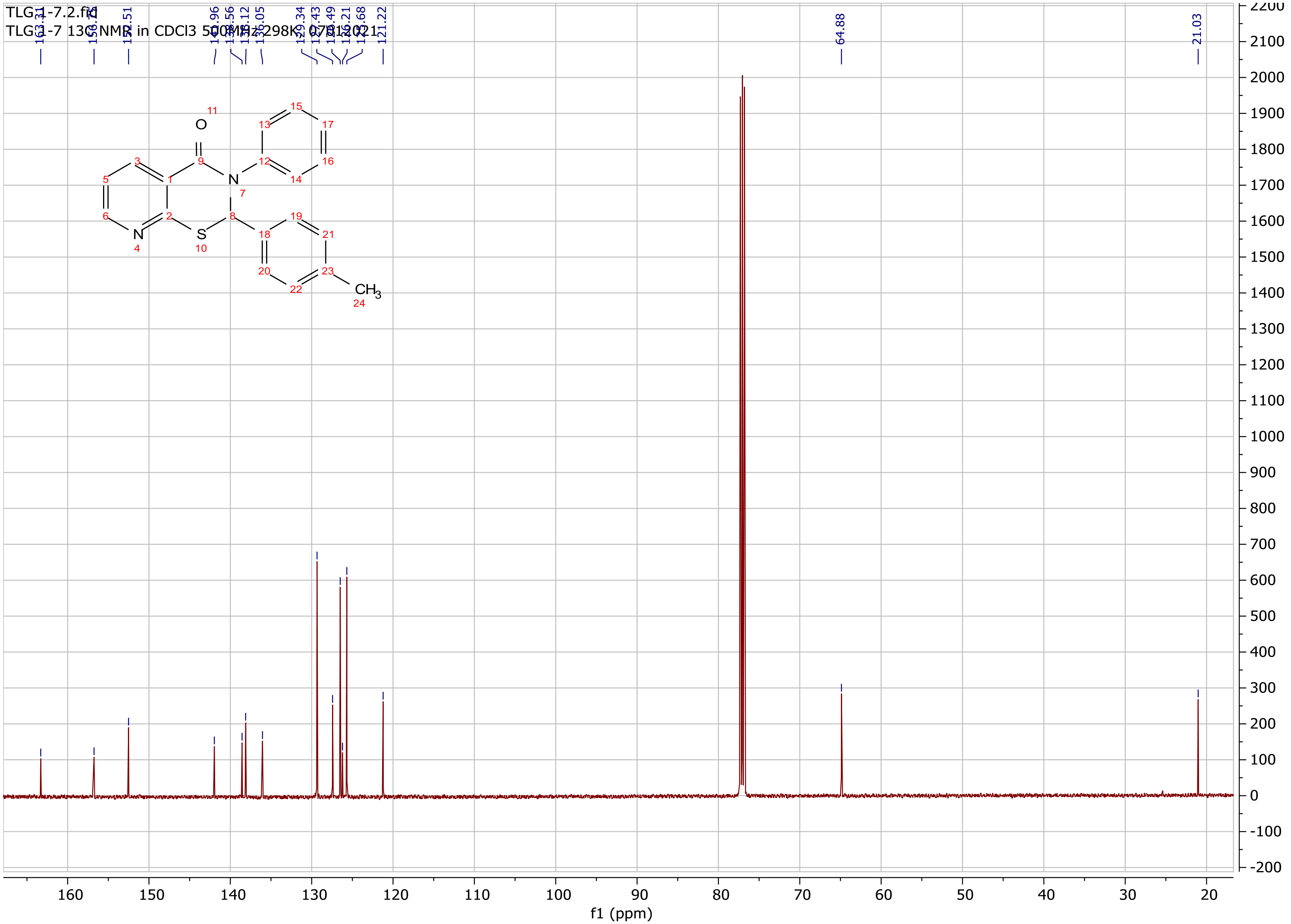

Figure S24. ^{13}C NMR spectrum of compound **1l** (*m*-Me).

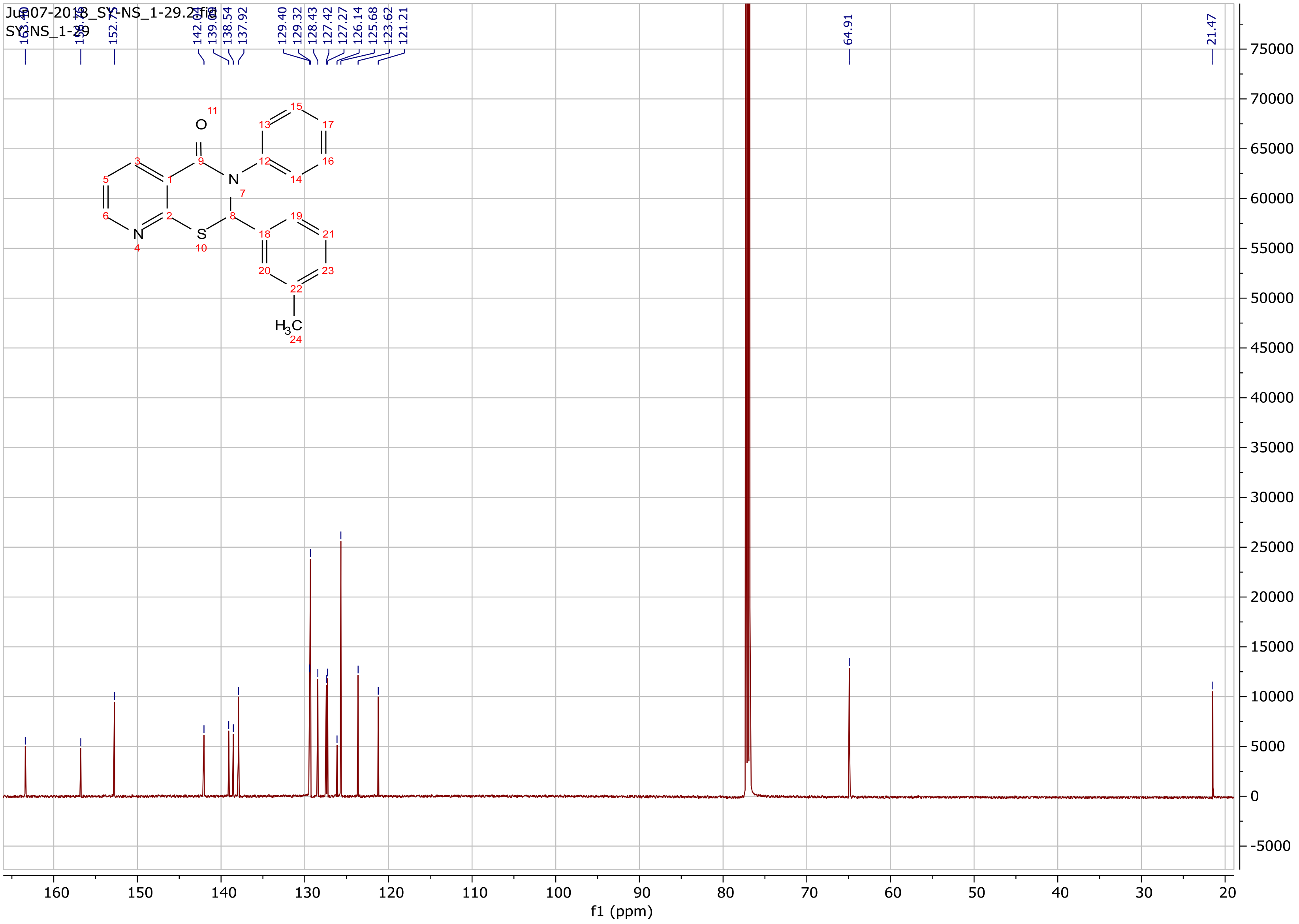

Figure S25. ^{13}C NMR spectrum of compound **1m** (*p*-OMe).

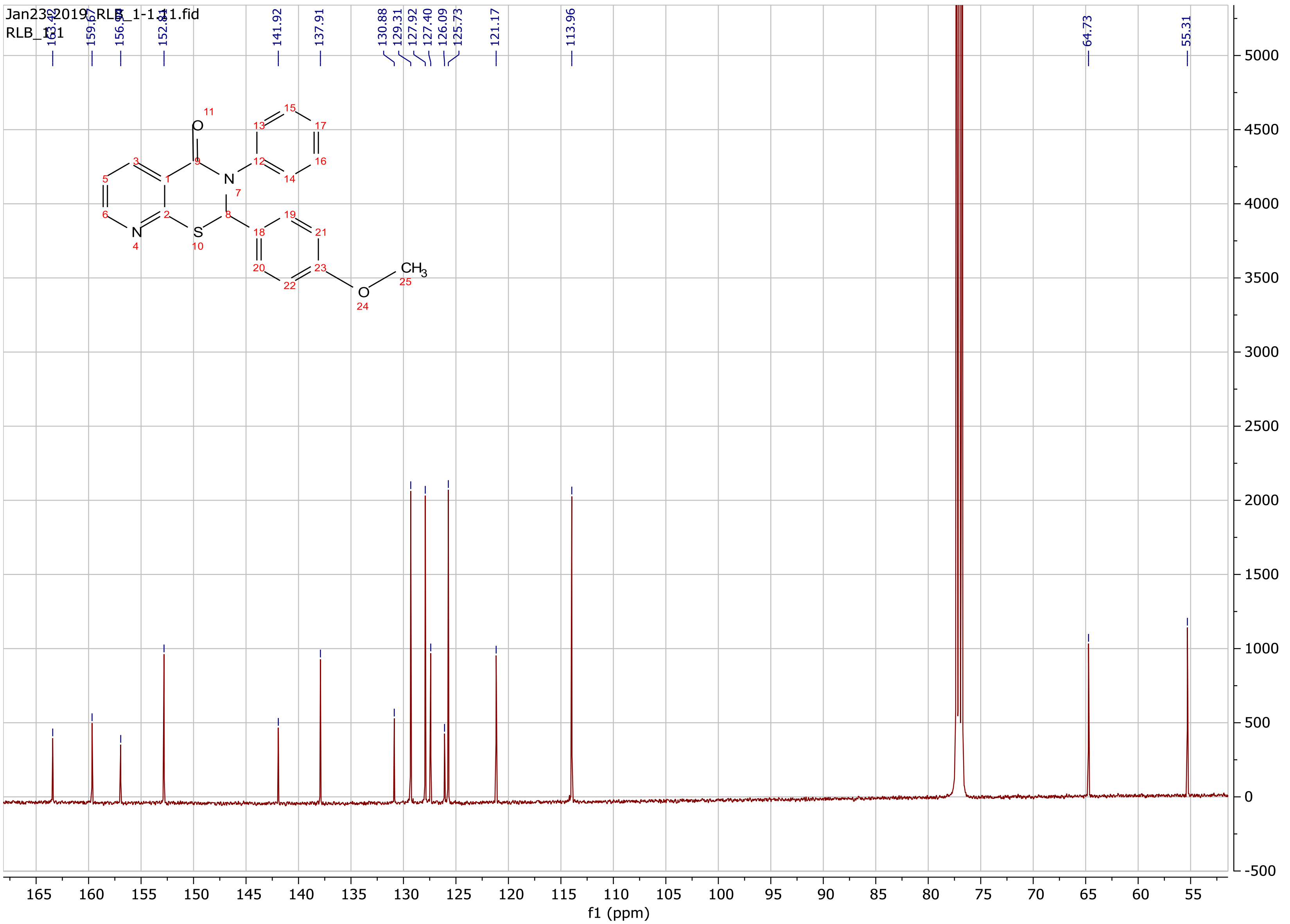

Figure S26. ^{13}C NMR spectrum of compound **1n** (*m*-OMe).

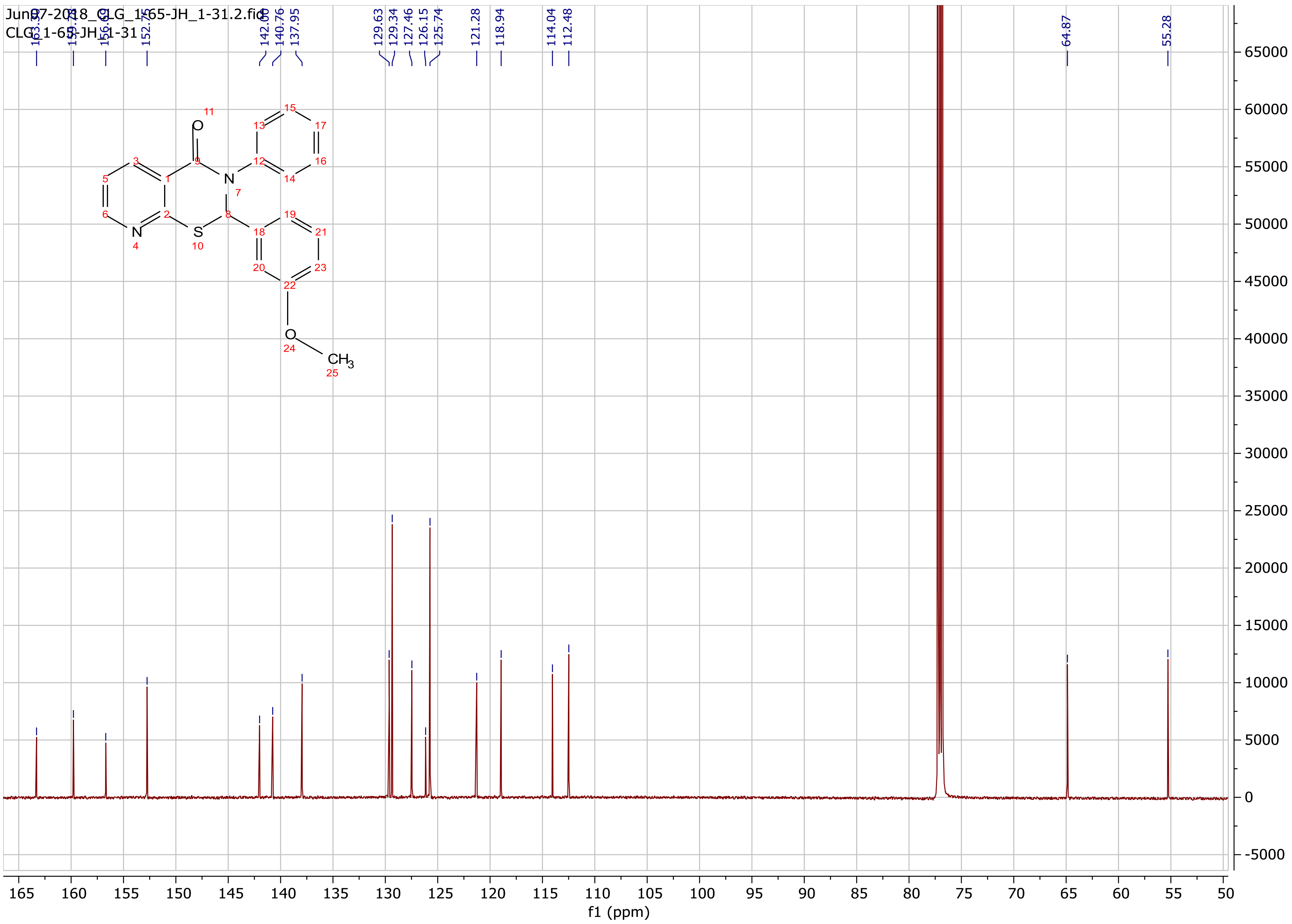

Figure S27. ^{19}F NMR spectrum of compound **1d** (*p*-CF_3).

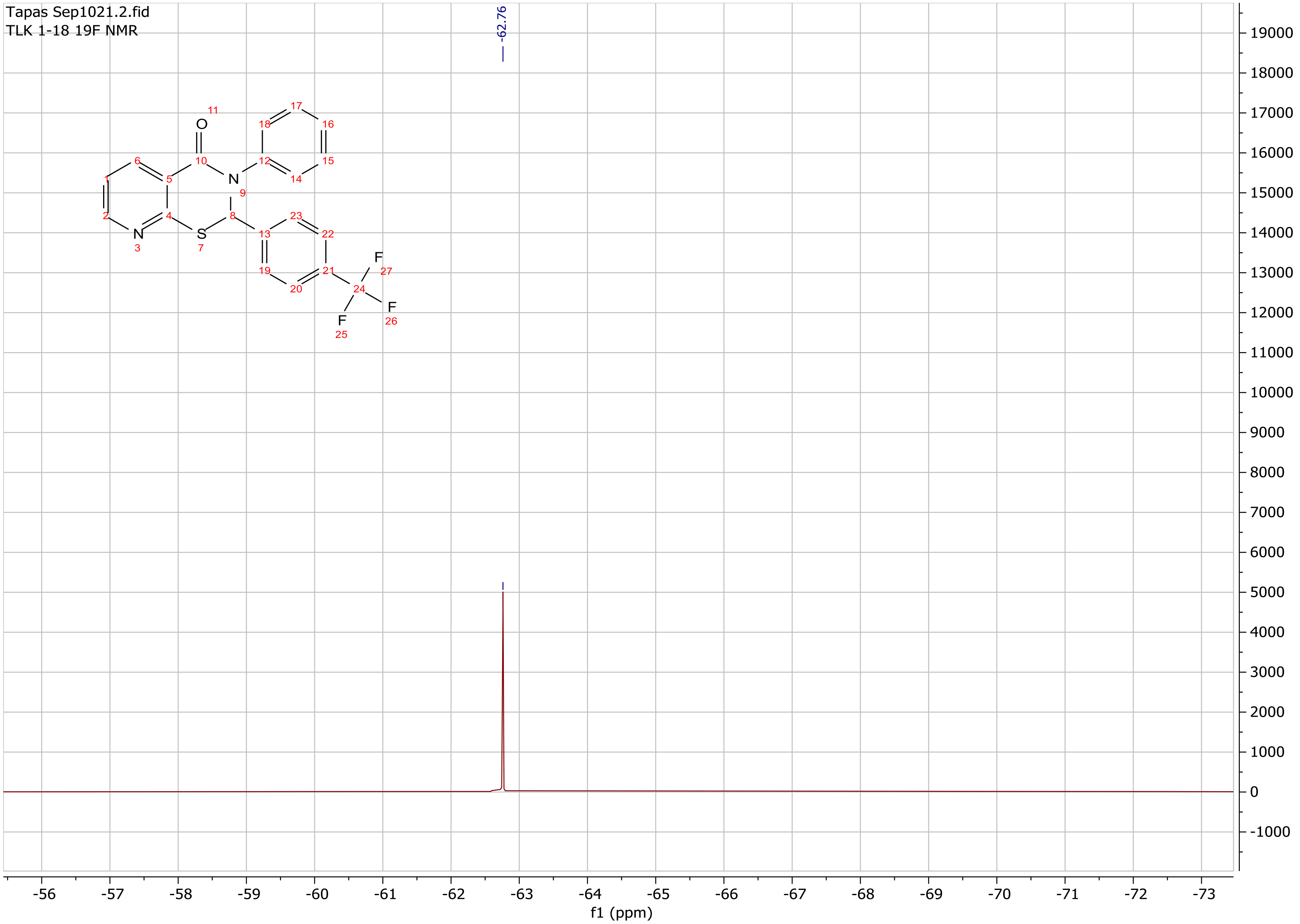

Figure S28. ^{19}F NMR spectrum of compound **1e** (*m*-CF_3).

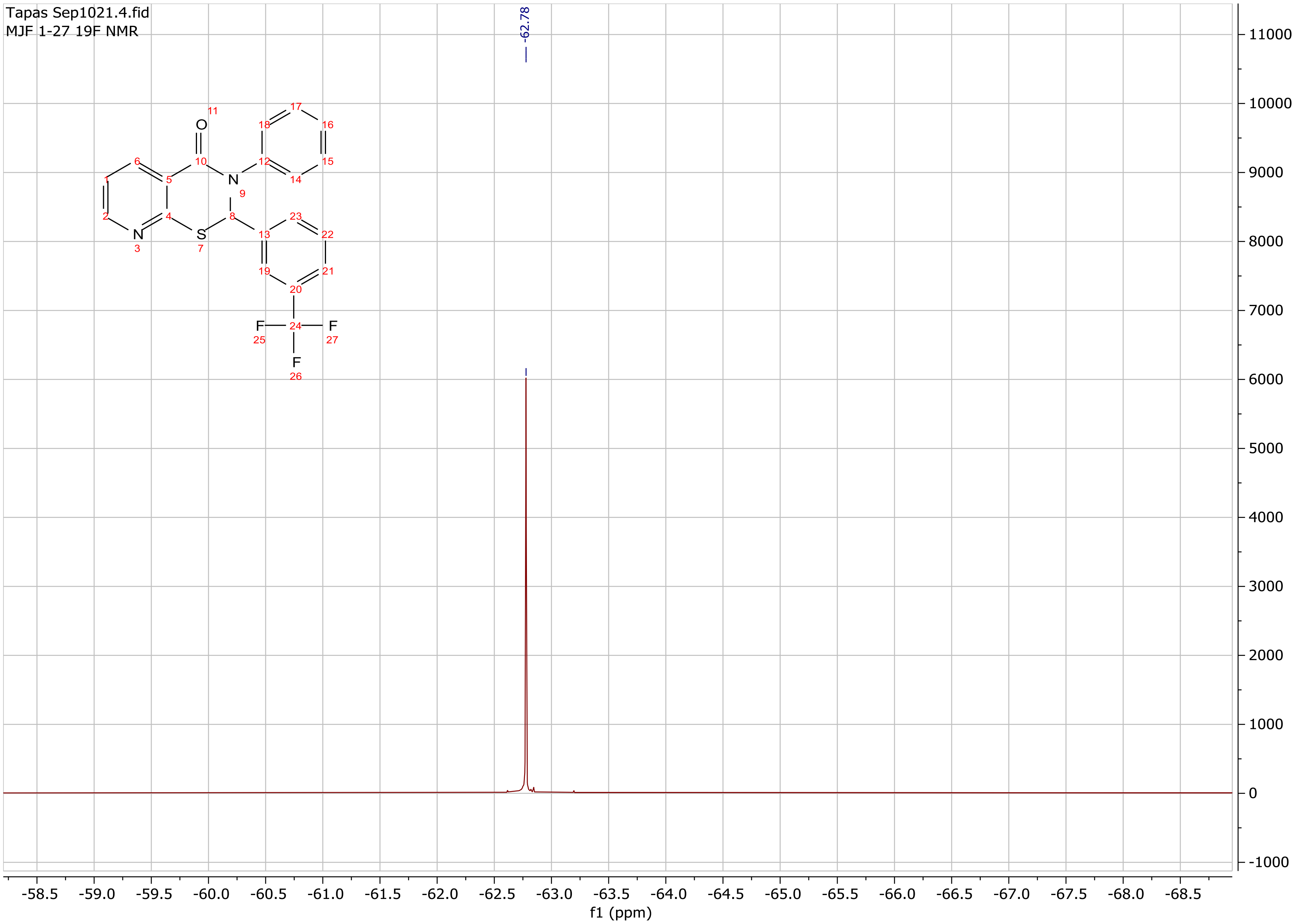

Figure S29. ^{19}F NMR spectrum of compound **1h** (*p*-F).

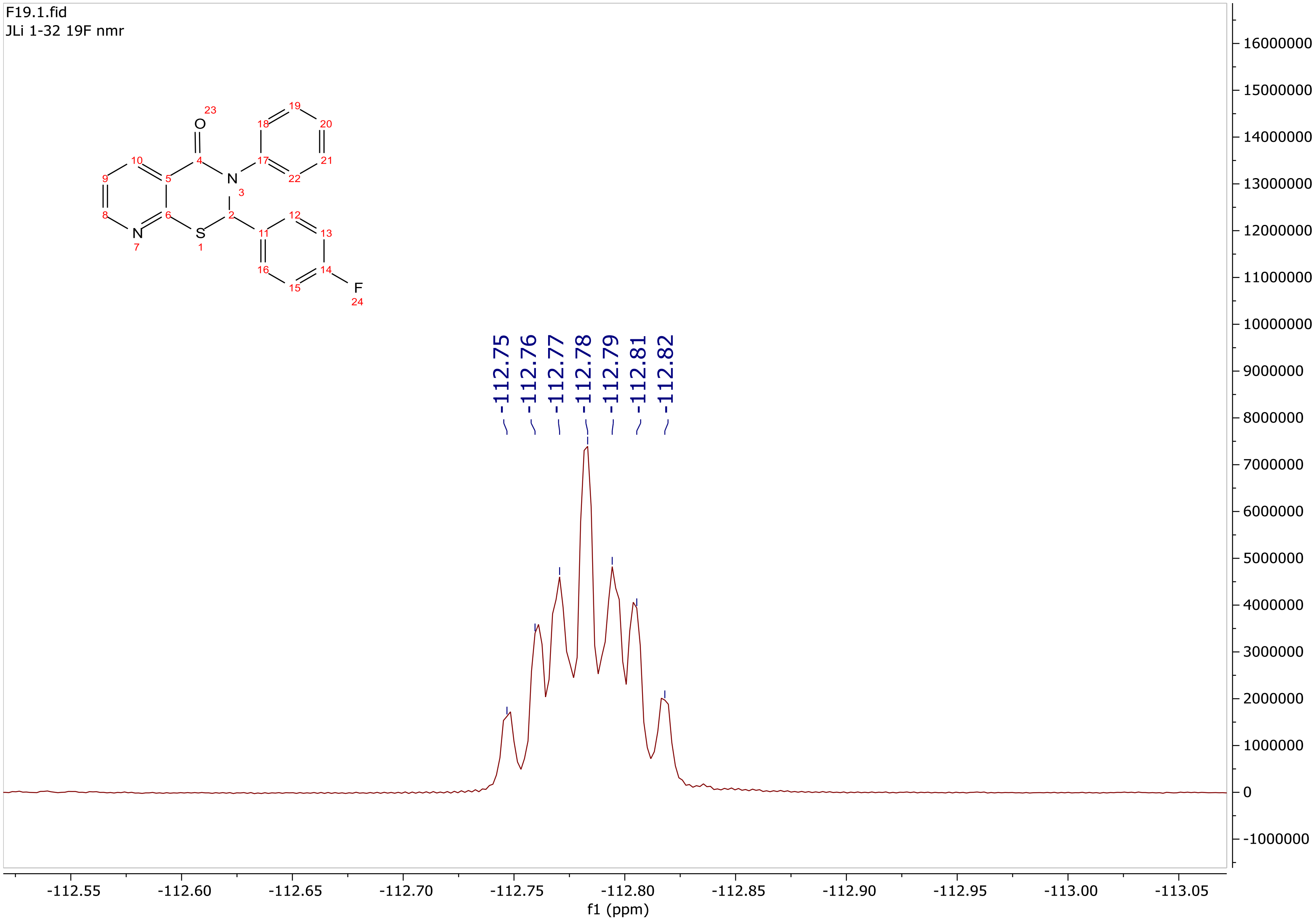

Figure S30. ^{19}F NMR spectrum of compound **1i** (*m*-F).

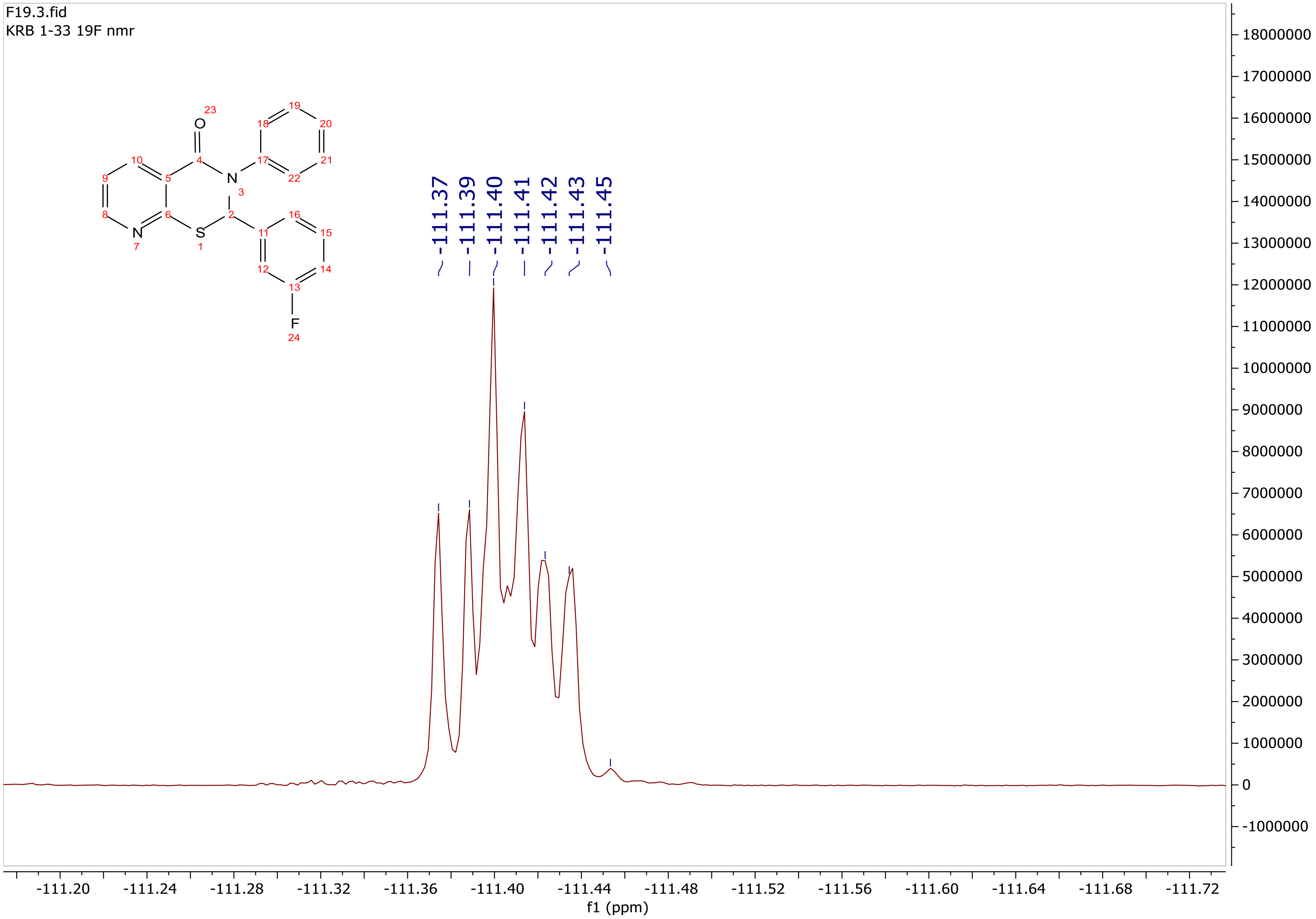

Figure S31. FT-IR spectrum of compound **1a** (*p*-NO_2).

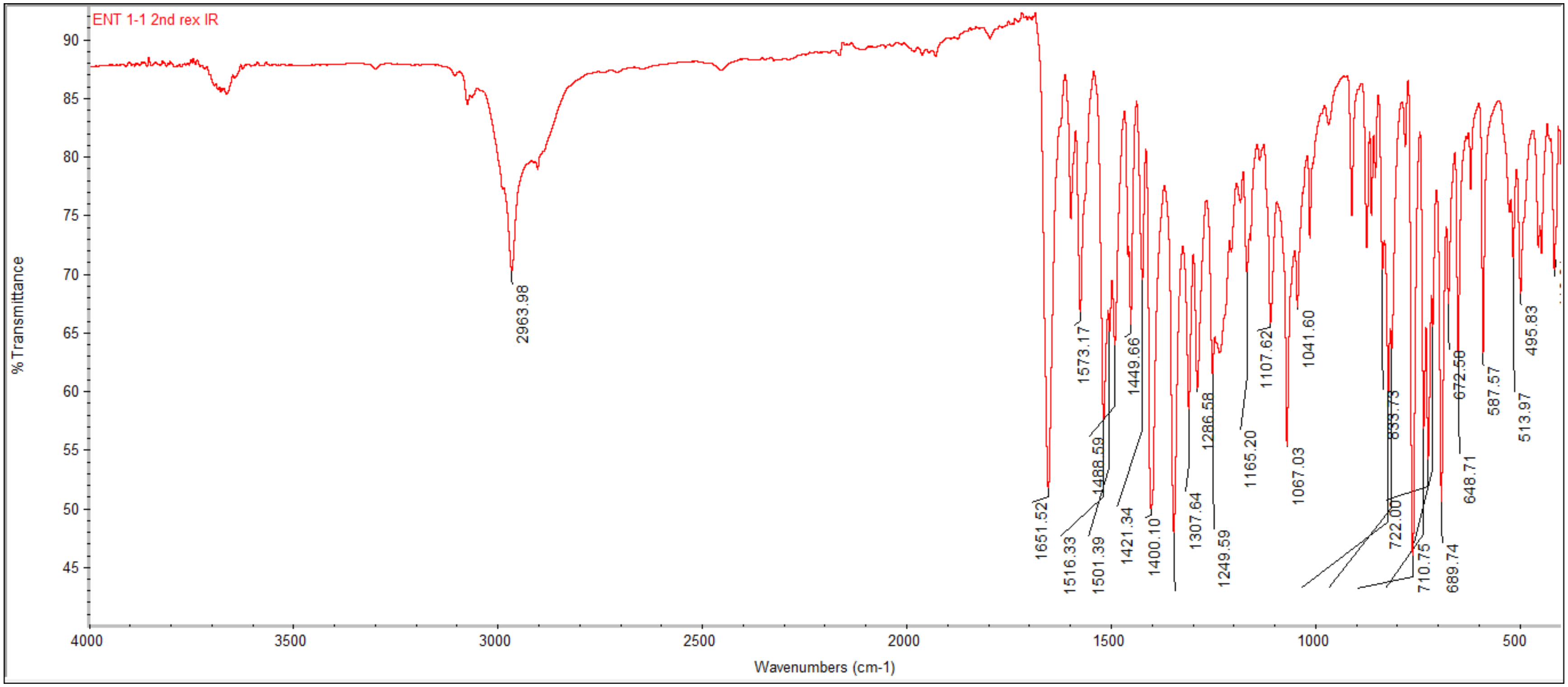

Figure S32. FT-IR spectrum of compound **1b** (*m*-NO_2).

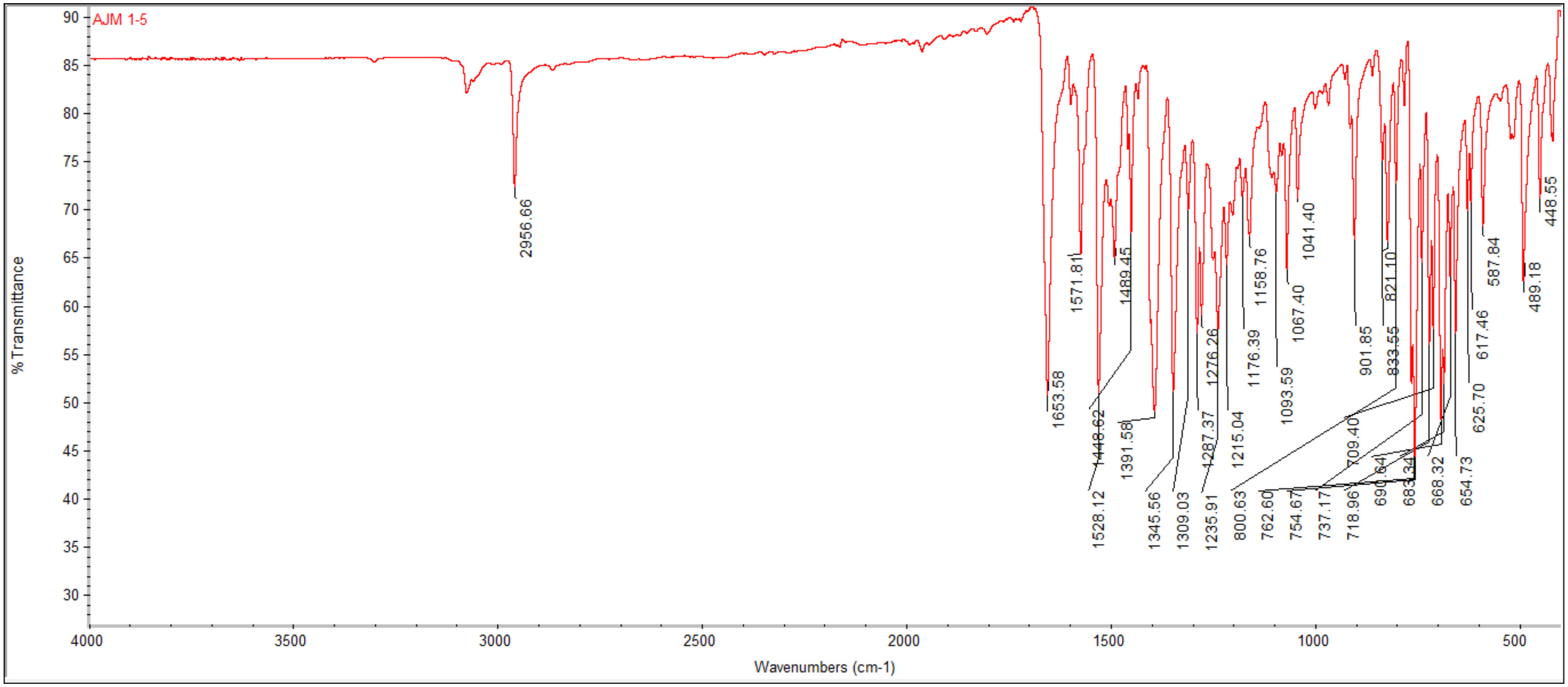

Figure S33. FT-IR spectrum of compound **1c** (*o*-NO_2).

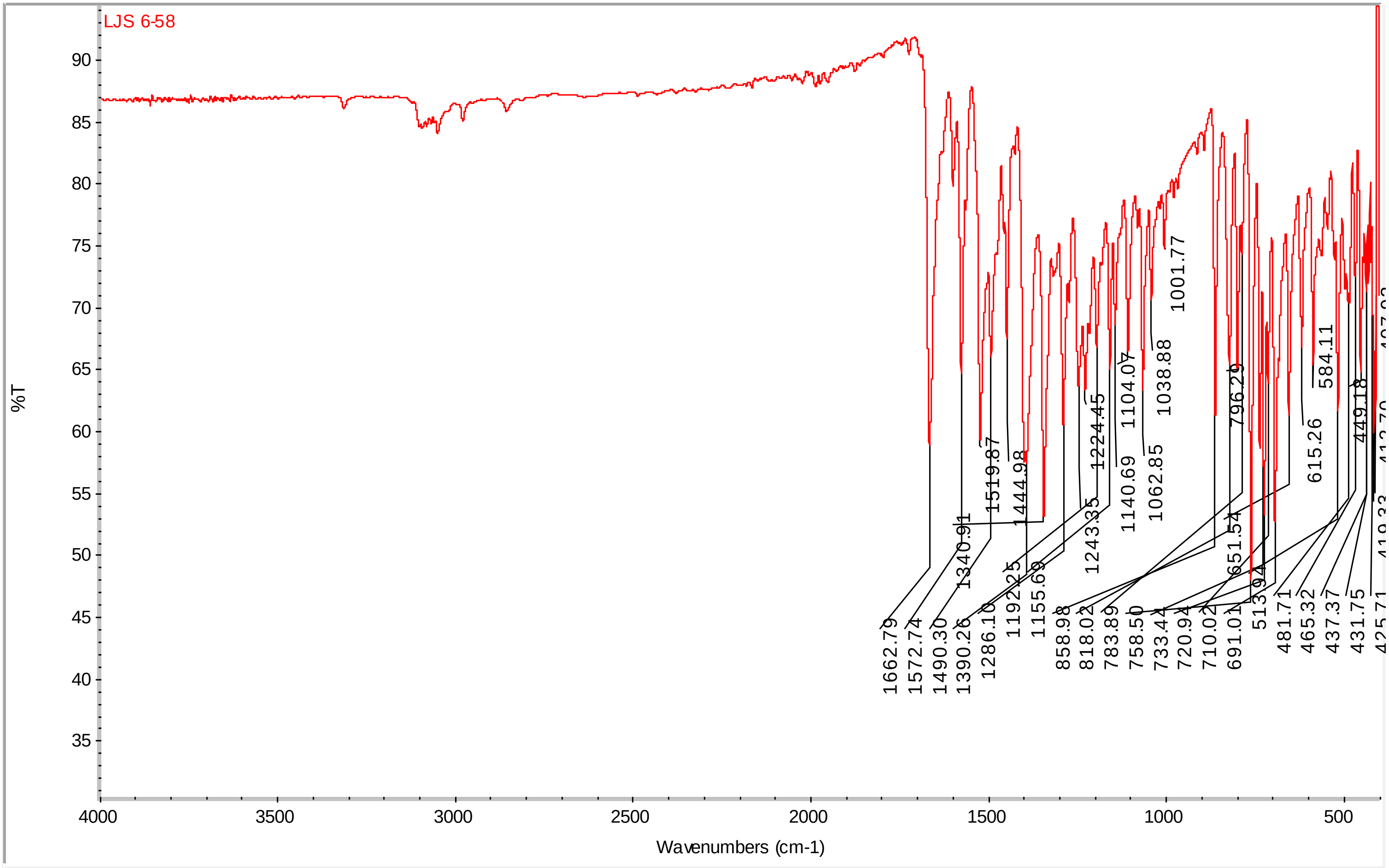

Figure S34. FT-IR spectrum of compound **1d** (*p*-CF_3).

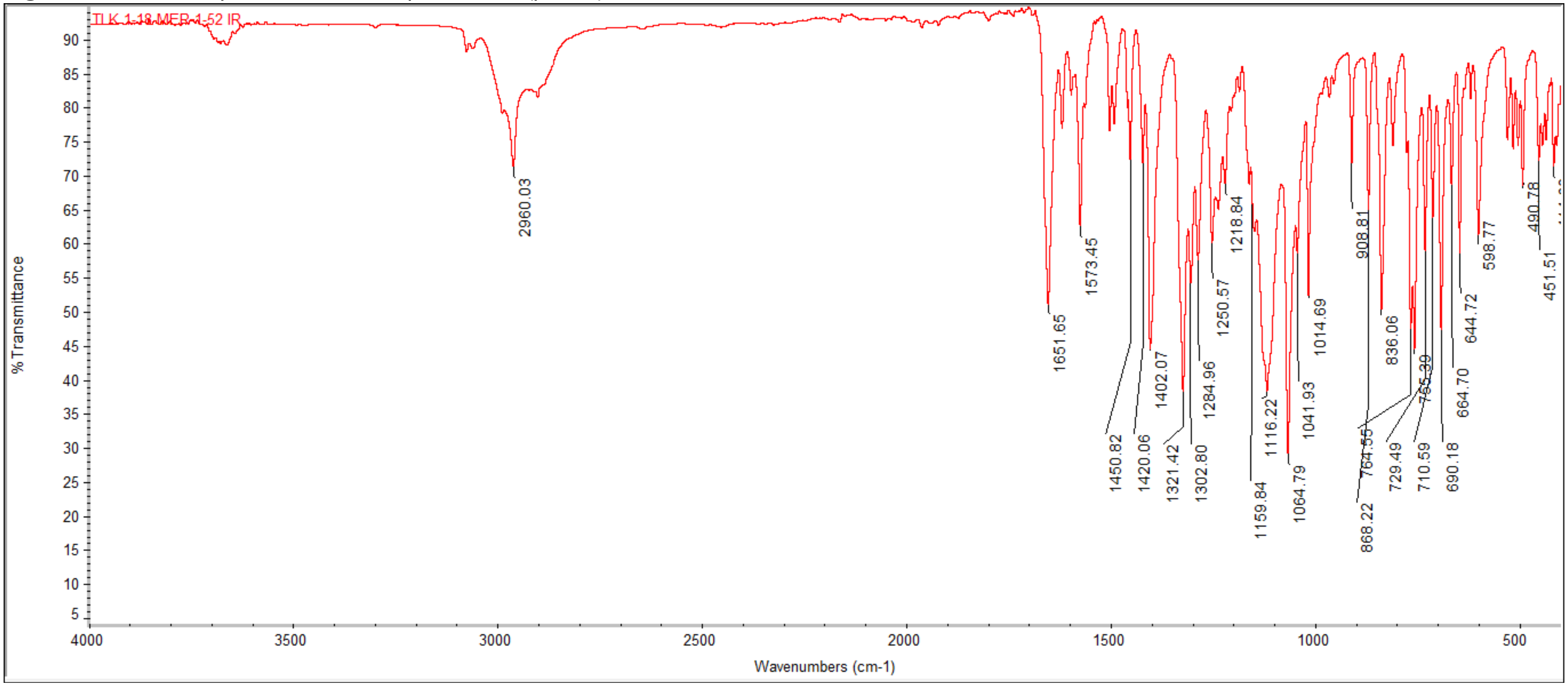

Figure S35. FT-IR spectrum of compound **1e** (*m*-CF_3).

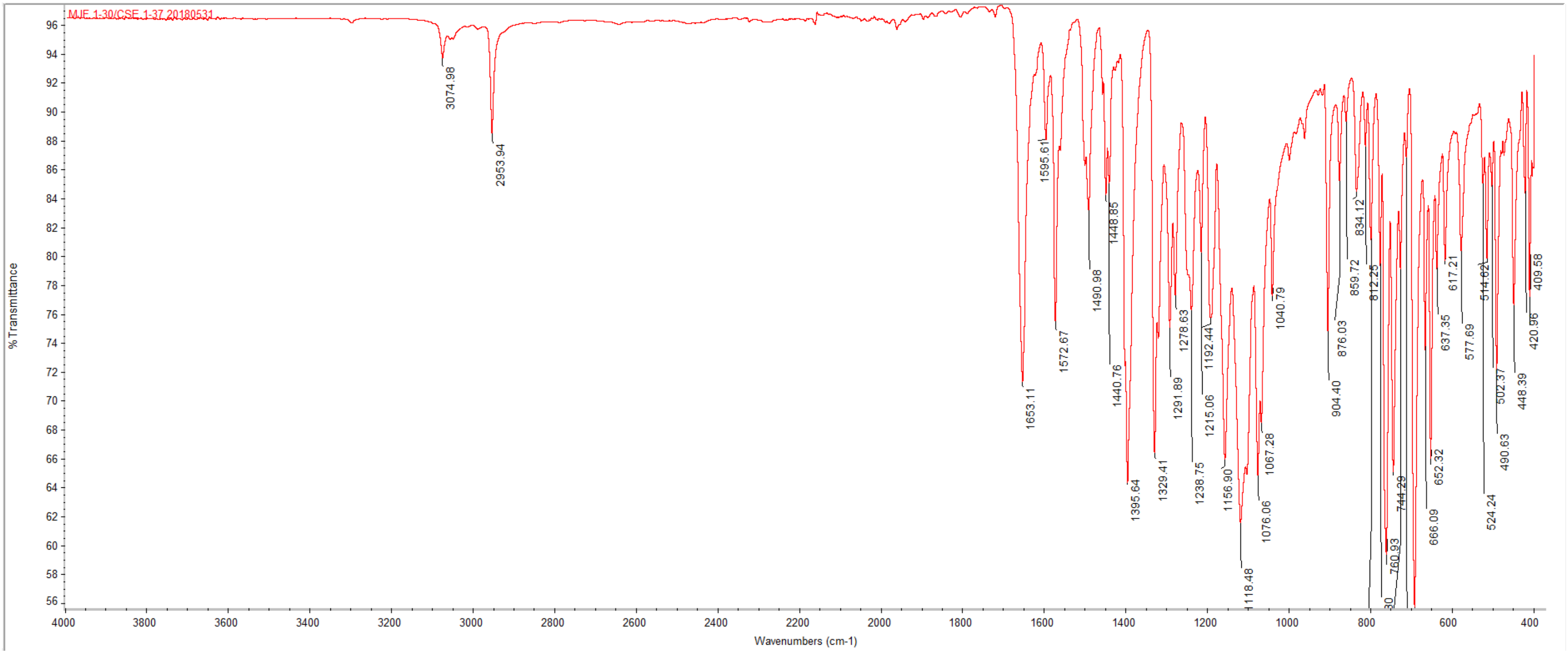

Figure S36. FT-IR spectrum of compound **1f** (*p*-Br).

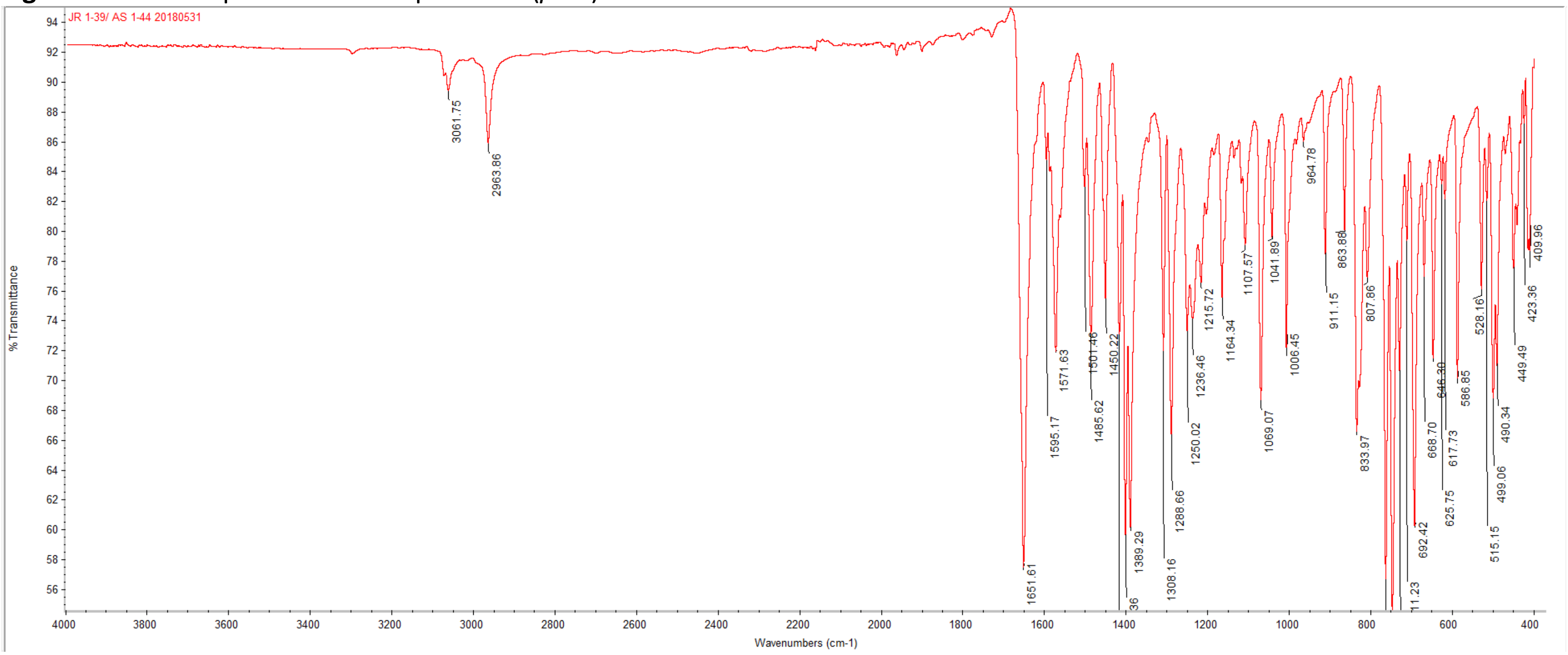

Figure S37. FT-IR spectrum of compound **1g** (*m*-Br).

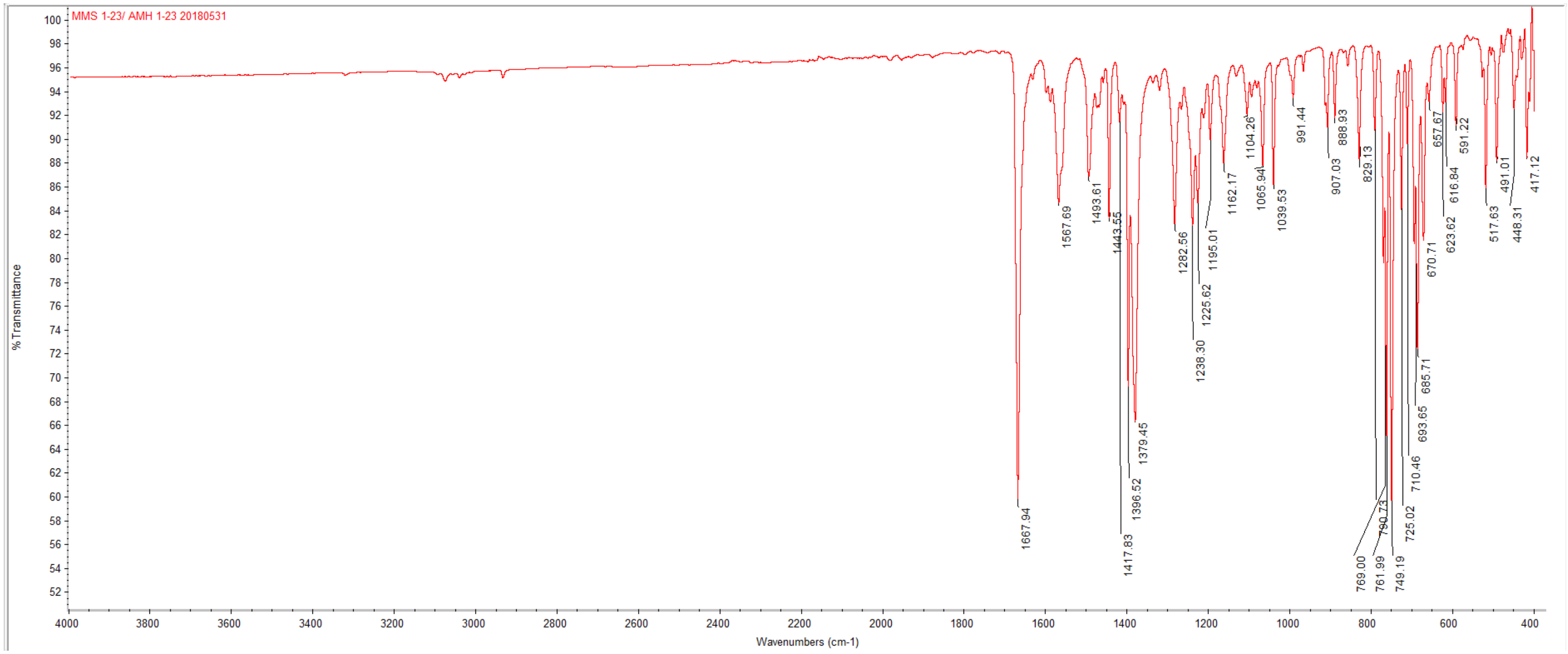

Figure S38. FT-IR spectrum of compound **1h** (*p*-F).

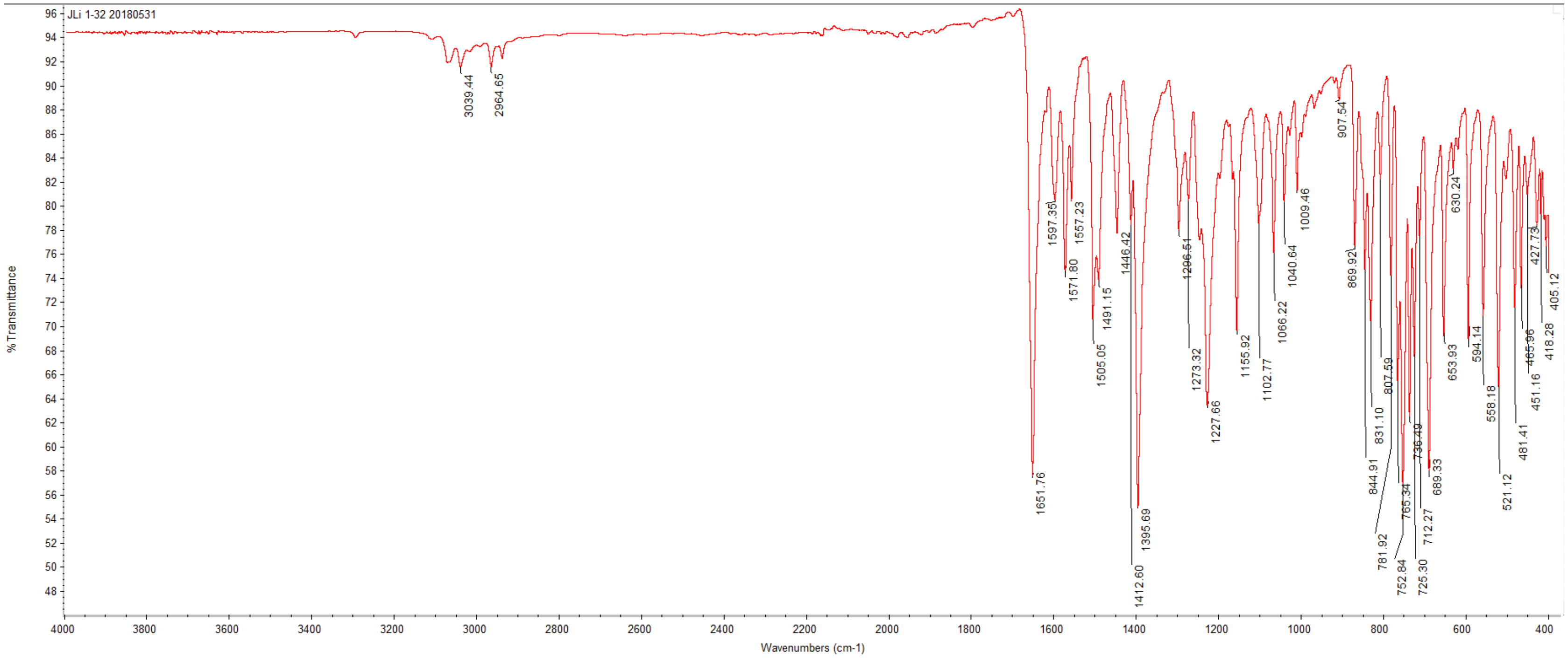

Figure S39. FT-IR spectrum of compound **1i** (*m*-F).

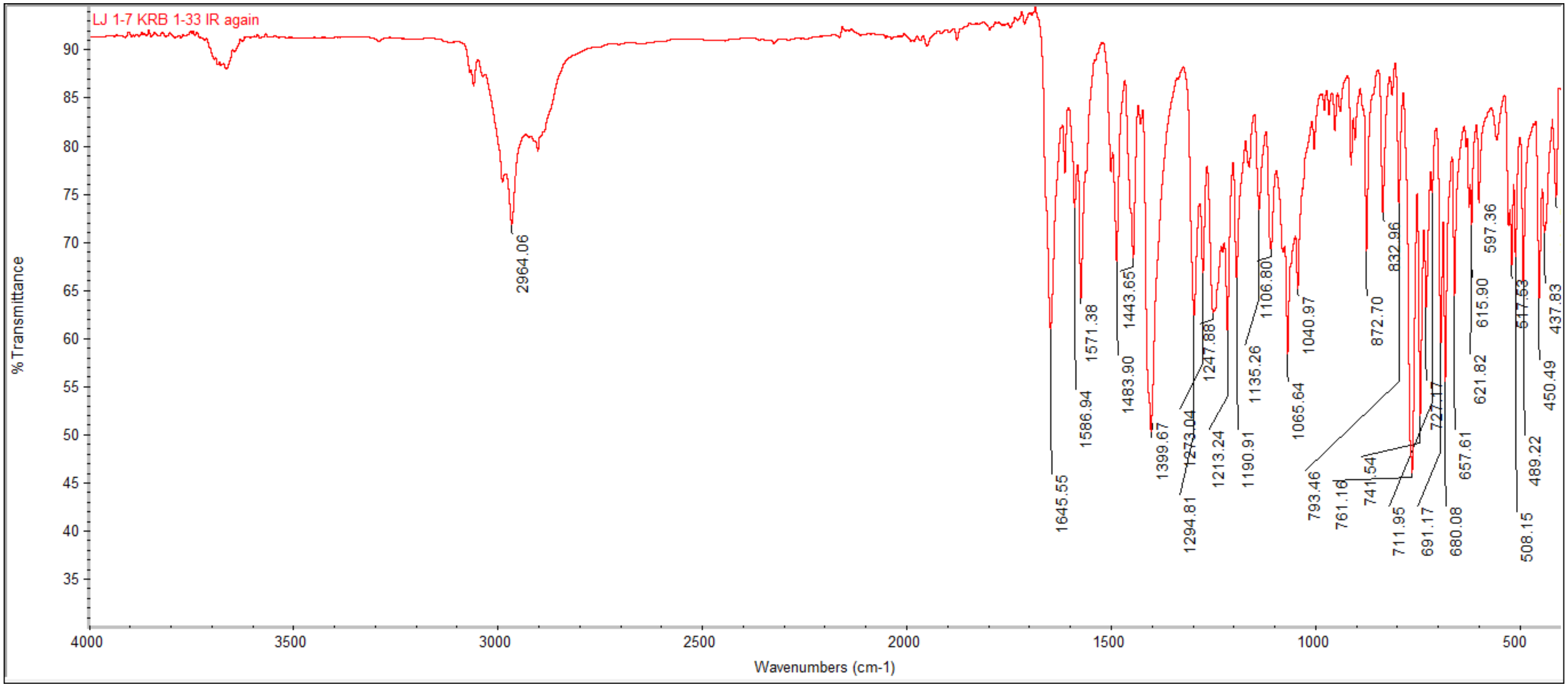

Figure S40. FT-IR spectrum of compound **1k** (*p*-Me).

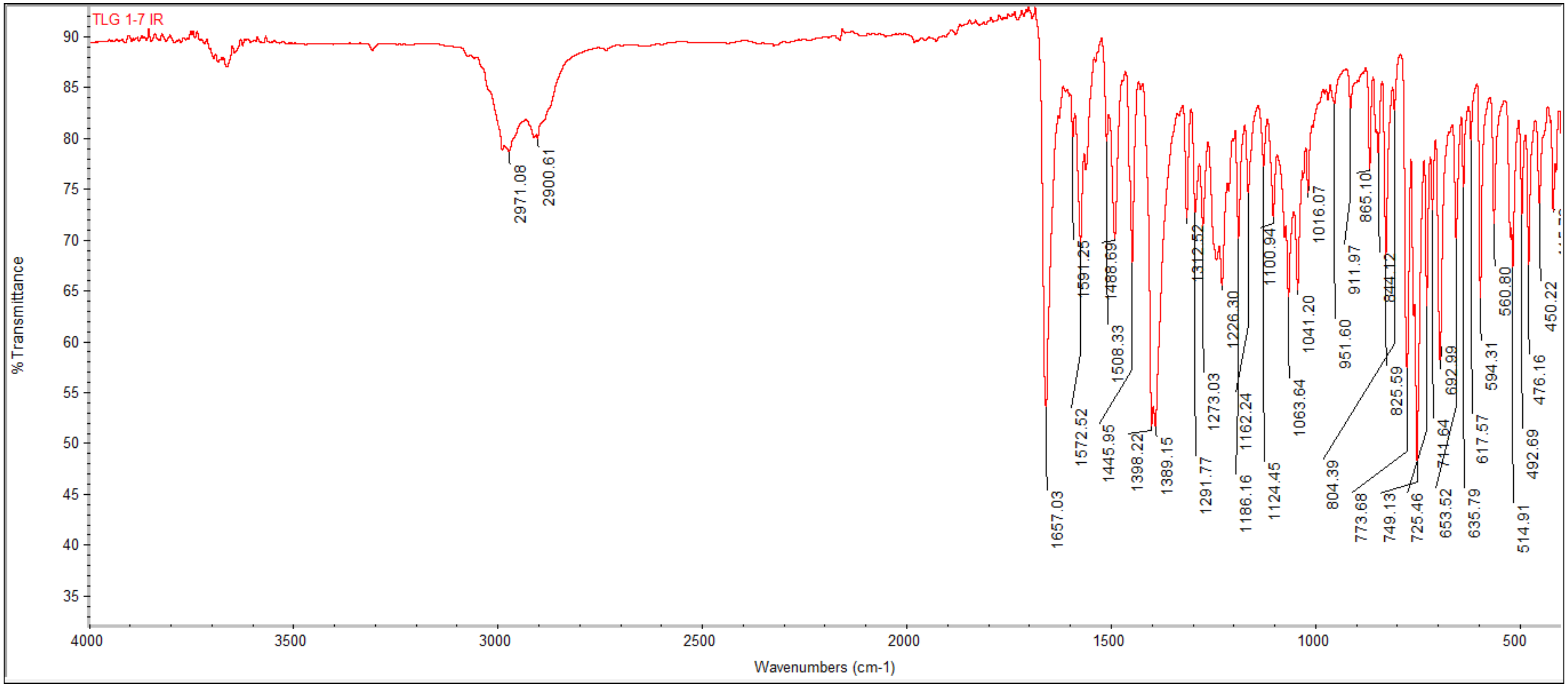

Figure S41. FT-IR spectrum of compound **1l** (*m*-Me).

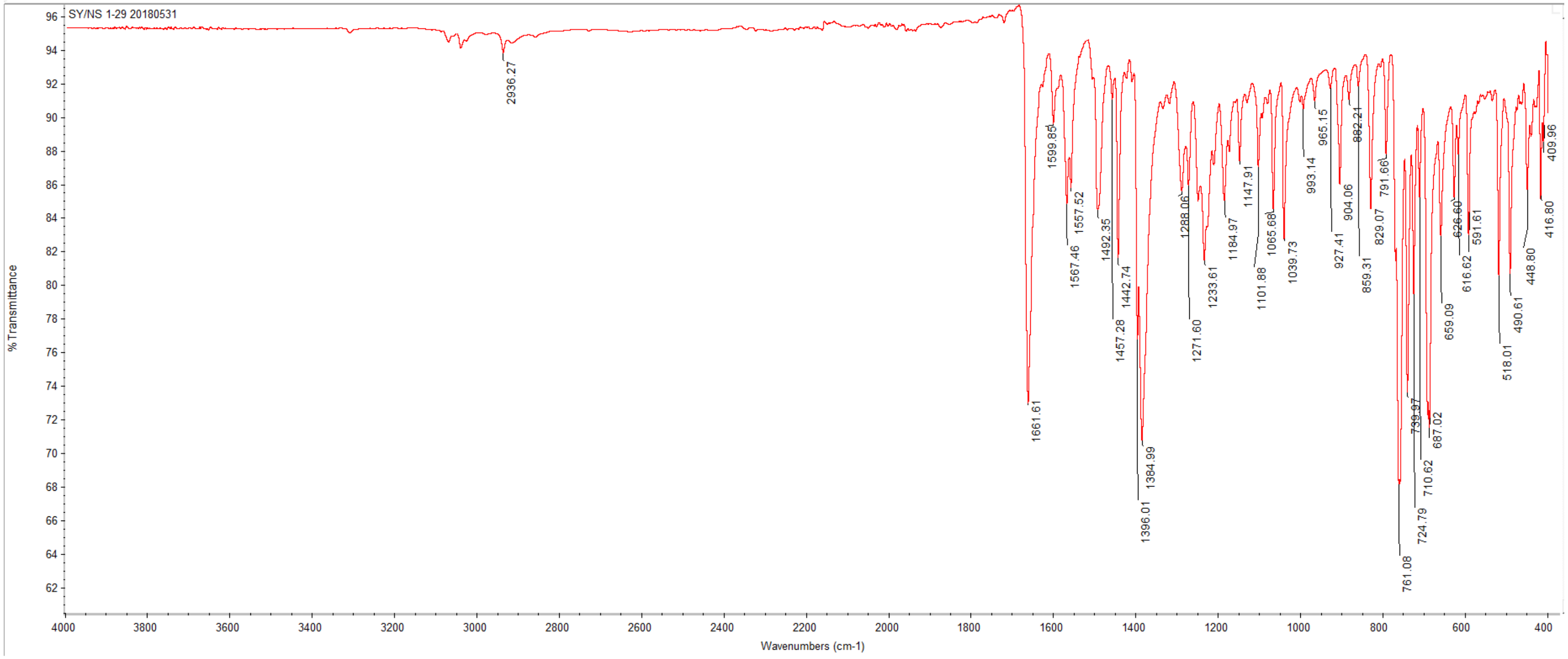

Figure S42. FT-IR spectrum of compound **1m** (*p*-OMe).

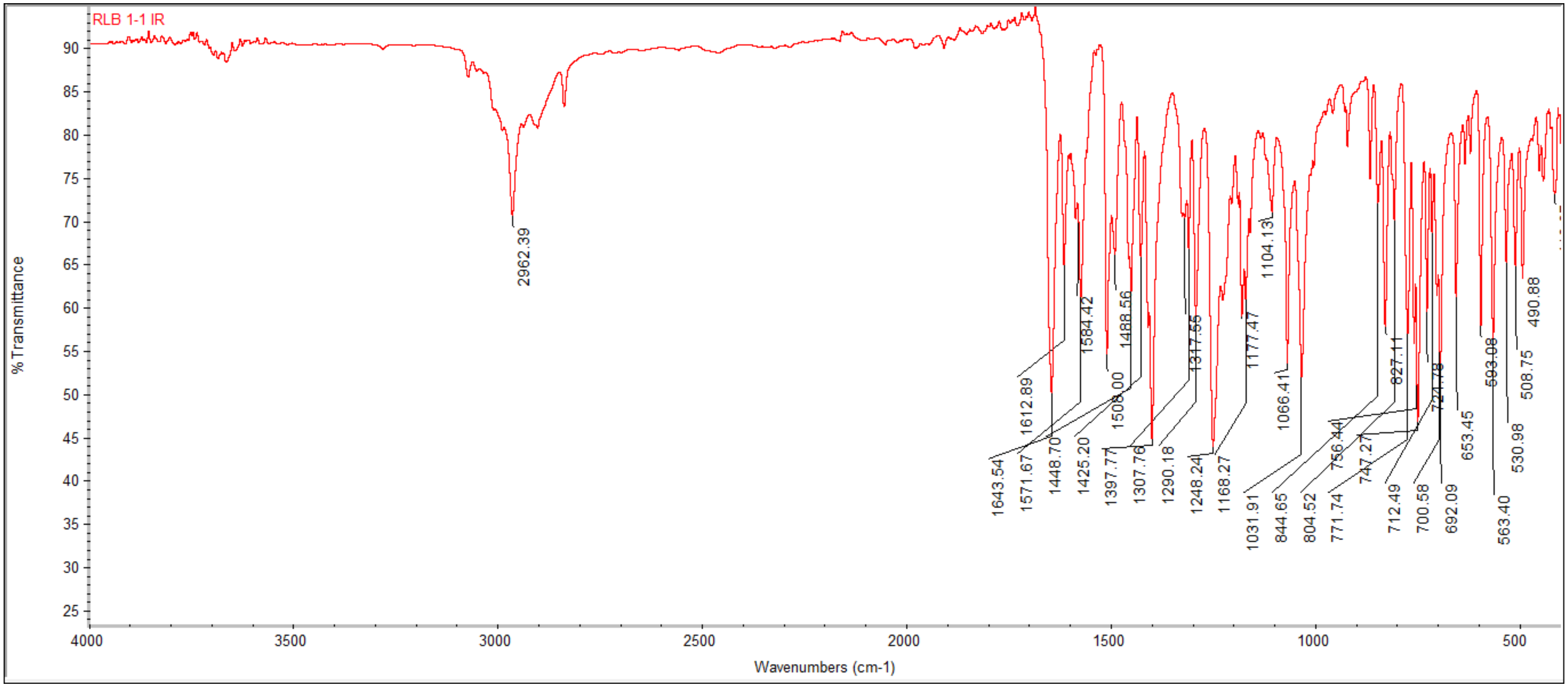

Figure S43. FT-IR spectrum of compound **1n** (*m*-OMe).

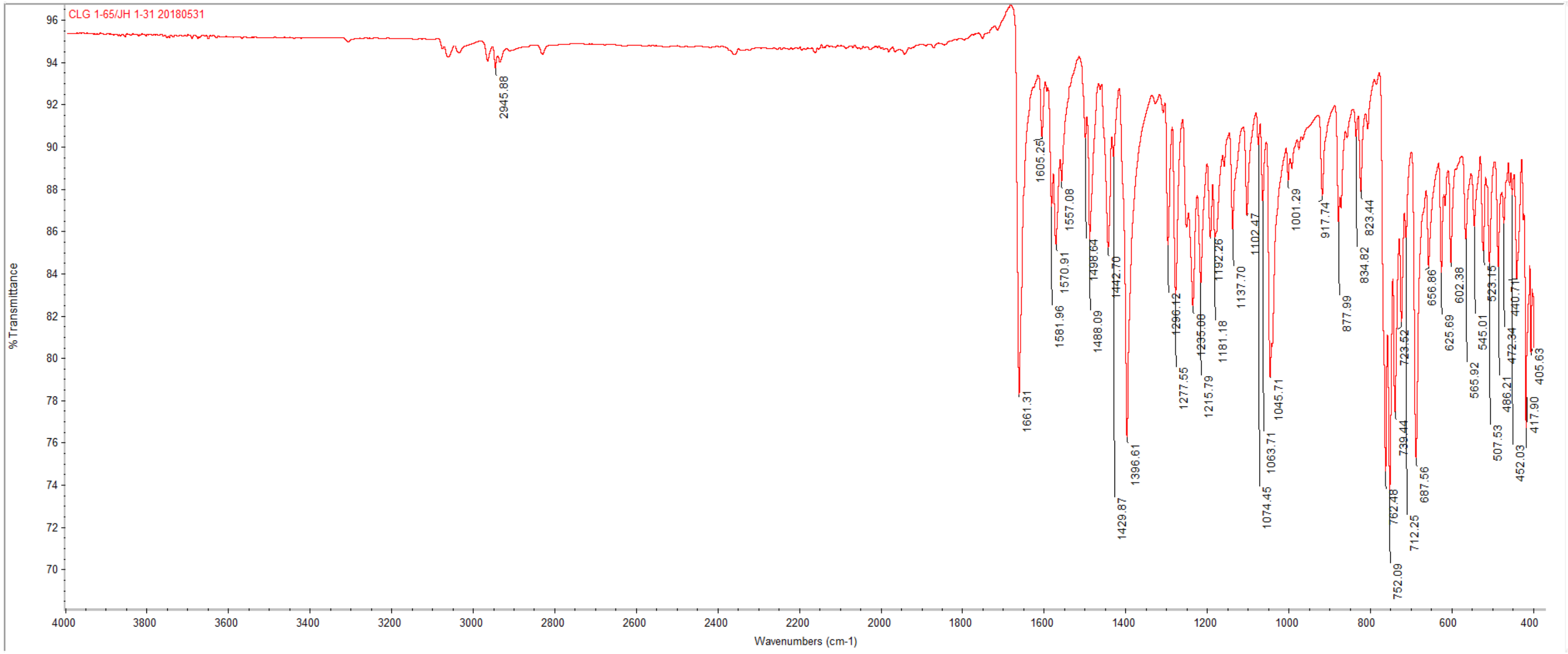

Figure S44. Mass spectrum of compound **1a** (*p*-NO_2).

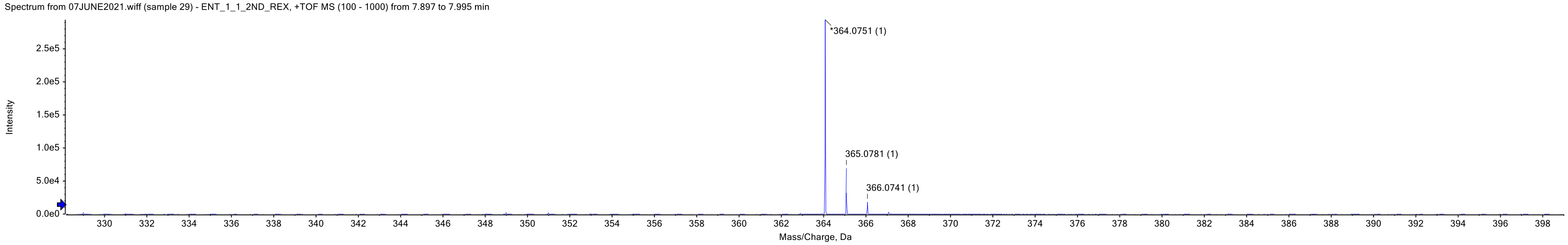

[M+H]$^+$ 364.0751Da

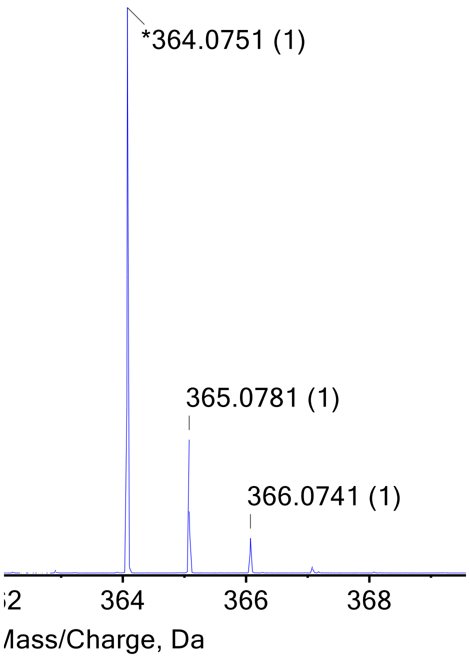

Figure S45. Mass spectrum of compound **1b** (*m*-NO_2).

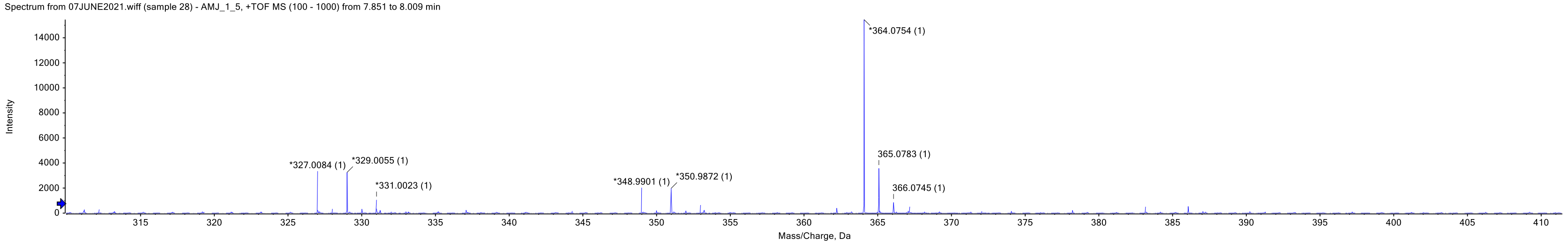

$[M+H]^+$ 364.0754Da

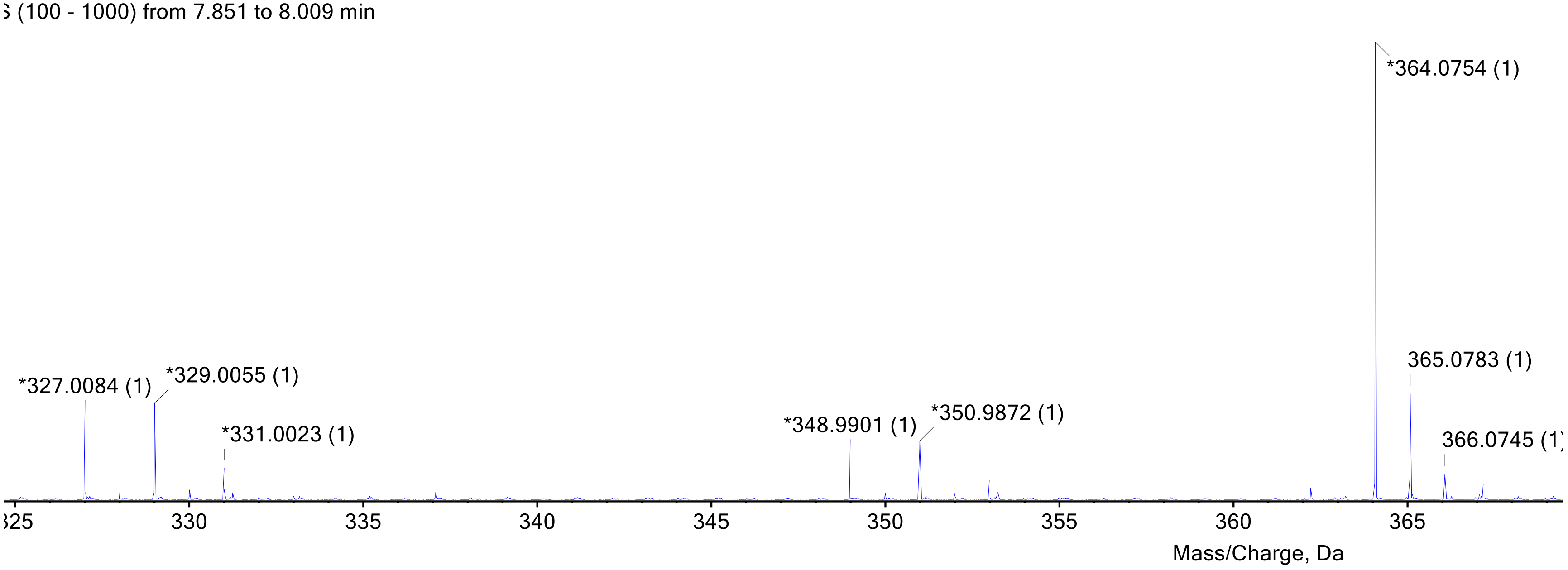

Figure S46. Mass spectrum of compound **1c** (*o*-NO_2).

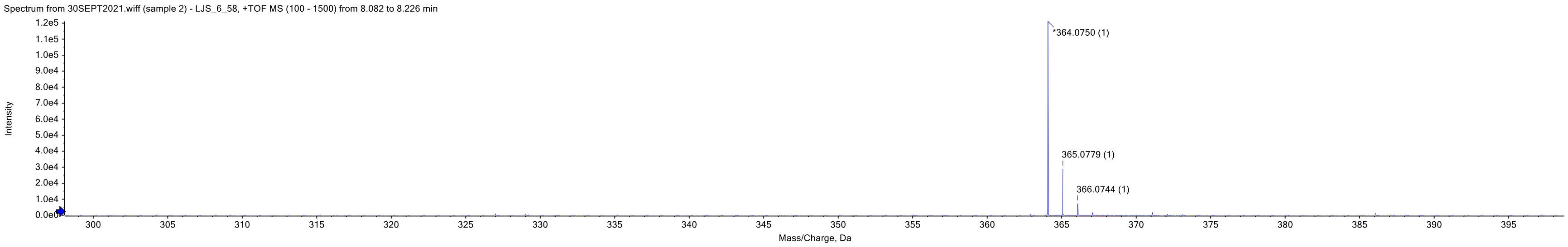

[M+H]$^+$ 364.0750

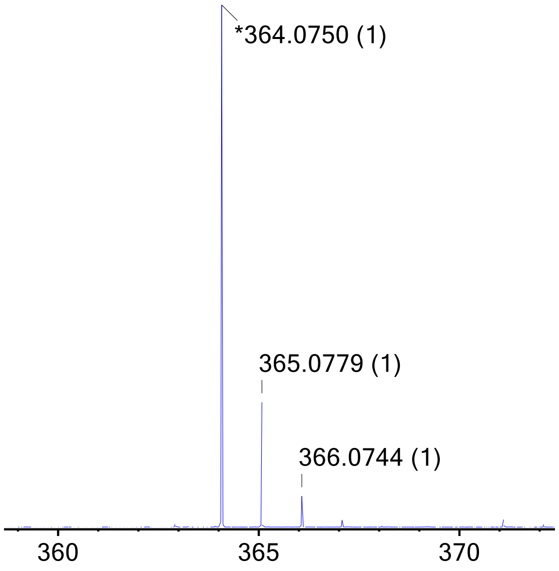

Figure S47. Mass spectrum of compound **1d** (*p*-CF_3).

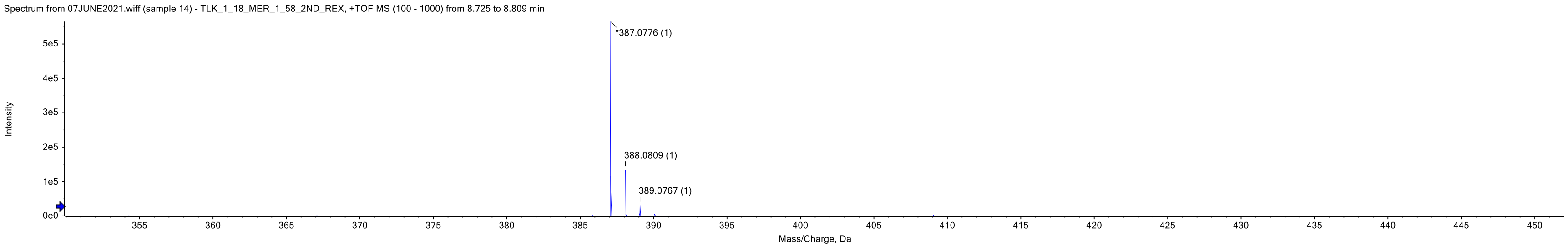

$[M+H]^+$ 387.0076Da

o 8.809 min

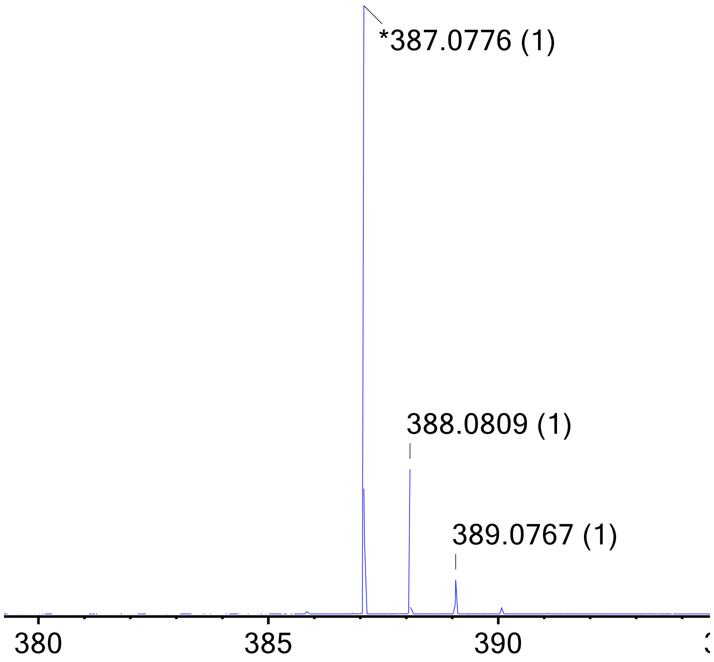

Figure S48. Mass spectrum of compound **1e** (*m*-CF_3).

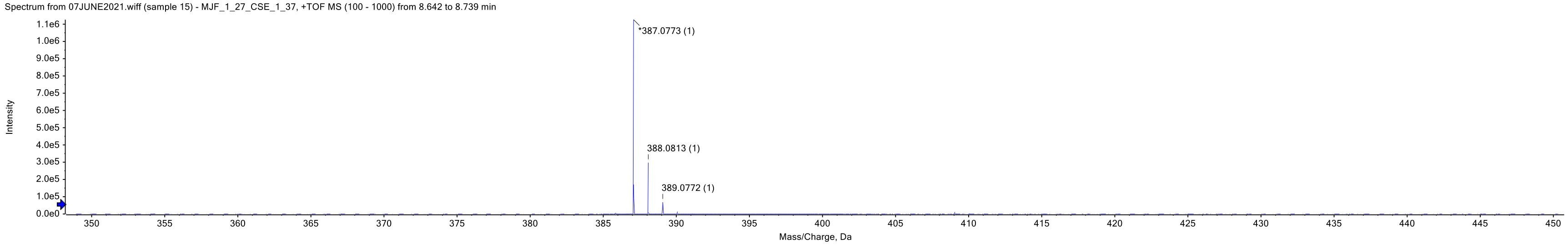

[M+H]$^+$ 387.0773Da

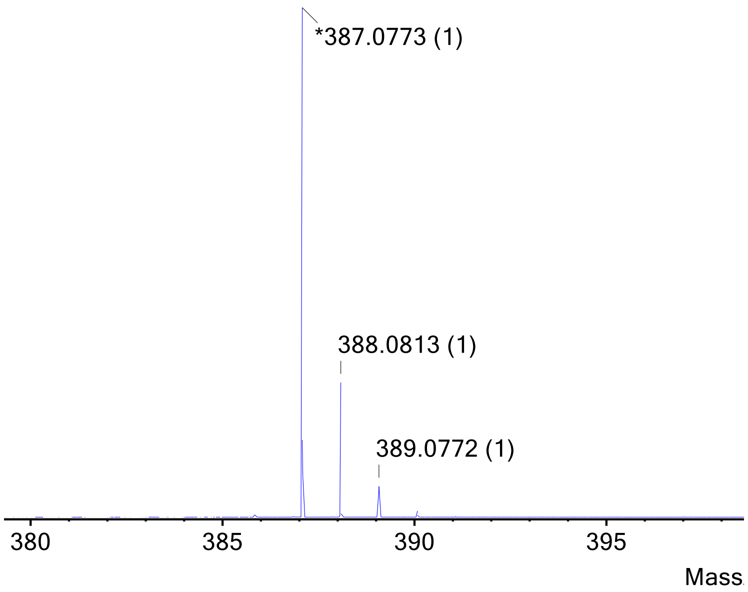

Figure S49. Mass spectrum of compound **1f** (*p*-Br).

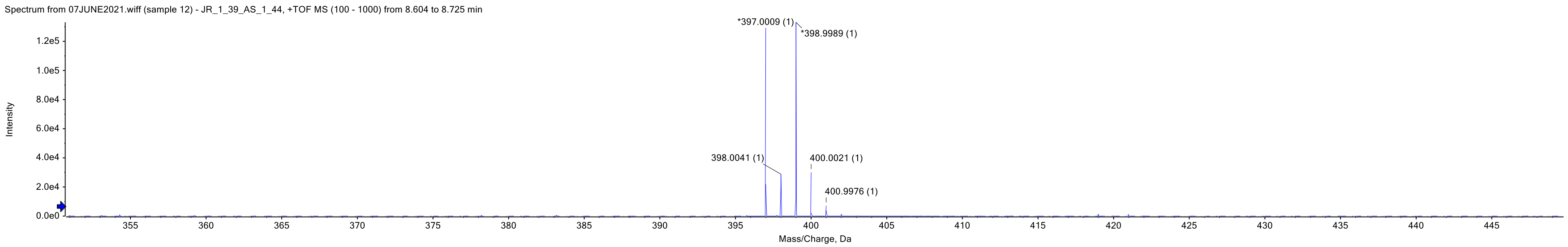

[M+H]$^+$ 397.0009Da

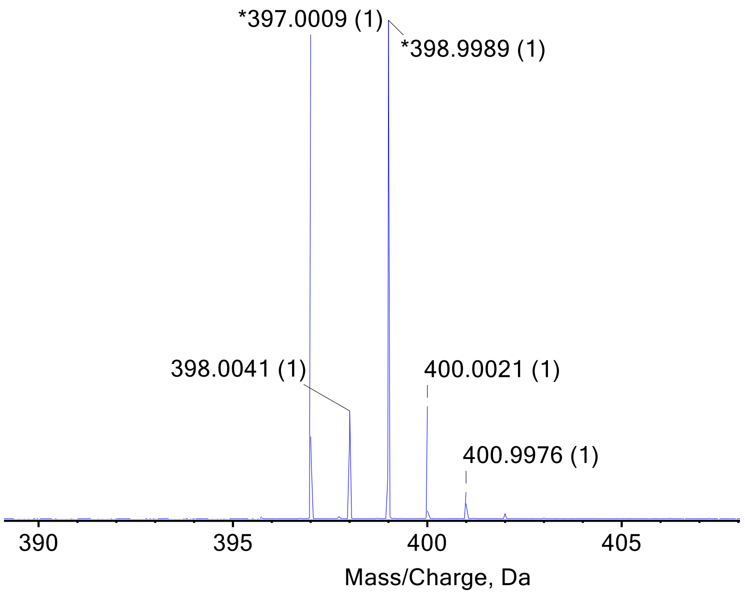

Figure S50. Mass spectrum of compound **1g** (*m*-Br).

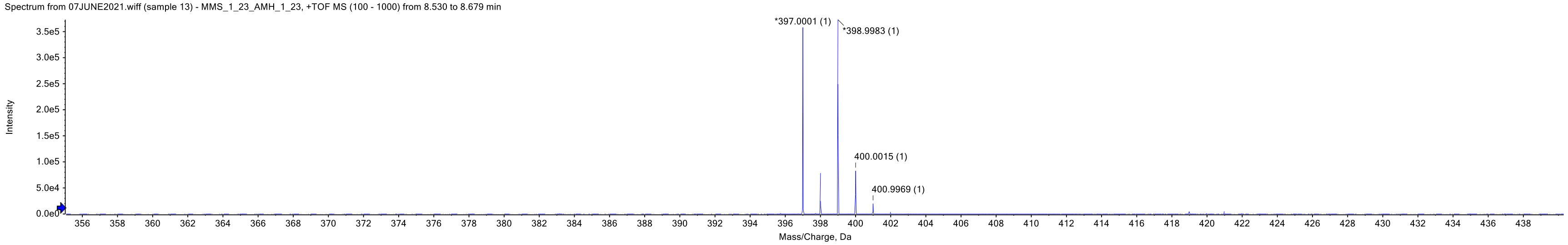

[M+H]$^{+}$ 397.0001Da

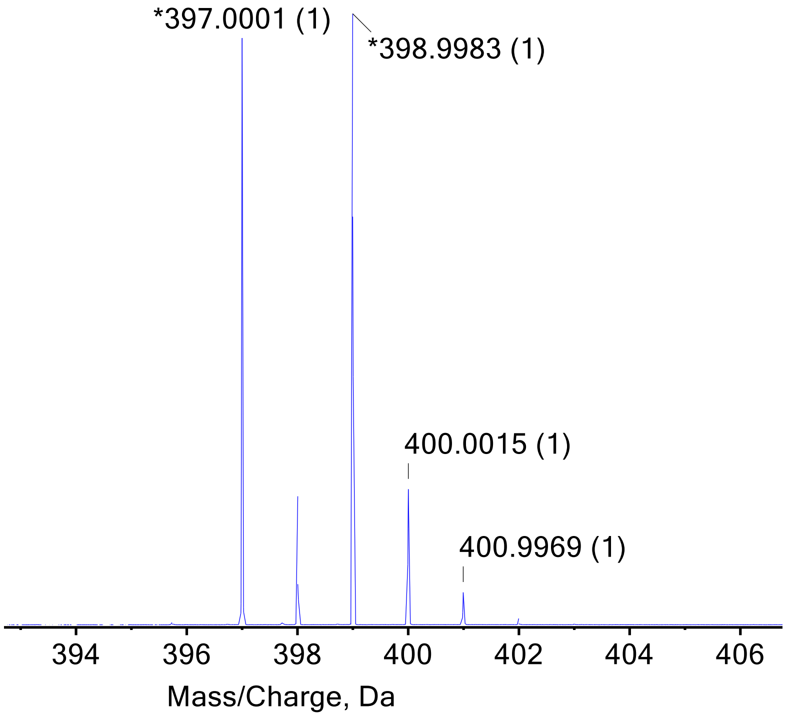

Figure S51. Mass spectrum of compound **1h** (*p*-F).

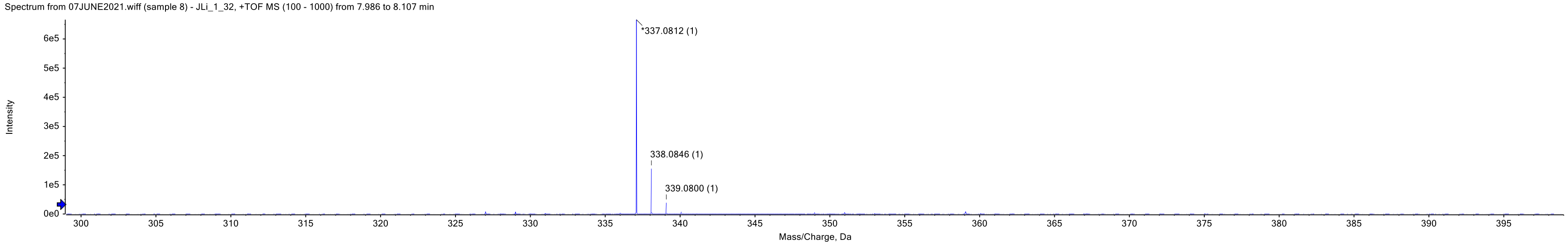

[M+H]$^{+}$ 337.0812Da

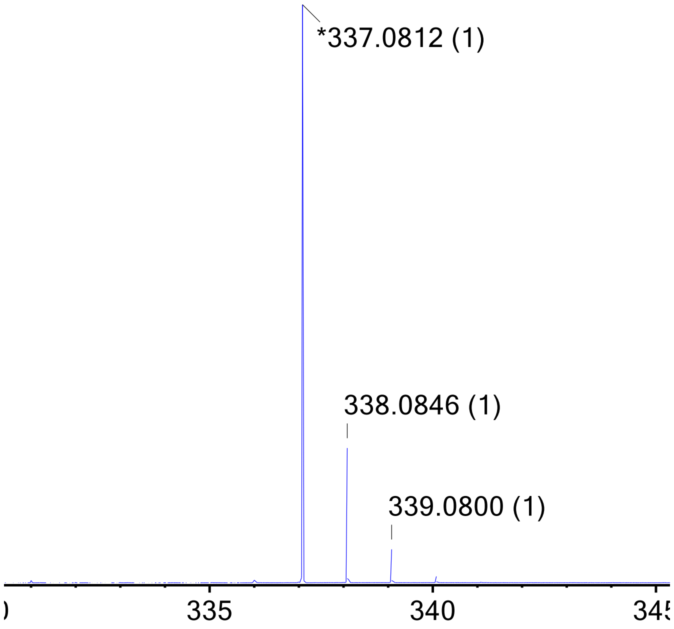

Figure S52. Mass spectrum of compound **1i** (*m*-F).

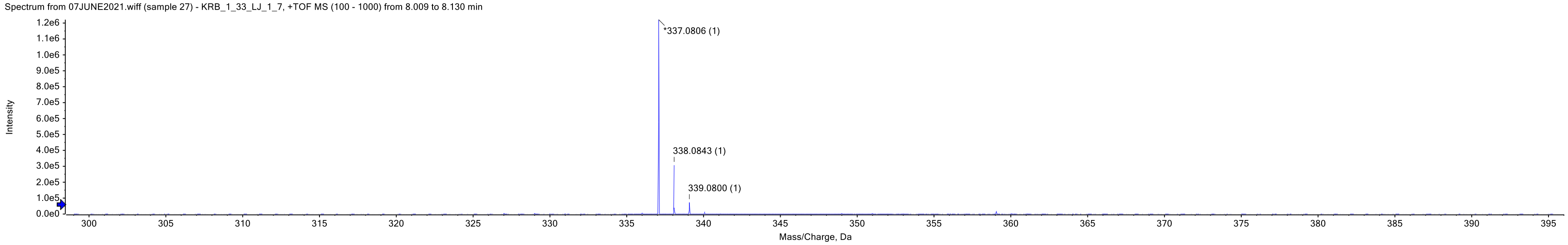

$[M+H]^+$ 337.0806Da

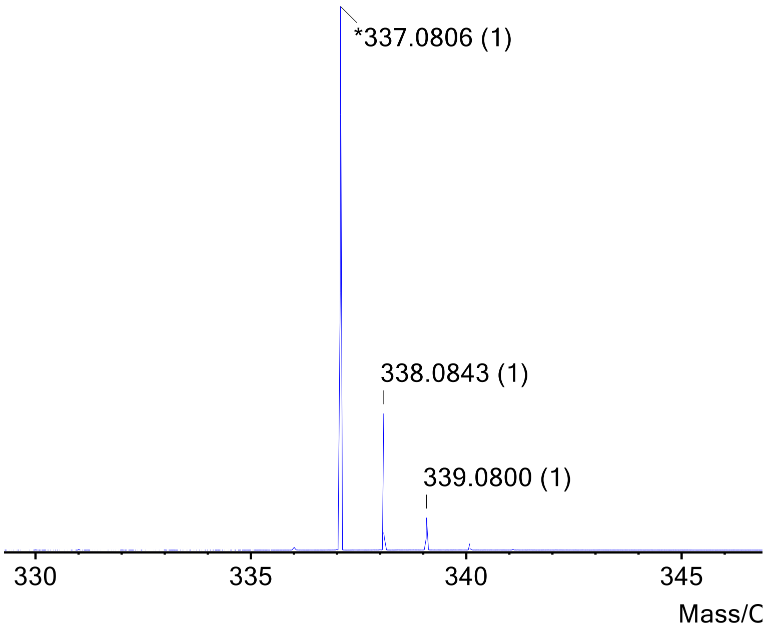

Figure S53. Mass spectrum of compound **1k** (*p*-Me).

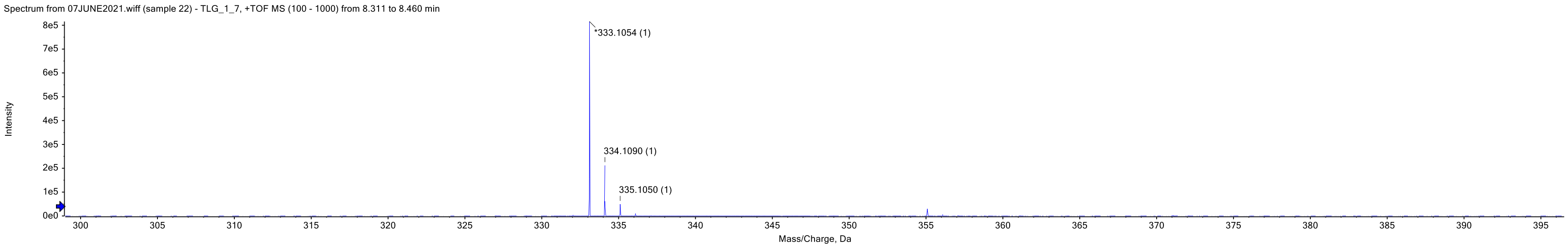

[M+H]$^{+}$ 333.1054Da

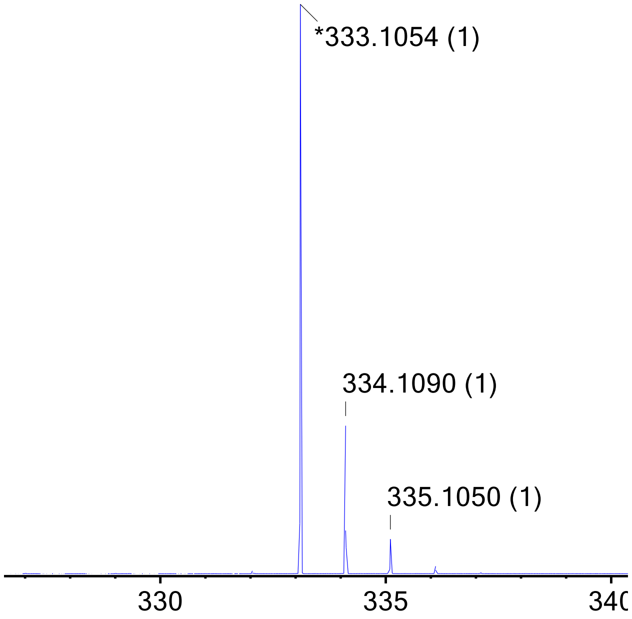

Figure S54. Mass spectrum of compound **1l** (*m*-Me).

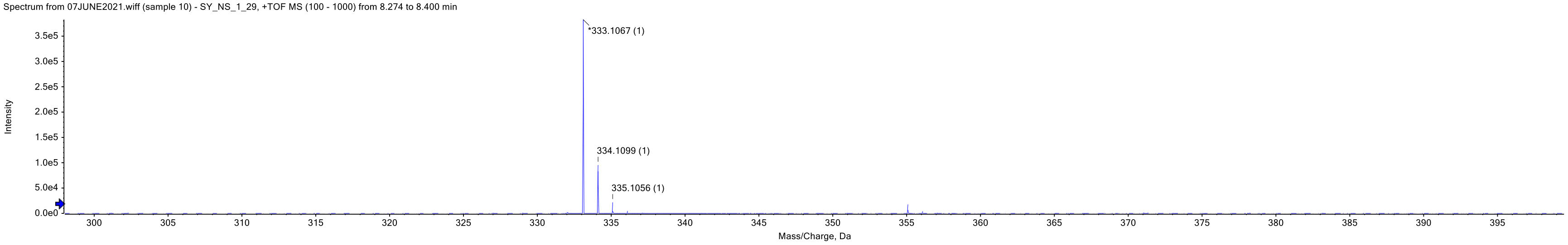

$[M+H]^+$ 333.1067Da

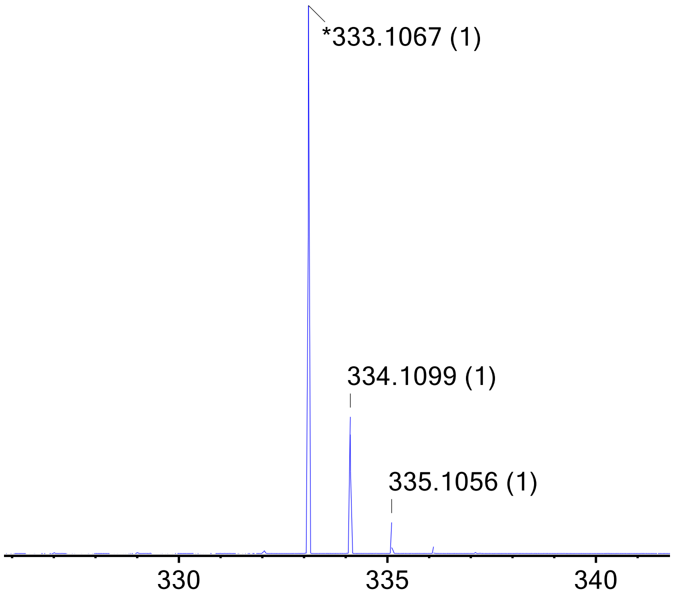

Figure S55. Mass spectrum of compound **1m** (*p*-OMe).

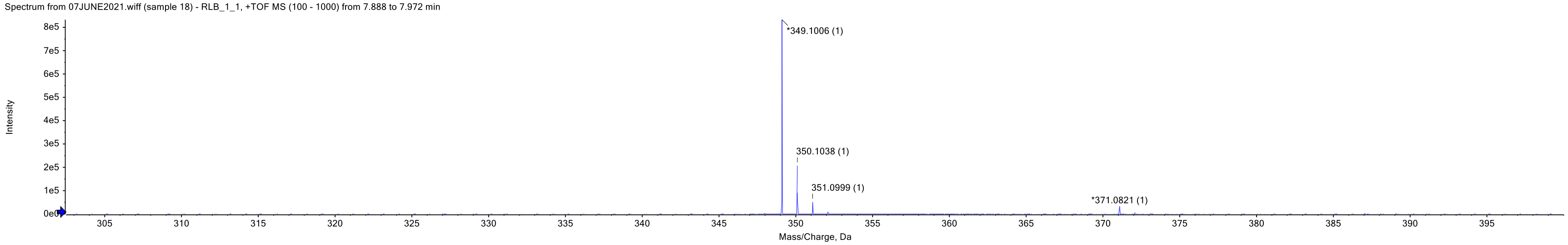

[M+H]$^+$ 349.1006Da

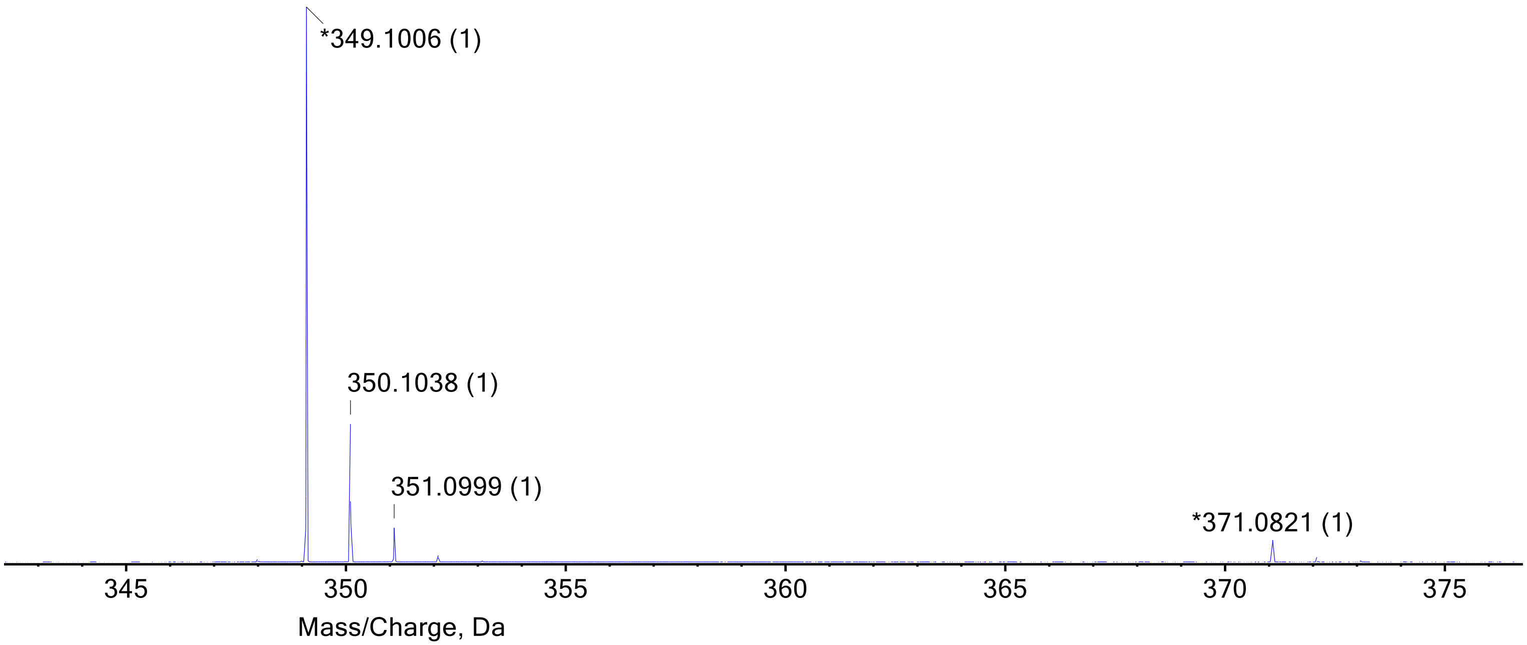

Figure S56. Mass spectrum of compound **1n** (*m*-OMe).

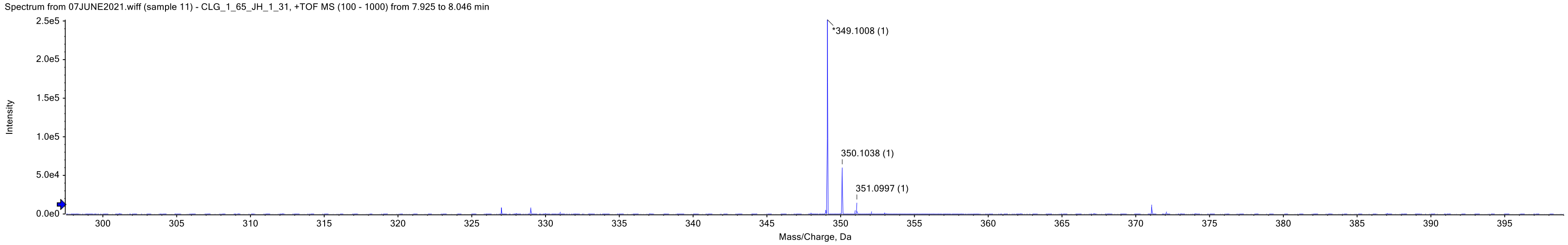

$[M+H]^+$ 349.1008Da

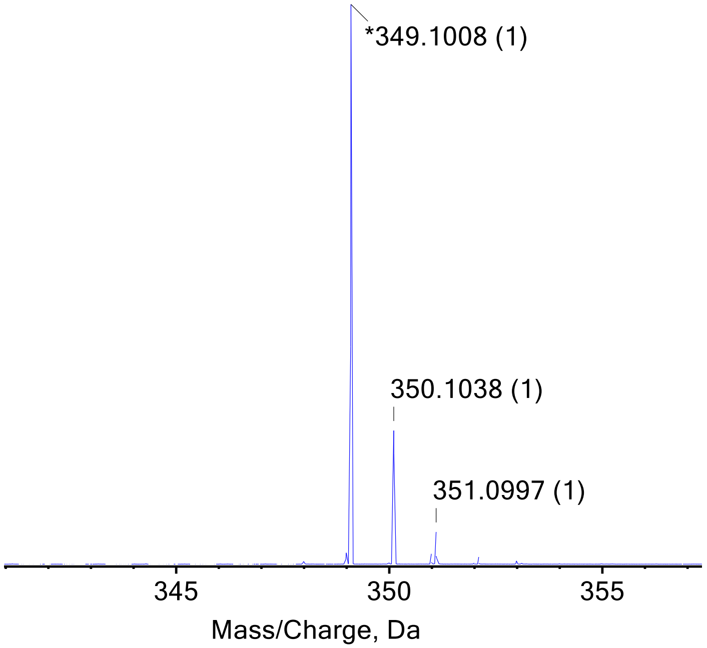

Table S1. TbBSF 90-13 growth curve data with 50 uM pyridothiazinones **1a**-**1n**. Cell densities are shown in parasites/mL in the following table. "R1" = Run 1, etc.

Hours	0	18	42	66
DMSO R1	2.33E+05	1.75E+06	5.35E+06	2.85E+06
DMSO R2	2.33E+05	2.07E+06	4.80E+06	2.15E+06
DMSO R3	2.33E+05	1.58E+06	5.30E+06	1.85E+06
DMSO R4	2.67E+05	1.10E+06	5.60E+06	2.62E+06
**1a** R1	2.33E+05	1.24E+06	3.80E+06	3.25E+06
**1a** R2	2.33E+05	1.19E+06	3.40E+06	3.60E+06
**1a** R3	2.33E+05	1.18E+06	3.10E+06	2.90E+06
**1b** R1	2.33E+05	1.36E+06	3.02E+06	3.15E+06
**1b** R2	2.33E+05	1.23E+06	3.33E+06	2.86E+06
**1b** R3	2.33E+05	1.26E+06	3.48E+06	3.23E+06
**1c** R1	2.67E+05	5.20E+05	8.10E+05	1.32E+06
**1c** R2	2.67E+05	6.50E+05	1.17E+06	1.37E+06
**1c** R3	2.67E+05	4.20E+05	9.10E+05	1.22E+06
**1d** R1	2.33E+05	0.00E+00	0.00E+00	0.00E+00
**1d** R2	2.33E+05	1.00E+04	0.00E+00	0.00E+00
**1d** R3	2.33E+05	0.00E+00	0.00E+00	0.00E+00
**1e** R1	2.33E+05	0.00E+00	0.00E+00	0.00E+00
**1e** R2	2.33E+05	1.00E+04	1.00E+04	0.00E+00
**1e** R3	2.33E+05	0.00E+00	0.00E+00	0.00E+00
**1f** R1	2.33E+05	0.00E+00	0.00E+00	0.00E+00
**1f** R2	2.33E+05	0.00E+00	0.00E+00	0.00E+00
**1f** R3	2.33E+05	0.00E+00	0.00E+00	0.00E+00
**1g** R1	2.33E+05	0.00E+00	0.00E+00	0.00E+00
**1g** R2	2.33E+05	1.00E+04	0.00E+00	0.00E+00
**1g** R2	2.33E+05	0.00E+00	0.00E+00	0.00E+00

**1h** R1	2.33E+05	1.17E+06	2.47E+06	2.80E+06
**1h** R2	2.33E+05	1.08E+06	2.73E+06	2.80E+06
**1h** R3	2.33E+05	9.70E+05	2.82E+06	2.35E+06
**1i** R1	2.33E+05	1.21E+06	2.83E+06	2.60E+06
**1i** R2	2.33E+05	9.60E+05	2.06E+06	2.55E+06
**1i** R3	2.33E+05	1.02E+06	2.06E+06	2.91E+06
**1j** R1	2.33E+05	1.40E+06	3.17E+06	2.63E+06
**1j** R2	2.33E+05	1.21E+06	3.95E+06	3.36E+06
**1j** R3	2.33E+05	1.37E+06	3.65E+06	3.69E+06
**1k** R1	2.33E+05	5.00E+04	2.00E+04	5.00E+04
**1k** R2	2.33E+05	0.00E+00	0.00E+00	0.00E+00
**1k** R3	2.33E+05	0.00E+00	0.00E+00	2.00E+04
**1l** R1	2.33E+05	5.00E+05	8.20E+05	1.35E+06
**1l** R2	2.33E+05	5.20E+05	6.50E+05	1.24E+06
**1l** R3	2.33E+05	7.00E+05	1.31E+06	1.91E+06
**1m** R1	2.33E+05	7.50E+05	1.53E+06	2.74E+06
**1m** R2	2.33E+05	1.25E+06	2.68E+06	3.09E+06
**1m** R3	2.33E+05	1.19E+06	2.27E+06	3.12E+06
**1n** R1	2.33E+05	1.39E+06	2.02E+06	2.45E+06
**1n** R2	2.33E+05	1.22E+06	2.32E+06	2.80E+06
**1n** R3	2.33E+05	1.10E+06	2.36E+06	2.77E+06
